# Basement membrane ligands initiate distinct signalling networks to direct cell shape

**DOI:** 10.1016/j.matbio.2020.02.005

**Published:** 2020-08

**Authors:** Michael J. Randles, Franziska Lausecker, Jonathan D. Humphries, Adam Byron, Simon J. Clark, Jeffrey H. Miner, Roy Zent, Martin J. Humphries, Rachel Lennon

**Affiliations:** a Wellcome Centre for Cell-Matrix Research, Division of Cell-Matrix Biology and Regenerative Medicine, School of Biological Sciences, Faculty of Biology Medicine and Health, The University of Manchester, Manchester Academic Health Science Centre, Manchester, UK; b Cancer Research UK Edinburgh Centre, Institute of Genetics and Molecular Medicine, University of Edinburgh, Edinburgh, UK; c Universitäts-Augenklinik Tübingen, Eberhard Karls University of Tübingen, Germany; d The Lydia Becker Institute of Immunology and Inflammation, Faculty of Biology Medicine and Health, The University of Manchester, Manchester, UK; e Renal Division, Washington University School of Medicine, Saint Louis, MO, USA; f Department of Medicine, Vanderbilt University Medical Center, Nashville, TN, USA; g Department of Paediatric Nephrology, Royal Manchester Children's Hospital, Manchester University Hospitals NHS Foundation Trust, Manchester Academic Health Science Centre, Manchester, UK

**Keywords:** Basement membrane, Type IV collagen, Laminin, Adhesome, Cell shape, Rac1, PKC

## Abstract

Cells have evolved mechanisms to sense the composition of their adhesive microenvironment. Although much is known about general mechanisms employed by adhesion receptors to relay signals between the extracellular environment and the cytoskeleton, the nuances of ligand-specific signalling remain undefined. Here, we investigated how glomerular podocytes, and four other basement membrane-associated cell types, respond morphologically to different basement membrane ligands. We defined the composition of the respective adhesion complexes using mass spectrometry-based proteomics. On type IV collagen, all epithelial cell types adopted a round morphology, with a single lamellipodium and large adhesion complexes rich in actin-binding proteins. On laminin (511 or 521), all cell types attached to a similar degree but were polygonal in shape with small adhesion complexes enriched in endocytic and microtubule-binding proteins. Consistent with their distinctive morphologies, cells on type IV collagen exhibited high Rac1 activity, while those on laminin had elevated PKCα. Perturbation of PKCα was able to interchange morphology consistent with a key role for this pathway in matrix ligand-specific signalling. Therefore, this study defines the switchable basement membrane adhesome and highlights two key signalling pathways within the systems that determine distinct cell morphologies. *Proteomic data are availableviaProteomeXchange with identifier* PXD017913.

## Highlights

• Cells have evolved mechanisms to sense the composition of their adhesive microenvironment and although much is known about general mechanisms employed by adhesion receptors to relay signals between the extracellular environment and the cytoskeleton, the nuances of ligand-specific signalling remain undefined.
• We investigated cellular responses to different basement membrane ligands and defined the composition of their respective adhesion complexes using mass spectrometry-based proteomics. On type IV collagen, multiple human epithelial cell types adopted a round shape with large adhesion complexes rich in actin-binding proteins.
• By contrast cells on laminin were elongated with small adhesion complexes enriched in endocytic and microtubule-binding proteins.
• Consistent with their distinctive shapes, cells on type IV collagen exhibited high Rac1 activity, while those on laminin had elevated PKCα and perturbation of either Rac1 or PKCα caused cell shape interchange.
• Overall this study defines ligand-specific adhesomes and highlights two key signalling hubs that influence cell shape.

## 1 Introduction

Adhesion to extracellular matrices has a profound effect on cell morphology, proliferation and migration. Since interstitial matrices, basement membranes (BMs) and other extracellular assemblies support specialized functions, it follows that cells have evolved mechanisms to sense the composition of their adhesive microenvironment. However, while much is now known about the general mechanisms employed by adhesion receptors to relay signals between the extracellular environment and the cytoskeleton, ligand-specific signalling is not fully understood.

Type IV collagen and laminin isoforms are core BM components and due to associations with human disease, they have been extensively investigated in the kidney glomerulus. This structure consists of specialized capillaries lined by glomerular endothelial cells and covered by epithelial podocytes. These two cell types engage the intervening glomerular BM and together this specialized capillary wall forms the kidney filtration barrier. During development, the glomerular BM is rich in laminin α5β1γ1 (laminin 511) and type IV collagen α1α1α2, whereas laminin α5β2γ1 (laminin 521) and type IV collagen α3α4α5 isoforms predominate in the mature glomerulus [1,2].

Podocytes adhere to the BM via integrins, leading to the recruitment of a diverse range of adaptor and signalling molecules to integrin cytoplasmic tails. These integrin adhesion complexes (IACs) tether the podocyte cytoskeleton to the underlying matrix and provide a mechanism for podocytes to sense and interpret the integrity of the BM. Several analytical approaches have been used to elucidate the proteins that comprise IACs when cells adhere to fibronectin-rich matrix substrates. At least 232 proteins have been robustly identified using targeted, candidate-based approaches [3,4] and many more using global mass spectrometry (MS)-based proteomic approaches [[5], [6], [7], [8]]. Using MS, IACs have been characterized at the level of activation[9], phosphorylation [10], and integrin receptor isoform specificity [6,11]. Recent integration of global proteomic datasets has enabled the definition of a consensus adhesome of core adhesion machinery comprising 60 components [12]. However, these studies have not included BM ligands; therefore, we aimed to investigate the influence of BM ligand on IAC composition and the associated consequences for cellular signalling and morphology.

Interpretation of matrix substrate is critical for cells. Both matrix and integrins are differentially expressed throughout human tissues, and specific matrix-integrin interactions are important for the function of distinct tissues [13]. Furthermore, mutations in adhesion signalling and BM components have a profound impact on cell function [14,15]. One example is Pierson syndrome, a rare form of congenital kidney disease: these patients have severe proteinuria and podocyte abnormalities caused by mutations in *LAMB2* [16]. A second example is Alport syndrome, caused by *COL4A3*, *COL4A4* or *COL4A5* mutations in humans, which leads to progressive loss of kidney function associated with sensory neuronal hearing loss [[17], [18], [19]]. Recent studies have shown that podocytes usually adhere to laminin in the normal glomerular BM, whereas in Alport syndrome podocytes make contact with ectopic type IV collagen α1α1α2, potentially disrupting normal podocyte adhesion signalling [20]. We therefore selected the podocyte as a BM ligand-responsive cell type to study differences in IAC composition on distinct BM ligands.

We analysed podocyte responses to type IV collagen and laminin (511 and 521) and we observed distinct cell shapes and signalling. Furthermore, we confirmed the same ligand-dependent changes in morphology in four other BM-associated cell types. We proceeded to analyze IACs using MS-based proteomics and identified BM ligand-dependent adhesion complexes characterized by the pivotal components Rac1 and PKCα, which could be manipulated to effect BM ligand-dependent morphologies.

## Results

### Basement membrane ligand determines cell shape

**Fig. 1:**
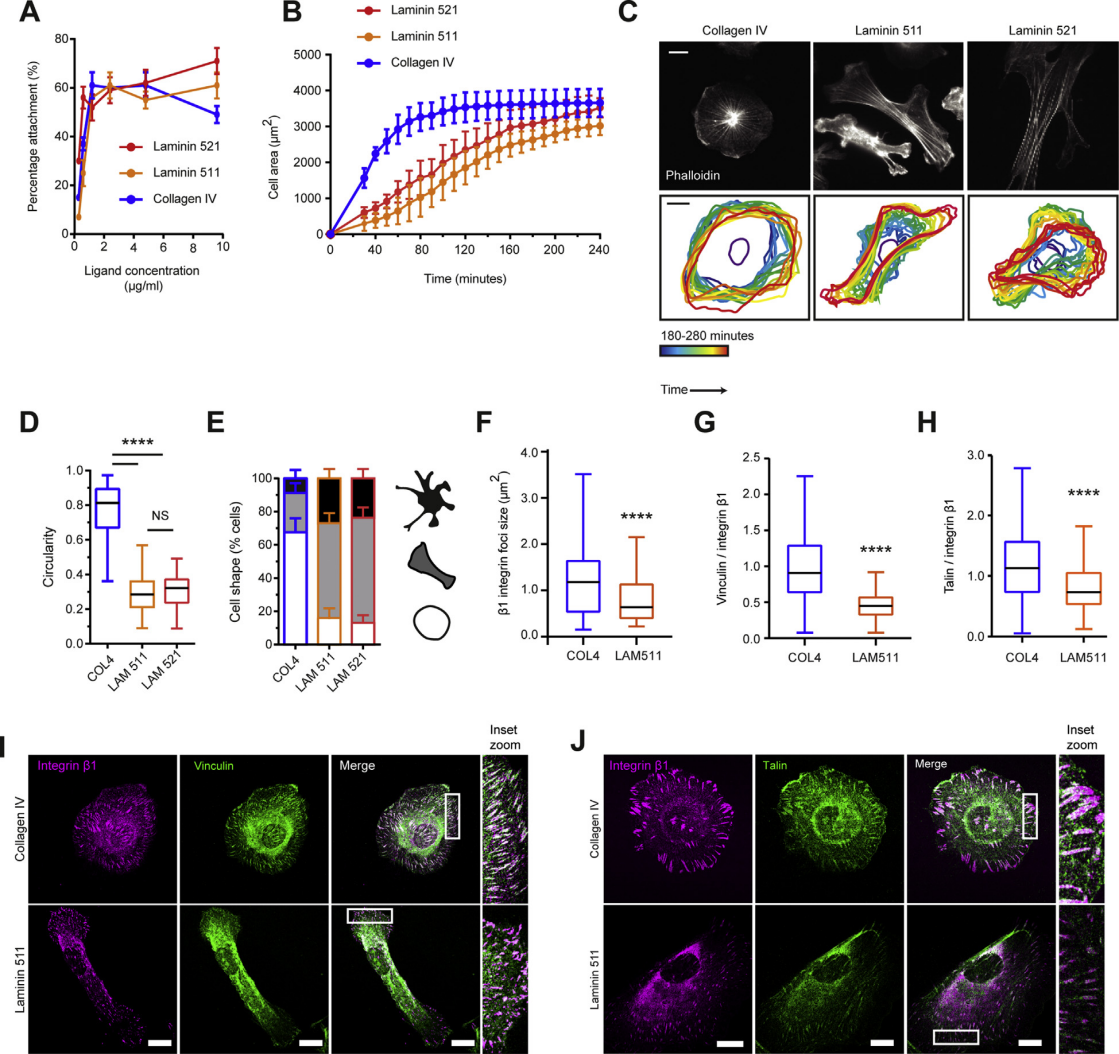
Adhesion to basement membrane ligand determines cellular morphology. (A) Podocytes were allowed to attach to plates coated with 0–10 μg/ml of matrix substrate for 30 min in serum-free media. Non-attached cells were removed by washing with PBS, and the percent of added cells attached to the substrate was quantified by crystal violet staining. (B) Podocytes attached to 5 μg/ml of matrix substrate for 240 min in serum-free media; collagen IV, laminin 511 and laminin 521. Cell spread area was calculated using phase-contrast live cell imaging. Measurements of cell area were extracted using Fiji ImageJ. (C–J) Podocytes were spread on 5 μg/ml of matrix substrate for 210 min in serum-free media. (C) Phalloidin staining of podocytes highlights distinct cellular shapes and actin structures within podocytes adhered to collagen IV compared with laminin. The colour-coded shape outlines indicate representative protrusive activities at 5-min intervals recorded between 180 and 280 min of cell spreading. (D) Podocytes circularity assessment when attached to collagen IV compared with laminin. Circularity was calculated using Fiji ImageJ. (E) Cell morphology was assessed using Fiji ImageJ; cells were manually categorised as rounded elongated or containing multiple protrusions. Podocytes had more elongated shapes and also produced more pseudopodial protrusions when spread on laminin compared with collagen IV. (F) Integrin β1 foci were larger in podocytes spread on collagen IV compared with podocytes spread on laminin. (G–H) Ratio imaging demonstrated differential localization of (G) vinculin and (H) talin to integrin adhesion complexes formed on collagen IV and laminin. (I–J) Immunofluorescence of Integrin β1 and (I) vinculin and (J) talin in podocytes spread on collagen IV or laminin. For attachment and spreading assays, experiments were performed four times. For immunofluorescence and morphology-determining experiments, 20–40 cells were measured per experiment and each experiment was performed four times. (I, J) Scale bar represents 10 μm; ∗∗∗∗, *p* < 0.0001; NS, not significant; LAM511, laminin-511; LAM521, laminin-521; COL4, collagen IV. Bar and line graph measurements are shown as mean ± standard deviation. Box plots indicate 25th and 75th percentiles (lower and upper bounds, respectively), 1.5 × interquartile range (whiskers) and median (black line). (For interpretation of the references to colour in this figure legend, the reader is referred to the Web version of this article.)

To study cell shape responses to BM ligands, human podocytes were allowed to attach and spread on type IV collagen α1α1α2 (collagen IV), laminin 511 (which predominates during glomerular development) or laminin 521 (the main isoform in the mature BM). Cells attached to all three ligands at low concentrations (Fig. 1A), but spreading occurred more rapidly on collagen IV (Fig. 1B). On all three substrates, however, podocytes reached the same average spread cell area within 210 min (Fig. 1B). The laminin receptor α3β1 integrin and the tightly-associated tetraspanin CD151 were highly expressed on the podocyte cell surface as determined by flow cytometry (Supplementary Fig. 1A). In addition, we observed differential levels of expression of phosphorylated paxillin (Y118) compared to β1 integrin on collagen IV and laminin, suggesting distinct integrin adhesion complexes (Supplementary Fig. 1B).

Irrespective of the degree of spreading, collagen IV and laminin induced different cell shapes. On type IV collagen, podocytes spread, but adopted a circular shape and were rarely polarized. The cells exhibited radial actin stress fibres and a single lamellipodium that often encompassed the entire cell edge (Fig. 1C–E). These cells also formed large β1 integrin-, vinculin- and talin-positive integrin adhesion complexes (IACs; Fig. 1F–J). In contrast, podocytes spread on laminin 511 or 521 were elongated and polygonal with a large number of pseudopodial projections (Fig. 1C–E) and smaller IACs (Fig. 1F–J). Under all conditions, there was no significant difference in cell responses to the two different laminin isoforms, consistent with both heterotrimers sharing the same integrin-binding domain in the α5 chain [[21], [22], [23], [24], [25]]. For this reason, the majority of subsequent experiments compared collagen IV with laminin 511.

We also examined cell shape on fibronectin as this matrix ligand localizes to BMs in certain circumstances and it is also abundant in cell culture. Podocytes on fibronectin reached a similar spread area to cells on laminin (Supplementary Fig. 1C and D) but they were more rounded. Furthermore, we compared cell area with the addition RGD peptide at a concentration to inhibit fibronectin engagement with α5β1 or αvβ3 integrins. Cell spreading on fibronectin was reduced significantly, whilst the area of podocytes on collagen IV and laminin was preserved following RGD peptide treatment (Supplementary Fig. 1C and D). This indicates that the BM ligands laminin and collagen IV were dominant in the experimental system we used.

To test the generality of the morphological response to collagen IV and laminin, a retinal pigment epithelial cell line (ARPE19), glomerular endothelial cells (GEnCs) and HEK293T cells were examined. *In vivo*, retinal pigment epithelial cells contact Bruch's membrane, which has a similar composition to the glomerular BM [[26], [27], [28]]. All three cell types showed morphological dependence on ligand, with laminin inducing polarization and protrusion, and type IV collagen leading cells to adopt circular morphologies (Supplementary Fig. 2 A-F). We further assessed the impact of adhesion to collagen IV or laminin on cellular phenotype using a dorsal root ganglion (DRG) neurite outgrowth assay. Podocytes express a number of neuronal markers, and podocyte branching and foot process formation are thought to employ similar guidance cues to that of neurite outgrowth [[29], [30], [31]]. DRG outgrowth occurred on both collagen IV and laminin, but the average length of neurites on laminin was significantly greater than those generated on type IV collagen (Supplementary Fig. 2G and H), again demonstrating BM influence on cellular phenotype.

Thus, for a range of cell types, adhesion to collagen IV induces a rounded phenotype, while laminin 511 supports a protrusive phenotype. Although β1 integrins mediate adhesion to both ligands, these findings imply both compositional differences in the associated IACs and differences in signal transduction to the cytoskeleton.

### Matrix ligand and cell morphology influence cellular signalling

**Fig. 2:**
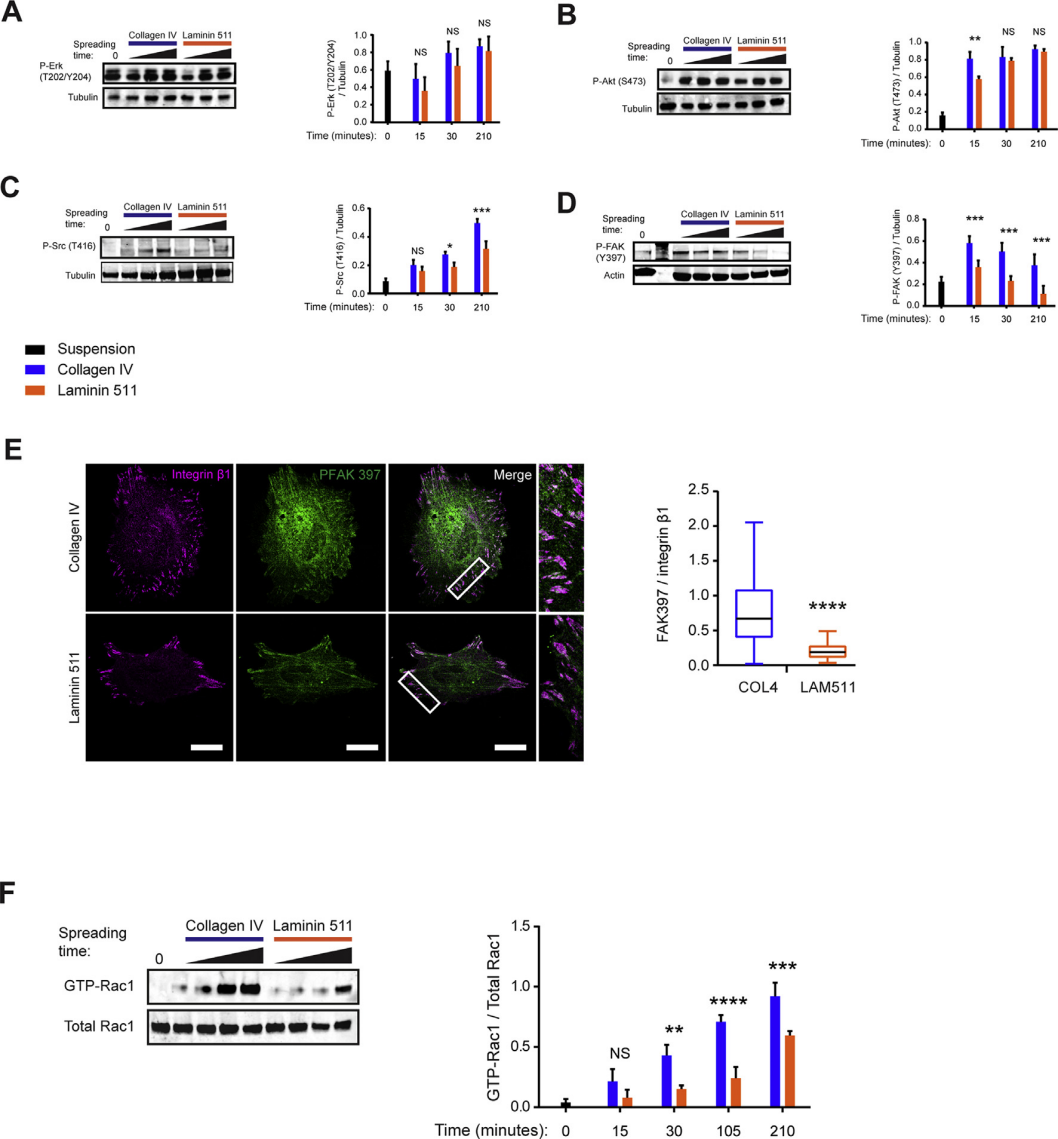
Signalling response to basement membrane (BM) ligands. (A–D) Canonical integrin signalling assayed by Western blot of total cell lysates from podocytes spreading on BM ligands over a 210-min time course of cells spread on 5 μg/ml of matrix substrate. (A–D) Podocyte P-Erk (T202/Y204), P-Akt (S473), P-Src (T416) and P-FAK (Y397) levels at 0, 15, 30 and 210 min of cell spreading onto either type IV collagen or laminin 511. Band intensity was calculated using integrated density in Fiji imageJ. (E) Immunofluorescence and ratio imaging quantification of P-FAK (Y397) and Integrin β1 in podocytes after 210 min of cell spreading onto BM ligands. For these experiments 20 cells were measured per experiment and each experiment was performed four times (F) GST-PAK pull down of active Rac1 assayed in podocytes spreading in serum-free media on 5 μg/ml of matrix substrate over a 210-min time course (0, 15, 30, 105 and 210 min of cell spreading). All bar graph measurements are shown as mean ± standard deviation. Box plots indicate 25th and 75th percentiles (lower and upper bounds, respectively), 1.5 × interquartile range (whiskers) and median (black line). Scale bar in (E) represents 10 μm; ∗∗∗∗, *p* < 0.0001; NS, not significant; LAM511, laminin-511; COL4, collagen IV.

To gain a greater understanding of the link between cell morphology, protrusive activity and BM ligand, we assessed canonical integrin signalling. AKT and ERK1/2 were activated in podocytes during cell spreading on collagen IV and laminin ligands to the same degree (Fig. 2A–B). In contrast, Src and FAK activation were both enhanced on collagen IV compared with laminin (Fig. 2C–D). These differences were also observed with immunofluorescence microscopy, where the amount of phosphorylated FAK (Y397) localized to IACs was increased on collagen IV compared with laminin (Fig. 2E). The phosphorylated form of another canonical integrin signalling protein, paxillin Y118, was also enriched in collagen IV IACs compared with laminin IACs (Supplementary Fig. 1B). Since podocytes spread on collagen IV had an appearance consistent with hyperactivated Rac1, we quantified Rac1 activity using PAK1 pulldown. Rac1 activity was significantly increased in podocytes spread on collagen IV compared with laminin (Fig. 2F).

**Fig. 3:**
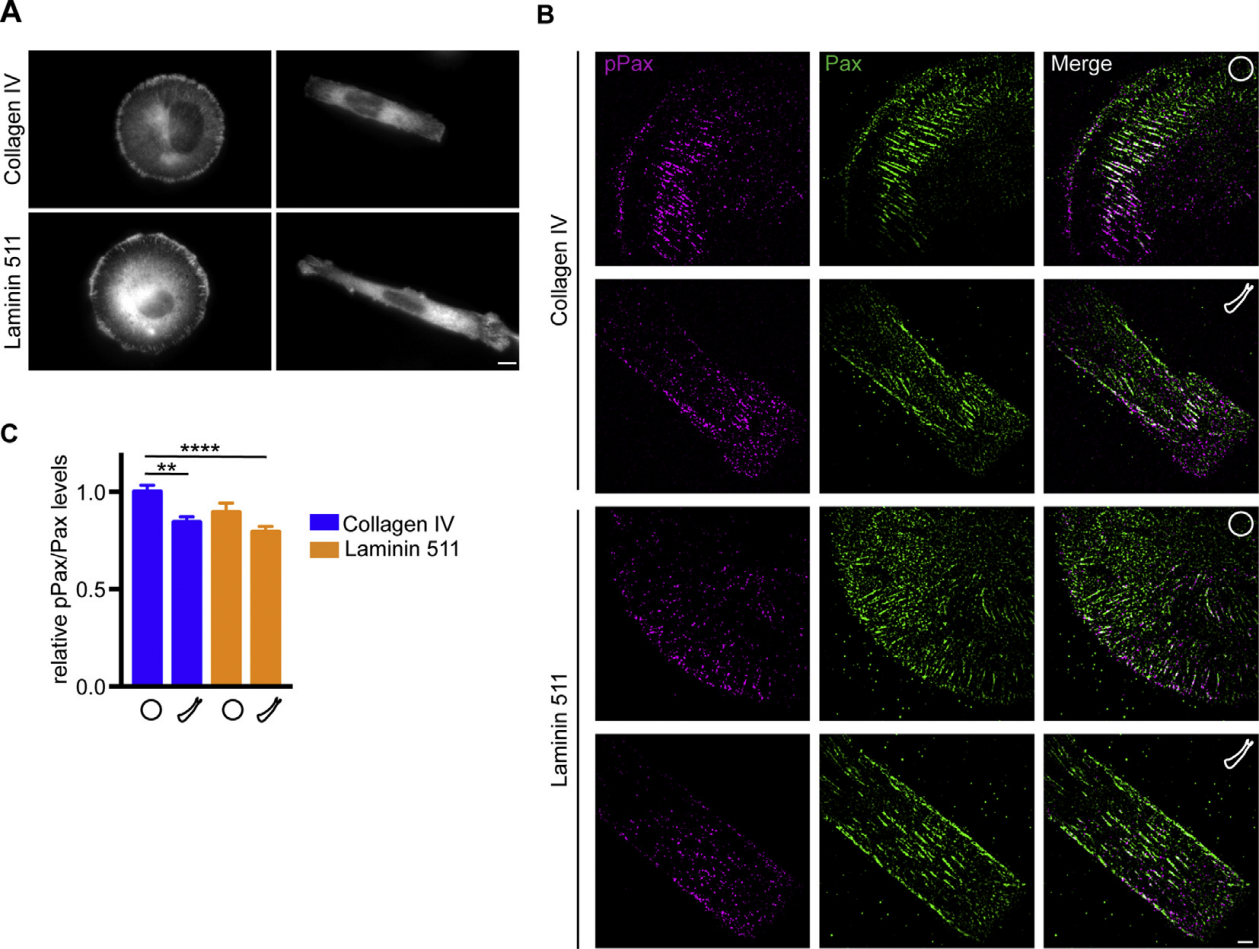
Focal adhesion signalling on micropatterned surfaces. (A) Micropatterned circles and lines with an area of 2800 μm^2^ were prepared and coated with collagen IV or laminin 511 (both at 5 μg/ml). Podocytes were spread on the micropatterned surfaces for 3.5 h in serum-free media. Podocytes adapted the round and elongated cell shapes on both ligands and formed paxillin-positive adhesions. Scale bar represents 10 μm. (B) Cells were stained for Paxillin and phospho-paxillin (Y118). Scale bar represents 2 μm. (C) Ratiometric imaging was performed on adhesions, quantifying phosphorylated paxillin (Y118) levels relative to paxillin levels. Data are pooled from three independent experiments; 40–60 cells were analysed per condition; ∗∗∗∗, *p* < 0.0001; ∗∗, *p* < 0.01.

Once a cell engages with a BM ligand, its shape is altered, and our data in Fig. 1, Fig. 2 demonstrate that there is differential signalling on BM ligands. To determine whether the BM ligand or the resulting cell morphology had the predominant influence on focal adhesion signalling, we spread podocytes on micropatterned circles or lines and examined phosphorylated paxillin (Y118) specifically at focal adhesions (Fig. 3A). Podocytes on collagen IV circles had the highest phospho-paxillin to paxillin ratio, whereas podocytes on laminin lines had the lowest ratio and there was a gradient between (Fig. 3B–C and Supplementary Fig. 2I). This suggests that, even when a particular shape is enforced, the BM ligand has a dominant effect on focal adhesion signalling.

### The composition of the adhesome is matrix ligand-dependent

To investigate how adhesion to collagen IV or laminin influences adhesion signalling and cell shape, we isolated basolateral IAC using established methods [32] from podocytes spread onto BM substrates. Podocytes were allowed to attach to collagen IV, laminin-511 or laminin-521 and form IACs. Subsequently, IACs were stabilized using a reversible crosslinker and podocyte cell bodies and nuclei removed by detergent lysis and hydrodynamic force (Supplementary Fig. 3A). The purity of the matrix ligands was assessed by MS. Collagen IV, laminin 511 and laminin 521 all demonstrated a high degree of purity (Supplementary Fig. 3B).

IAC marker proteins talin and vinculin were detected in isolated IACs, but proteins that do not localize to IACs, such as Bcl-2 homologous antagonist/killer (BAK) and heat shock 70 kDa protein (HSP70), were not found (Supplementary Fig. 3D and E). Apotransferrin was used as a negative control for proteins that are basolateral but not IAC-associated. Cells attached to apotransferrin, but did not spread and remained phase bright (Supplementary Fig. 3F). Talin and vinculin were not detected in these complexes by western blotting (Supplementary Fig. 3D), while MS analysis revealed a high abundance of the transferrin receptor and low abundance of integrin receptors in apotransferrin complexes (Supplementary Fig. 3C). In contrast, integrins were enriched in IACs and included the β1, α1, α2, α3, α5 and αV subunits. These data suggest that podocytes utilise αVβ1 to a similar degree when spreading on BM ligands and this may reflect the presence of fibronectin in cell culture. In contrast, integrin α3β1 is utilized to a greater degree by podocytes when spreading on laminin, whereas podocytes enlist a combination of α1β1, α2β1 and α5β1 to a greater degree when spreading on type IV collagen (Supplementary Fig. 3C, Supplementary Table 1).

**Fig. 4:**
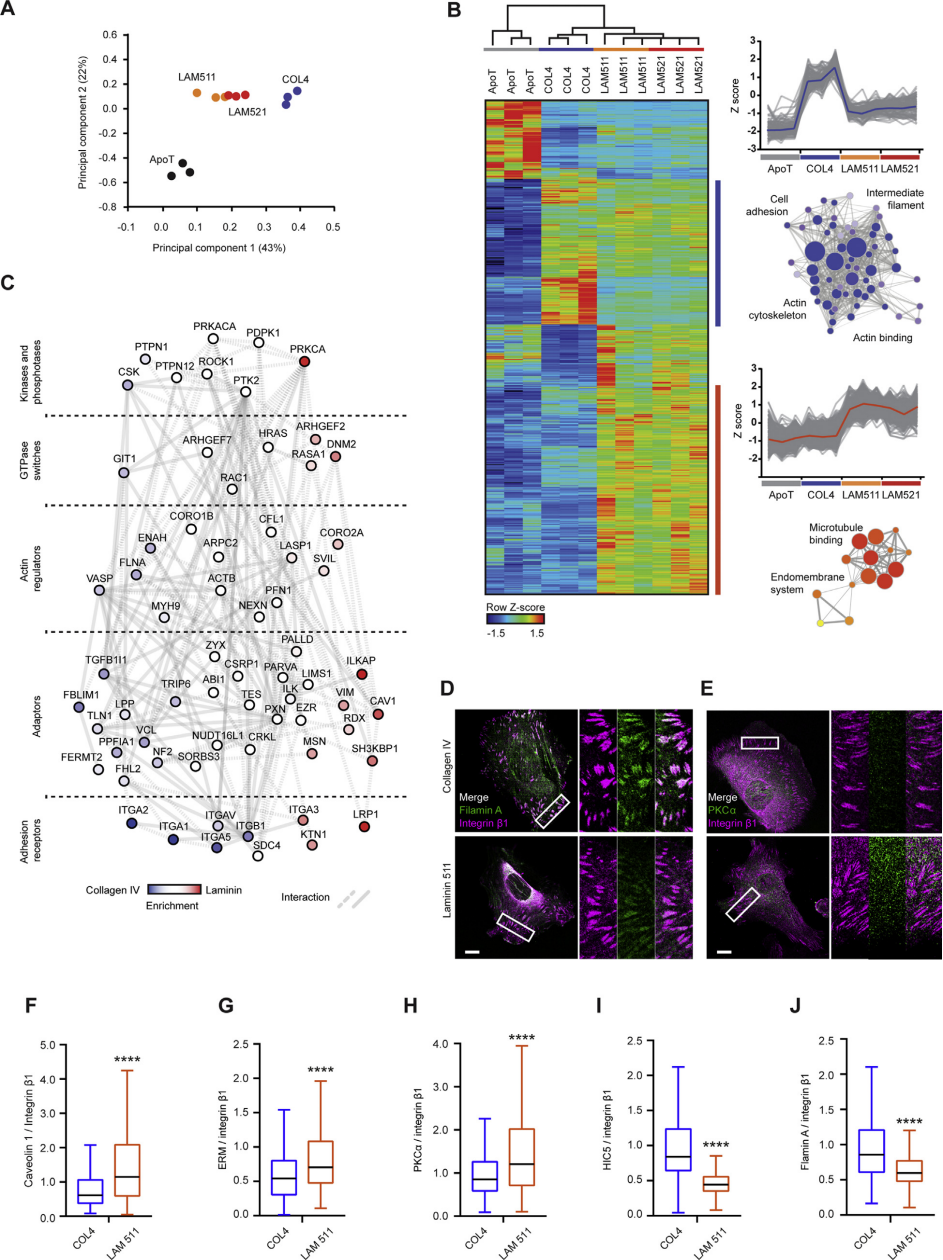
The adhesome is matrix ligand dependent. (A) Principal component analysis of mass spectrometry data from podocytes. Circles represent biological replicates. (B) Unsupervised hierarchical clustering analysis of integrin adhesion complexes (IACs). Clustering was performed on the basis of Euclidian distance. The heat map displays row z-score, plotted in hot–cold colour scale, with hot (red-orange) indicating high abundance and cold (green-blue) indicating low abundance. Summary protein profiles across conditions are displayed. Grey lines show individual protein profiles in the highlighted cluster and the central coloured line represents the mean protein abundance of the cluster. Associated Gene Ontology enrichment maps demonstrate enriched biological processes. The size of the nodes (circles) represents the number of proteins, the grey lines (edges) between the nodes represents overlap of proteins within biological process terms. The intensity (opacity) of colouring represents the *p* value of the enrichment. (C) Protein-protein interaction network constructed from known integrin adhesion components that were identified by mass spectrometry. Proteins are sorted into different categories. Edges represent protein-protein interactions, nodes represent proteins. Solid edges represent reported protein-protein interactions detected using multiple different techniques, dashed lines represent lower confidence interactions detected with single techniques. Coloured nodes represent those with significantly different abundance in laminin (511 and 521 combined) IACs compared with collagen IV IACs. (D) Immunofluorescence of filamin A and integrin β1 in cells spread on matrix-ligand for 210 min. (E) Immunofluorescence of PKCα and integrin β1 in cells spread on matrix-ligand for 210 min. (F–J) Ratio imaging analysis of integrin adhesion components identified as either laminin or collagen IV enriched by MS. For immunofluorescence and morphology-determining experiments, 20–40 cells were measured per experiment and each experiment was performed four times. Box plots indicate 25th and 75th percentiles (lower and upper bounds, respectively), 1.5 × interquartile range (whiskers) and median (black line). Scale bar in (D, E) represents 10 μm; ∗∗∗∗, *p* < 0.0001; NS, not significant; LAM511, laminin-511; COL4, collagen IV; ApoT, apotransferrin. (For interpretation of the references to colour in this figure legend, the reader is referred to the Web version of this article.)

Principal component analysis and hierarchical clustering highlighted the relationship between replicates, with collagen IV IACs and laminin IACs grouping separately, suggesting heterodimer-specific components are present in laminin and collagen IV IACs (Fig. 4A–B). In keeping with previous experiments, laminin 511 and laminin 521 IACs were largely indistinguishable, with only the laminin β1 and β2 changed in abundance between the two conditions (Fig. 4A–B, Supplementary Table 1). Gene Ontology enrichment analysis revealed that actin-binding proteins were enriched in collagen IV IACs, whereas microtubule-binding proteins and endocytic machinery components were enriched in laminin IACs (Fig. 4B).

### The basement membrane adhesome comprises consensus adhesome components

MS analysis of IACs was also performed in ARPE19 cells. In general, there was congruence between the two datasets (Supplementary Fig. 4A, Supplementary Table 2). In terms of both number and abundance of proteins, the type IV collagen adhesome resembled more closely the known fibronectin consensus adhesome, with most of the less well connected and characterized consensus components enriched in laminin IACs (Supplementary Fig. 4C, Supplementary Tables 3 and 4). In order to focus on known IAC components, we generated protein-protein interaction networks using the consensus adhesome (Supplementary Fig. 4C) and literature-curated adhesome (Fig. 4C). Interestingly, we identified a number of Rac1-interacting proteins that were differentially recruited to IACs dependent on BM ligand (Supplementary Fig. 3D). Filamin A and IQGAP1, which interact to suppress Rac1 activity, were enriched in collagen IV IACs [33]. In contrast, RCC2, also known to suppress Rac1 activity [8], was enriched in laminin IACs. These data suggest that, dependent on the adhesion type, different pathways are utilized by cells to curtail Rac1 activity.

We validated the findings from MS using immunofluorescence (Fig. 4D–J, Supplementary Fig. 5A). In the MS dataset, filamin A and Hic5 were enriched in collagen IV IACs, whereas caveolin-1 and ezrin, radixin, moesin (ERM) proteins were enriched in laminin IACs: the patterns of enrichment were confirmed by fluorescence imaging and ratio imaging quantification (Fig. 4D–J, Supplementary Fig. 5A). Furthermore, myosin-1E localized to IACs and was more abundant in laminin IACs than collagen IV IACs (Supplementary Table 5, Supplementary Fig. 5B). Myosin-1E is of particular interest as mutations in *MYO1E* cause nephrotic syndrome [34] and it has also been implicated as a genetic modifier in Alport syndrome [35]. We also identified ARHGAP29 in podocyte IACs and validated this by immunofluorescence (Supplementary Fig. 5C). Both ARHGAP29 and myosin-1E localized to circular adhesions that form underneath the nucleus (Supplementary Fig. 5B and C). EPB41L3 was also detected in podocyte IACs and was equally abundant in both collagen IV and laminin IACs (Supplementary Table 5).

### PKCα adhesion signalling is matrix ligand-dependent

**Fig. 5:**
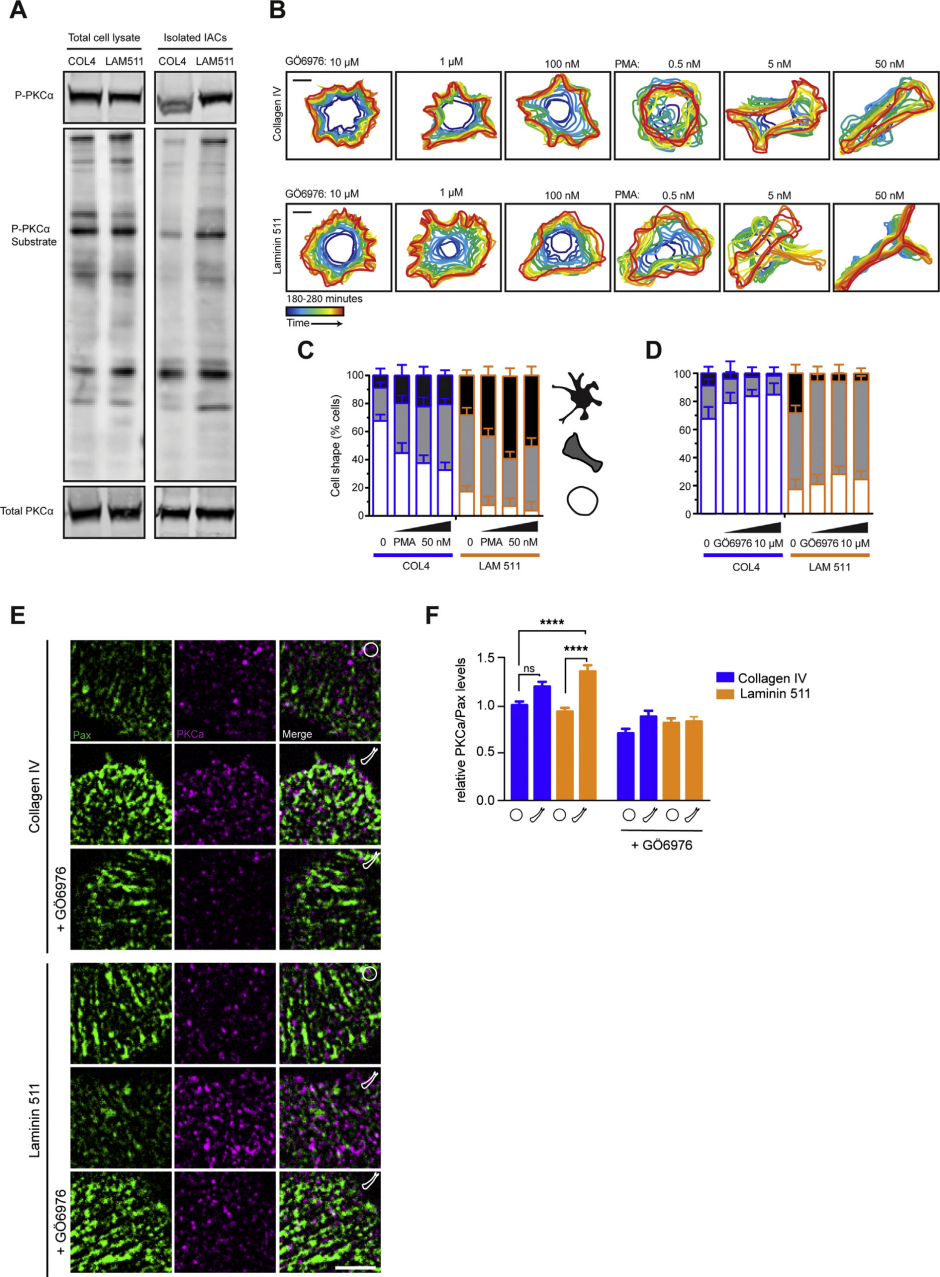
Modulating PKC alters cell morphology and signalling. (A) Western blot demonstrates PKC activity in both total cell lysates and integrin adhesion complexes from podocytes spread onto collagen IV or laminin for 210 min. (B) The colour-coded shape outlines indicate representative protrusive activities at 5-min intervals recorded over 100-min period. For measurements of protrusive activity live cell imaging was performed between 180 and 280 min of cell spreading. Podocytes were treated with either GӦ6976 or PMA to suppress or induce PKC activity respectively during cell spreading on matrix ligand. (C,D) PKC modulation impacts podocyte morphology and pseudopodial projection formation. (E,F) PKCα localization to adhesions is controlled through ligand specificity. Podocytes were spread on circles and lines coated with collagen IV or laminin for 3.5 h in serum-free media and stained for PKCα and paxillin and ratiometric imaging was performed. Scale bar represents 20 μm. Data are pooled from three independent experiments. GӦ6976 induced inhibition of PKCa localization to FA was significant for collagen circles, lines and laminin lines. For ratiometric imaging 20–40 cells were analysed per experiment and each experiment was performed four times. All bar graph measurements are shown as mean ± standard deviation. Box plots indicate 25th and 75th percentiles (lower and upper bounds, respectively), 1.5 × interquartile range (whiskers) and median (black line). Scale bar in (e) represents 5 μm; ∗∗∗∗, *p* < 0.0001; NS, not significant; LAM511, laminin-511; COL4, collagen IV. (For interpretation of the references to colour in this figure legend, the reader is referred to the Web version of this article.)

Although consensus adhesome proteins were enriched in IACs formed on collagen IV, many kinases and phosphatases were enriched in laminin IACs (Supplementary Table 4). Protein kinase C alpha (PKCα) was highly enriched in laminin IACs (Fig. 4C, E, H). We therefore investigated the role of PKCα during adhesion to BM ligands. PKC activity in total cell lysates from cells adhered to collagen IV or laminin was similar, whereas PKC activity was enhanced in isolated laminin IACs compared with collagen IV IACs (Fig. 5A). The role of PKCα in influencing cellular morphology was tested by altering PKC signalling using phorbol 12-myristate 13-acetate (PMA) as an activator of PKC or GӦ6976 as an inhibitor. Inhibition of PKC induced cells on laminin to become modestly rounded, phenotypically similar to those spreading on collagen IV, whereas activating PKC in cells spreading on collagen IV induced the cells to produce more pseudopodial projections and resemble cells spreading on laminin (Fig. 5B–D). To examine the role of cell shape in directing ligand-mediated signalling, PKCα levels were investigated in the adhesions of cells spread on collagen IV and laminin micropatterns. In line with our previous findings, highest PKCα levels were observed at adhesions on laminin lines and less PKCα localization to adhesions on collagen circles. Spreading podocytes on laminin circles decreased localization of PKCα to adhesions, suggesting a potential contribution of the cell morphology towards cellular signalling, which was abolished upon GӦ6976-induced inhibition of PKCα (Fig. 5E and F). Furthermore, treatment with PKCα modulators had effects on both the actin and microtubule cytoskeleton during cell adhesion (Supplementary Fig. 6A). The role of PKC signalling in matrix interpretation was also tested in DRG neurite outgrowth assays. Inhibition of PKC using GӦ6976 reduced neurite length in cells on laminin, but had no effect on collagen IV (Supplementary Fig. 6B). Conversely, PMA enhanced neurite length on collagen IV at 8 h, but this was not seen with laminin (Supplementary Fig. 6B).

### Integrin α3 and PKCα cooperate in laminin matrix interpretation

**Fig. 6:**
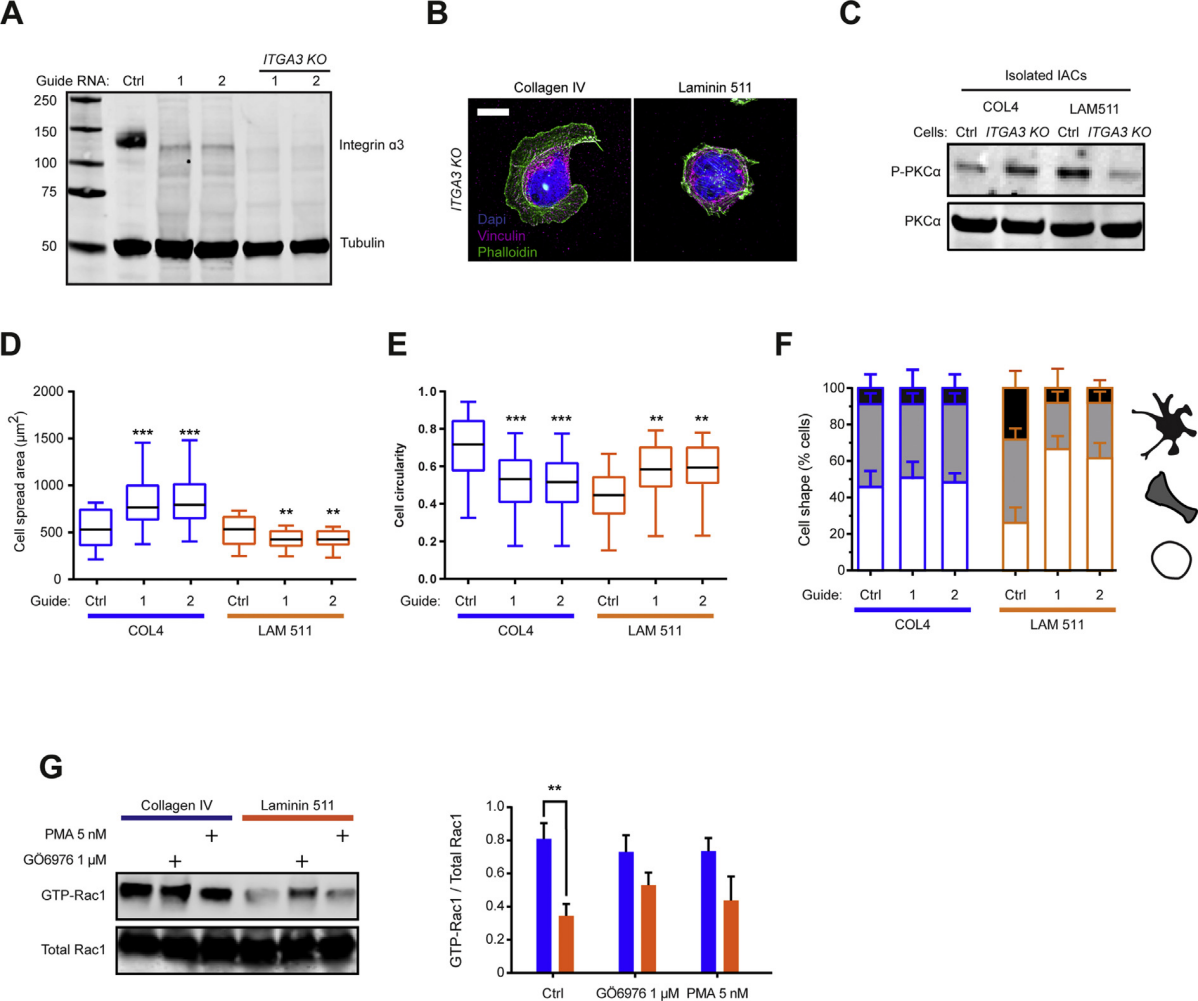
Integrin α3 and PKC are involved in matrix interpretation. (A) Deletion of *ITGA3* from HEK293T cells using two different CRISPR-Cas9 guide RNAs. Ctrl, scrambled guide RNA. Lane 1, molecular weight marker; lane 2, control guide RNA; lane 3, *ITGA3* guide RNA 1 targeted cells prior to fluorescent activated cell sorting; lane 4, *ITGA3* guide RNA 2 targeted cells prior to fluorescent activated cell sorting; lane 5, *ITGA3* guide RNA 1 targeted cells after fluorescent activated cell sorting; lane 6, *ITGA3* guide RNA 1 targeted cells after fluorescent activated cell sorting. (B) Immunofluorescence of *ITGA3-KO* cells spread onto matrix ligand for 210 min. (C) Western blot demonstrates PKC activity in integrin adhesion complexes from *ITGA3-KO* cells spread onto matrix ligand for 210 min. (D–F) Deletion of *ITGA3* impacts cell morphology and pseudopodial projection formation. (G) GST-PAK pull down of active Rac1 assayed in podocytes spreading on BM ligands over a 210-min time course. All bar graph measurements are shown as mean ± standard deviation. For immunofluorescence and morphology-determining experiments, 20–40 cells were measured per experiment and each experiment was performed four times. All bar graph measurements are shown as mean ± standard deviation. Box plots indicate 25th and 75th percentiles (lower and upper bounds, respectively), 1.5 × interquartile range (whiskers) and median (black line). Scale bar in (f) represents 10 μm; ∗∗∗∗, *p* < 0.0001; NS, not significant; LAM511, laminin-511; COL4, collagen IV.

As the integrin α3 subunit was the only integrin receptor that we identified enriched in laminin IACs (Fig. 4C, Supplementary Fig. 3C and Supplementary Table 1), we explored the relationship between integrin α3, PKCα and cell morphology. Using CRISPR-Cas9, *ITGA3*-null (*ITGA3-KO*) cells were generated (Fig. 6A) and spreading assays on collagen IV or laminin were performed. *ITGA3-KO* cells still attached to laminin, but had reduced spreading, formed fewer pseudopodial projections and had reduced PKCα signalling in isolated IACs (Fig. 6B–F). When *ITGA3-KO* cells adhered to collagen IV, the cells had increased cell area and there was also an effect on PKCα signalling at IACs (Fig. 6B–F). Interestingly, the *ITGA3-KO* cells plated onto collagen IV displayed an opposing phenotype to cells plated onto laminin, with increased cell spread area, PKCα signalling and decreased circularity, and this could indicate utilization of alternative integrin heterodimers. Finally, Rac1 activity in podocytes during spreading on collagen IV was not significantly influenced by either PKC activation or inhibition. In contrast, during cell spreading on laminin, podocyte Rac1 activity was modestly increased by PKC inhibition but not influenced by PKC activation. This suggests alternative mechanisms of Rac1 inhibition dependent on IAC composition (Fig. 6G). Overall, these findings suggest that an integrin α3 to laminin interaction influences PKC signalling, and this pathway promotes pseudopodial generation and an elongated cell shape.

## Discussion

In this investigation, we have defined the adhesome of cells engaging with basement membrane ligands. We discovered that: 1) laminin-based adhesion signalling led to elongated cells with multiple extended pseudopodial projections, reminiscent of foot process structures formed by podocytes *in vivo*; 2) type IV collagen-based adhesion induced circular cell phenotypes associated with hyperactivated Rac1; and 3) these differential responses to BM ligand were not unique to podocytes but also identified in retinal pigment epithelial cells, glomerular endothelial cells, HEK293T and neurites. Although previous studies have described laminin and collagen ligands inducing different cell morphologies [36], this study is the first to substantiate these observations with detailed MS-based investigation of IACs and the definition of key cell signalling components responsible for determining cell shape (PKCα and Rac1).

BMs underlie all sheets of cells and are composed of condensed networks of matrix proteins. Core BM constituents include type IV collagen, laminin isoforms, nidogens and heparan sulfate proteoglycans; however, MS-based proteomic studies have revealed the complex nature of BMs and have identified tissue-specific expression of hundreds of BM components [37]. Tissue-specific expression is likely to represent the variable functional roles of BMs from a semi-permeable membrane (in the kidney filter, blood-brain barrier and the blood-retinal barrier) to an impermeable structural scaffold (in the skin or intestine). Cells adhere to BMs via IACs yet it is unclear which BM ligand cells preferentially engage. The nanoscale architecture of the glomerular BM was investigated using correlative STORM-EM imaging [20] and these data suggest that, in health, podocytes and endothelial cells engage laminin rather than type IV collagen and this engagement is switched in kidney diseases such as Alport syndrome [20]. This study also localized type IV collagen to the middle of the healthy glomerular BM, perhaps indicating a stabilising role upon fusion of the endothelial and podocyte BMs during development [38].

In this study, we observed striking differences in the morphologies of cells on laminin and type IV collagen. These morphological patterns were observed in several cell types that engage BM ligands (glomerular podocytes and endothelial cells, HEK293 cells, retinal pigment epithelial cells and neuronal cells), suggesting that the interpretation of BM ligand is conserved between cell types. The morphological patterns we observed may also relate to pathological changes *in vivo*. In the kidney, healthy podocytes have large cell bodies with multiple cellular extensions or foot processes that cover the surfaces of the glomerular capillaries and interdigitate with processes from neighbouring cells. In this situation, podocytes engage laminin at the cell-matrix interface [20]; however, in glomerular disease, podocytes undergo a considerable change in morphology and become effaced and flattened. Although this morphological switch has been recognized for decades, the associated changes in signalling are not well understood. In the effaced state, podocytes may be exposed to alternative matrix ligands, including ectopic type IV collagen, and this ligand switch could drive outside-in signalling to effect altered morphology.

To gain insight into the signalling pathways involved in the morphological switch, we used MS-based proteomics and demonstrated that podocyte IAC composition is dependent on BM ligand. Actin-binding machinery was enriched in collagen IV IACs, whereas microtubule-binding and endocytic machinery were enriched in laminin adhesion complexes. This is in keeping with studies that have shown the importance of laminin to integrin β1 interactions promoting microtubule assembly and stabilization in axons [39]. This observation suggests that collagen IV adhesion complexes form more robust links to the actin cytoskeleton thereby generating more tension and enhanced Rho family GTPase activity. In contrast, MS data suggest that laminin adhesion complexes may be hubs for kinase signalling and microtubule regulation, and that they may also be more readily turned over. Focusing on specific candidates from the MS analysis, we validated myosin-1E and ARHGAP29 as novel IAC components, which also localized to atypical adhesion structures. We also identified PKCα enrichment in laminin IACs and further study suggested integrin α3 and PKCα signalling downstream of laminin is an important determinant of cell morphology in cells attached to laminin. Accordingly, upon integrin α3 knockout, cells spreading on laminin showed decreased PKCα activation and acquired a round shape. In contrast, the knocked-down cells plated on collagen IV displayed an opposite phenotype, with increased PKCα activation; higher cell spread area and reduced cell circularity. We speculate that this opposing phenotype could be linked to increased available β1 integrin forming alternative heterodimers in the absence of α3. We further speculate that cells spreading on collagen are able to utilise these alternative heterodimers more effectively than cells spreading on laminin.

In conclusion, we have defined the composition of IACs in cells engaging BM ligands and have identified key signalling pathways acting through these protein complexes to effect changes in cell shape. Further investigation of how differential integrin-ligand binding triggers different signalling pathways could lead to strategies to switch cell morphology, which may have therapeutic implications for a range of human diseases.

### Experimental procedures

#### Antibodies

Antibodies used were against mouse anti-human paxillin (clone 349; BD Biosciences), mouse anti-human CD151 (11G5a; Abcam), rat anti-human integrin α6 (GoH3; Abcam), rat anti-human integrin β1 (9EG7), mouse anti-human caveolin 1 (clone 2297; BD Biosciences), mouse anti-human integrin α1 (FB12; Millipore), mouse anti-human ezrin/radixin/moesin (EMD Millipore), mouse anti-human integrin α2 (P1E6; Abcam), mouse anti-human integrin α3 (IA3 clone; ab20141; Abcam) (P1B5; ab24696; Abcam), goat anti-human integrin α3 (I-19 Santa Cruz Biotechnology), mouse anti-human Rac1 (BD Biosciences), mouse anti-human integrin α4 (HP2/1; Abcam), mouse anti-human integrin αvβ3 (LM609; Millipore), mouse polyclonal vinculin (hVin1; Sigma-Aldrich), polyclonal rabbit anti-human p (Y397)FAK (44-624G; Invitrogen), rabbit anti-human BAK (B5897; Sigma-Aldrich), rabbit anti-human filamin A (H-300; Santa Cruz Biotechnology), rabbit anti-human Hic5 (Thermo Fisher Scientific; PA528839), rabbit anti-human PKCα (Cell Signalling Technology) (P4334; Sigma-Aldrich), rabbit anti-human p (T638)PKCα (44962G; Thermo Fisher Scientific), rabbit anti-human phospho-(Ser) PKC substrate (Cell Signalling Technology), rabbit anti-human myosin 1E (HPA023886; Sigma), rabbit anti-human ARHGAP29 (sc-365554; Santa Cruz Biotechnology). Alexa Fluor 488 Phalloidin molecular probe was used to detect actin filaments and tubulin was detected with α-tubulin rat monoclonal antibody (YL1/2; Thermo Fisher Scientific). Secondary antibodies conjugated to Alexa Fluor 488 or 594 (Life Technologies) were used for immunohistochemistry; secondary antibodies conjugated to Alexa Fluor 680 (Life Technologies) or IRDye 800 (Rockland Immunochemicals) were used for western blotting.

#### Cell culture

Conditionally immortalized human podocytes [40] were grown in uncoated tissue culture plates. Podocytes between passage 10 and 16 were cultured for 14 days at 37 °C in RPMI 1640 medium with glutamine (R-8758; Sigma-Aldrich) supplemented with 10% (v/v) foetal calf serum (FCS) (Life Technologies) and 5% (v/v) ITS (I-1184; Sigma-Aldrich; 1 ml/100 ml). Glomerular endothelial cells (GEnCs) were grown in endothelial basal medium-2 (CC-3156; Lonza) containing 5% (v/v) FCS and EGM-2 BulletKit growth factors (CC-4147; Lonza), excluding VEGF, for 5 days at 37 °C. Retinal pigment epithelial (ARPE19) cells were grown in uncoated tissue culture plates. ARPE19 cells between passage 16 and 20 were cultured at 37 °C in 50% DMEM4, 50% Ham's F12 medium supplemented with 10% (v/v) FCS. HEK293T cells were cultured at 37 °C in DMEM4 medium supplemented with 10% (v/v) FCS. Two integrin α3 KO HEK293T cell lines were generated by CRISPR-Cas9. CRISPR-Cas9 plasmids containing gRNAs against *ITGA3* guide 1 (5′- TCGGGTATCCAGGTTGAAGG-3) and *ITGA3* guide 2 (5′-TCCCGGCCTCCTTCACTACC-3′) were acquired from Horizon Discovery (8100 Cambridge Research Park). Cells were transiently transfected with constructs containing the gRNA and Cas9 Nickase. Forty-eight hours after transfection, cells were sorted using fluorescence-activated cell sorting with an anti-integrin α3 extracellular domain antibody (IA3 clone; ab20141; Abcam). Integrin α3-negative cells were collected and single cells were sorted into individual wells of a 96-well plate. Single-sorted cells were allowed to expand and deletion of integrin α3 was verified by immunoblotting using a goat anti-human integrin α3 antibody (I-19; Santa Cruz Biotechnology).

#### Isolation of dorsal root ganglia

Dorsal root ganglion neurons (DRGs) were isolated using a previously described method [41]. Briefly, following euthanasia, the vertebral column of adult mice was dissected to collect all of the dorsal root ganglion neurons (DRGs). DRGs were cleaned to remove the roots and connective tissue from ganglia, and incubated with 1.25% collagenase diluted in Hams F12 for 45 min at 37 °C and 5% CO_2_. The solution was then removed and replaced with fresh 1.25% collagenase diluted in Hams F12 and incubated for a further 45 min at 37 °C and 5% CO_2_. Ganglia were washed gently 3 times with Hams F12 and dissociated using a glass pipette. The suspension was then passed through a sterile 70-μm mesh and centrifuged at 300 rpm for 5 min. The supernatant was discarded and the neurones resuspended in 0.5 ml Hams F12. The resulting cell suspension was then layered on top of a 15% solution of bovine serum albumin (fatty acid free, Sigma) diluted in Hams F12. Finally, the sample was centrifuged at 900 rpm for 10 min. The supernatant was discarded including the debris layer at the interface, and the cells were resuspended in media Hams F12 medium containing NGF, (Sigma cat. no. N6009), 1% N2, (2 μM progesterone (Sigma cat. no. P8783), 10 mM putrescine (Sigma cat. no. P5780), 10 mg/ml transferrin (Sigma cat no. T8158), 3 μM sodium selenite (Sigma cat. no. S5261) and 1% AraC.

#### Flow cytometry

For standard flow cytometric analysis, podocytes were washed with phosphate-buffered saline without cations (PBS-) and detached from culture surfaces with 1× trypsin-EDTA at 37 °C. The dissociated cells were harvested by centrifugation (1000 rpm, 4 min). The cell pellet was resuspended in 0.1% (w/v) BSA/0.1% (w/v) sodium azide in PBS- (0.1/0.1 solution). Cells were then incubated with primary antibody, diluted in 0.1/0.1 solution, at 4 °C for 30 min. Following two washes with 0.1/0.1 solution and centrifugation steps, cells were incubated with appropriate species-specific FITC-conjugated secondary antibody at 4 °C for 30 min. Cells were then washed three times with 0.1/0.1, resuspended in PBS- and analysed on a Dako CYAN, FACS machine.

#### Matrix ligands

Recombinant human laminin-511 and laminin-521 were a kind gift from Professor Karl Tryggvason, and can be purchased from BioLamina. Fibronectin from bovine plasma (F1141) and type IV collagen from human placenta (C5533) was purchased from Sigma-Aldrich.

#### Activators and inhibitors

RGD peptide (Cyclo (-Arg-Gly-Asp-D-Phe-Val) trifluoroacetate salt) was purchased from BACHEM (4026200). Drug titration experiment revealed most impaired cell spreading on fibronectin at 10 mM. In subsequent spreading assays the RGD peptide was used at 10 mM. PMA (phorbol 12-myristate 13-acetate (PMA) was purchased from Sigma-Aldrich and used at 0.5–50 nM to activate PKC. GӦ6976 was purchased from Sigma-Aldrich and used at 100 nM-10 μM to inhibit PKC.

#### Attachment assay

For all cell adhesion assays, cells were trypsinized for 10 min, followed by pipetting cells up and down to break up cell clumps. Cells were left to trypsinize for another 5 min, and then pelleted by centrifugation (1000 rpm for 4 min at room temperature). To remove cell-bound extracellular matrix, cells were washed three times with PBS-. Cells were left to incubate in serum-free RPMI 1640 (podocytes) or 50% DMEM, 50% Ham's-F12 (ARPE19) for 30 min at 37 °C and 5% (v/v) CO2 to downregulate ECM adhesion signalling events. Subsequently, cells were pelleted and washed with serum-free RPMI 1640 (podocytes) or serum-free DMEM5 and spread on tissue culture dishes coated with ECM ligand (0–10 μg/ml) or apotransferrin (10 μg/ml). For quantification percentage of cell attachment, 96-well plates were blocked with 10 mg/ml heat-denatured BSA solution followed by coating with the matrix molecule of interest diluted to the appropriate concentration in DPBS. Cells were allowed to attach to matrix-coated wells for 30 min at 37 °C. Nonadherent and loosely attached cells were removed by gently washing the wells with 100 μL of PBS. Cells were fixed in the wells by addition of 100 μL 5% (w/v) glutaraldehyde for 20 min at room temperature. Subsequently, cells were stained with 100 μL 0.1% (w/v) crystal violet, 200 mM MES, pH 6.0, for 60 min at room temperature. Wells were washed three times with dH_2_O. Finally, dye was solubilized in 100 μL 10% (v/v) acetic acid for 5 min at room temperature and absorbance recorded at 570 nm using a plate reader. To estimate a value for 100% attachment, cells were added directly to uncoated plastic in medium containing serum for 30 min and fixed without washing. Assays were performed in quadruplicate.

#### Micropatterned surfaces

Micropatterned surfaces were created according to manufacturer's instructions (Primo Alveole). In brief, glass-bottom dishes (MatTek Corporation) were coated with a poly (l)-lysine poly ethylene glycol (PEG) layer (PLL-PEG) (Alveole). Addition of a photo initiator (PLPP) (Alveole) and UV-light exposure of the area of interest (circles and lines) degraded the polymer and made the area of interest available for coating with collagen IV and laminin. Circles and lines had an area of 60 μm^2^. Leonardo software (Alveole) was used to upload the patterning designs and an Eclipse Ti inverted microscope (Nikon) using a 20×/0.45 SPlan Fluar objective was used to expose the dishes to UV-light. Micropatterned surfaces were coated with laminin 511 and collagen IV (both at 5 μg/ml) and podocytes were spread on the surfaces for 3.5 h in serum-free media.

#### IAC isolation

IACs were isolated using a similar approach to the ligand affinity purification method described previously [32]. For isolation of IACs from podocytes or ARPE19 cells, cells were treated in the same manner as for the attachment assay described above. Cells were spread on tissue culture dishes coated with ECM ligand (2.5–10 μg/ml) or apotransferrin (10 μg/ml) for 180 min following attachment assay described above. Thereafter, cells were incubated with the membrane-permeable crosslinker dimethyl-3,3′-dithiobispropionimidate (DTBP; Sigma-Aldrich; 6 mM) diluted in Advanced Dulbecco's Modified Eagle's Medium (DMEM5) (Sigma-Aldrich) for 3 min and quenched with Tris-HCl, pH 8.5, after which cells were washed twice with PBS. Cell bodies were removed by a 1-min incubation with extraction buffer (10 mM Tris, 150 mM NaCl, 1% (v/v) Triton X-100, 25 mM EDTA, 25 μg/ml leupeptin, 25 μg/ml aprotinin and 0.5 mM AEBSF). Denuded cells were subjected to 30 s high-pressure water wash to removed nuclei. Protein complexes left bound to the substrate were collected in reducing sample buffer (50 mM Tris-HCl, pH 6.8, 10% (w/v) glycerol, 4% (w/v) sodium dodecylsulfate (SDS), 0.004% (w/v) bromophenol blue, 8% (v/v) β-mercaptoethanol) and incubated at 70 °C for 10 min. Adhesion complex samples were fractionated by SDS-PAGE and used either for western blotting or visualized with InstantBlue to be used for in-gel proteolytic digestion.

#### MS data acquisition

Following SDS–PAGE, gel lanes were sliced and subjected to in-gel digestion with trypsin [42] with modifications [8]. Peptide samples were analysed by liquid chromatography (LC)-tandem MS using a nanoACQUITY UltraPerformance LC system (Waters) coupled online to an LTQ Velos mass spectrometer (Thermo Fisher Scientific) or using an UltiMate 3000 Rapid Separation LC system (Thermo Fisher Scientific) coupled online to an Orbitrap Elite mass spectrometer (Thermo Fisher Scientific). Peptides were concentrated and desalted on a Symmetry C_18_ preparative column (20 mm × 180 μm, 5-μm particle size; Waters) and separated on a bridged ethyl hybrid C_18_ analytical column (250 mm × 75 μm, 1.7-μm particle size; Waters) using a 45-min linear gradient from 1% to 25% or 8%–33% (v/v) acetonitrile in 0.1% (v/v) formic acid at a flow rate of 200 nl min^−1^. Peptides were selected for fragmentation automatically by data-dependent analysis. The mass spectrometry proteomics data have been deposited to the ProteomeXchange Consortium via the PRIDE [43] partner repository with the dataset identifier PXD017913.

#### MS data analysis

Tandem mass spectra were extracted using extract_msn (Thermo Fisher Scientific) executed in Mascot Daemon (version 2.4; Matrix Science). Peak list files were searched against a modified version of the Uniprot mouse database (version 3.70; release date, May 3, 2011), containing ten additional contaminant and reagent sequences of non-mouse origin, using Mascot (version 2.2.06; Matrix Science) (Perkins et al., 1999). Carbamidomethylation of cysteine was set as a fixed modification; oxidation of methionine and hydroxylation of proline and lysine were allowed as variable modifications. Only tryptic peptides were considered, with up to one missed cleavage permitted. Monoisotopic precursor mass values were used, and only doubly and triply charged precursor ions were considered. Mass tolerances for precursor and fragment ions were 0.4 Da and 0.5 Da, respectively. MS datasets were validated using rigorous statistical algorithms at both the peptide and protein level [44,45] implemented in Scaffold (version 3.6.5; Proteome Software). Protein identifications were accepted upon assignment of at least two unique validated peptides with ≥90% probability, resulting in ≥99% probability at the protein level. These acceptance criteria resulted in an estimated protein false discovery rate of 0.1% for all datasets.

#### MS data quantification

Relative protein abundance was calculated using peptide intensity. Orbitrap MS data were entered into Progenesis LC-MS (Non Linear Dynamics Ltd) and automatically aligned. Spectra were extracted using extract_msn executed in Mascot Daemon (version 2.4; Matrix Science) and imported back into Progenesis LC-MS to acquire intensity data. Alignment of chromatograms was carried out using the automatic alignment algorithm, followed by manual validation and adjustment of the aligned chromatograms. All features were used for peptide identifications. Progenesis LC-MS created the peak list file that was exported and searched in Mascot. Results were loaded in Scaffold (Proteome Software Inc, version 3.6.5) and peptide and protein identification thresholds were set to 95% and 99% confidence, respectively. Data were exported from Scaffold as a spectrum report, and imported into Progenesis LC-MS to assign peptide identifications to features. Peptide and protein data were then exported from Progenesis LC-MS as.csv files to be analysed in Excel (Microsoft).

#### Protein interaction network analysis

Protein interaction network analysis was performed using Cytoscape (version 2.8.1) [46]. Proteins identified in at least two biological replicates were mapped onto a merged human, mouse and rat interactome built from Protein Interaction Network Analysis platform *Homo sapiens* network (release date, December 10, 2012), *Mus musculus* network (release date, December 10, 2012) and the *Rattus norvegicus* network (release date, December 10, 2012) [47], the ECM interactions database MatrixDB (release date, April 20, 2012) [48], and a literature-curated database of integrin-based adhesion–associated proteins [49]. For networks where enrichment is presented, Progenesis LC-MS normalized intensity data were used. Topological parameters were computed using the Network Analyzer plug-in [50].

#### Immunofluorescence and image analysis

Cells on coverslips were washed with PBS and then fixed with 4% (w/v) paraformaldehyde. Cells were permeabilized with 0.5% (v/v) Triton X-100 and blocked with 3% (w/v) BSA in PBS before incubation with primary antibodies. Coverslips were mounted and images were collected using a CoolSnap HQ camera (Photometrics) and separate DAPI/FITC/Cy3 filters (U-MWU2, 41001, 41007a, respectively; Chroma, Olching, Germany) to minimise bleed-through between the different channels. The images were collected using a Coolsnap HQ (Photometrics) camera with a Z optical spacing of 0.2 μm. Images collected were viewed and analysed with Fiji [51]. To calculate the area of immunostained marker per cell, images were edited to remove additional cells and artefacts outside the required cell area, compiled to form a hyperstack, background subtracted using a rolling ball radius of 30, ‘threshold’ function used to select immunostained marker and to convert images to black-white binary images, and ‘analyze particles' function used to calculate total black pixel area. Some images were acquired on a Delta Vision (Applied Precision) restoration microscope using a 60× objective and the [Sedat] filter set (Chroma [89000]).

#### Ratiometric image analysis

Images were acquired on a Delta Vision Core (Applied Precision) restoration microscope using a 60×/1.42 Plan Apo objective and the Sedat filter set (Chroma 89000). Images were acquired using a CSU-X1 spinning disc confocal (Yokagowa) on a Zeiss Axio-Observer Z1 microscope with a 60× and 100×/1.40 Plan-Apochromat objective, Evolve EMCCD camera (Photometrics) and motorised XYZ stage (ASI). The 488 nm and 561 nm lasers were controlled using an AOTF through the laserstack (Intelligent Imaging Innovations (3I)) allowing both rapid ‘shuttering’ of the laser and attenuation of the laser power. To analyze focal adhesions, background was subtracted using the rolling ball function. Cells were co-immunostained with paxillin (Mus) and phospho paxillin Y118 (Rabbit) or PKCα (Rabbit) antibodies and images were acquired using the same exposure time between channels. Focal adhesions (20–25 per cell) were manually selected for analysis by drawing regions of interest (ROIs). ROIs were used to measure the integrated density of the FAs in both channels. A ratio of the integrated density between both channels was calculated as described before [52].

#### Immunoblotting

Following SDS-PAGE, resolved proteins were transferred to nitrocellulose membrane (Whatman). Membranes were washed and incubated with antibodies as described previously [8]. Briefly, membranes were blocked with casein blocking buffer (Sigma-Aldrich) and probed with primary antibodies diluted in blocking buffer containing 0.05% (v/v) Tween 20. Membranes were washed with Tris-buffered saline plus Tween 20 and incubated with species-specific fluorescent dye–conjugated secondary antibodies diluted in blocking buffer containing 0.05% (v/v) Tween 20. Membranes were washed in the dark and then scanned using the Odyssey infrared imaging system (LI-COR Biosciences) to visualize bound antibodies.

#### GST-PAK pull-down

Active Rac1 was purified from lysates using a pull-down approach with GST-PAK beads. Cells were prepared as described for attachment assays prior to plating on 5 μg/ml matrix-coated dishes. At various times, 0, 15, 30 and 210 min of cell spreading, cells were lysed in ice-cold lysis buffer [20 mM Hepes, pH 7.5, 1% (wt/vol) Igepal, 0.5% (wt/vol) sodium deoxycholate, 140 mM NaCl, 4 mM EDTA, 4 mM EGTA, 10% (wt/vol) glycerol), and lysates were clarified by centrifugation at 12,000 g, 4 °C, for 1 min. Lysates were then incubated with GST-PAK beads for 1 h at 4 °C. Beads were washed three times with ice-cold lysis buffer as described above, and active GTPase was eluted off beads by addition of reducing sample buffer. Samples were then resolved by SDS-PAGE and analysed by western blotting.

#### Statistical analysis

Data presented as bar graphs or box-and-whisker plots were analysed by Shapiro-Wilk test for normality and analysed by Kruskal-Wallis test or ANOVA with post-hoc Bonferroni correction as appropriate. Principal component analysis was performed in MATLAB. Unsupervised hierarchical clustering was performed in T4MeV using a Euclidean distance-based complete-linkage matrix.

## Conflicts of interest

The authors declare no conflicts of interests.
