# Supplementary material for: Basement membrane ligands initiate distinct signalling networks to direct cell shape
Source: Matrix Biol. 2020 Aug;90:61–78. doi: 10.1016/j.matbio.2020.02.005 (PMC7327512; doi:10.1016/j.matbio.2020.02.005)
Supplement: Multimedia component 1 [file mmc1.pdf]

Supplementary Figure 1

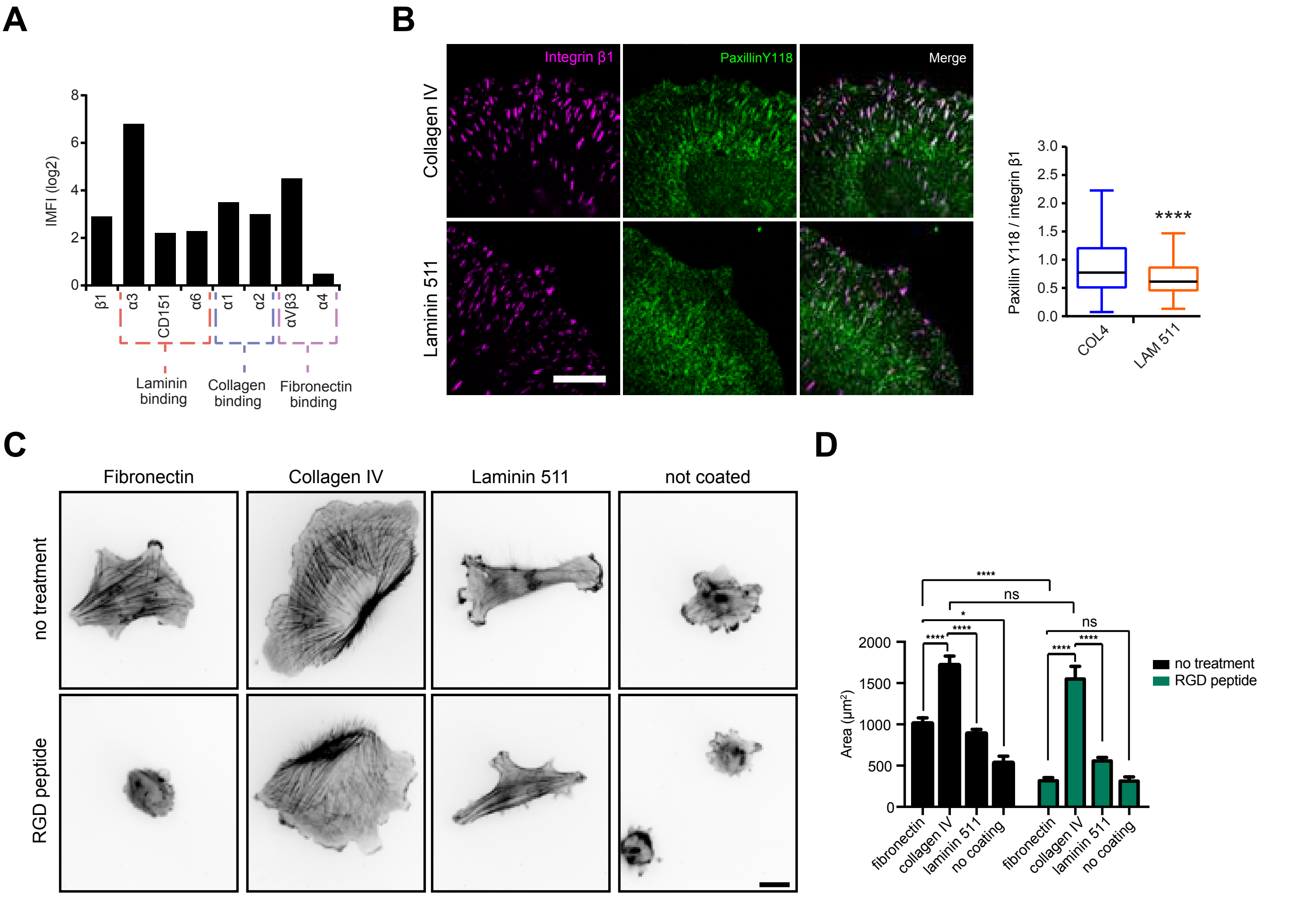

Supplementary Figure 1: (A) Podocytes express: integrin $\alpha 3\beta 1$, which binds specifically to laminin; CD151, a tetraspanin that is associated with integrin $\alpha 3\beta 1$; integrin $\alpha 1\beta 1$, which binds to laminin and collagen with preference for collagen IV; integrin $\alpha 2\beta 1$, which binds to laminin and collagen with preference for collagen I; and integrin $\alpha V\beta 3$, which binds to fibronectin, vitronectin, von Willebrand factor, latency associated peptide-transforming growth factor beta, fibrillin, fibrinogen, thrombospondin, tenascin and osteopontin. (B) Immunofluorescence and ratio imaging demonstrated differential localization of phosphorylated paxillin (Y118) to collagen IV and laminin 511 integrin adhesion complexes. For immunofluorescence and morphology determining experiments 20-40 cells were measured per experiment and each experiment was performed four times. ****, $p < 0.0001$; NS, not significant; LAM511, laminin-511; COL4, collagen IV. MFI, median fluorescence intensity. Scale bar represents 2 μm in (B). (C) Podocytes were spread on fibronectin, collagen IV and laminin for 3.5h in serum-free media with or without RGD peptide (10mM). Cells were fixed and stained for actin. Scale bar is 10 μm. (D) Quantification of C. Cell area was measured. Experiment was performed three times, for each experiment 20-50 cells were analysed. ****, $p < 0.0001$; *, $p < 0.05$; ns, not significant.

Supplementary Figure 2

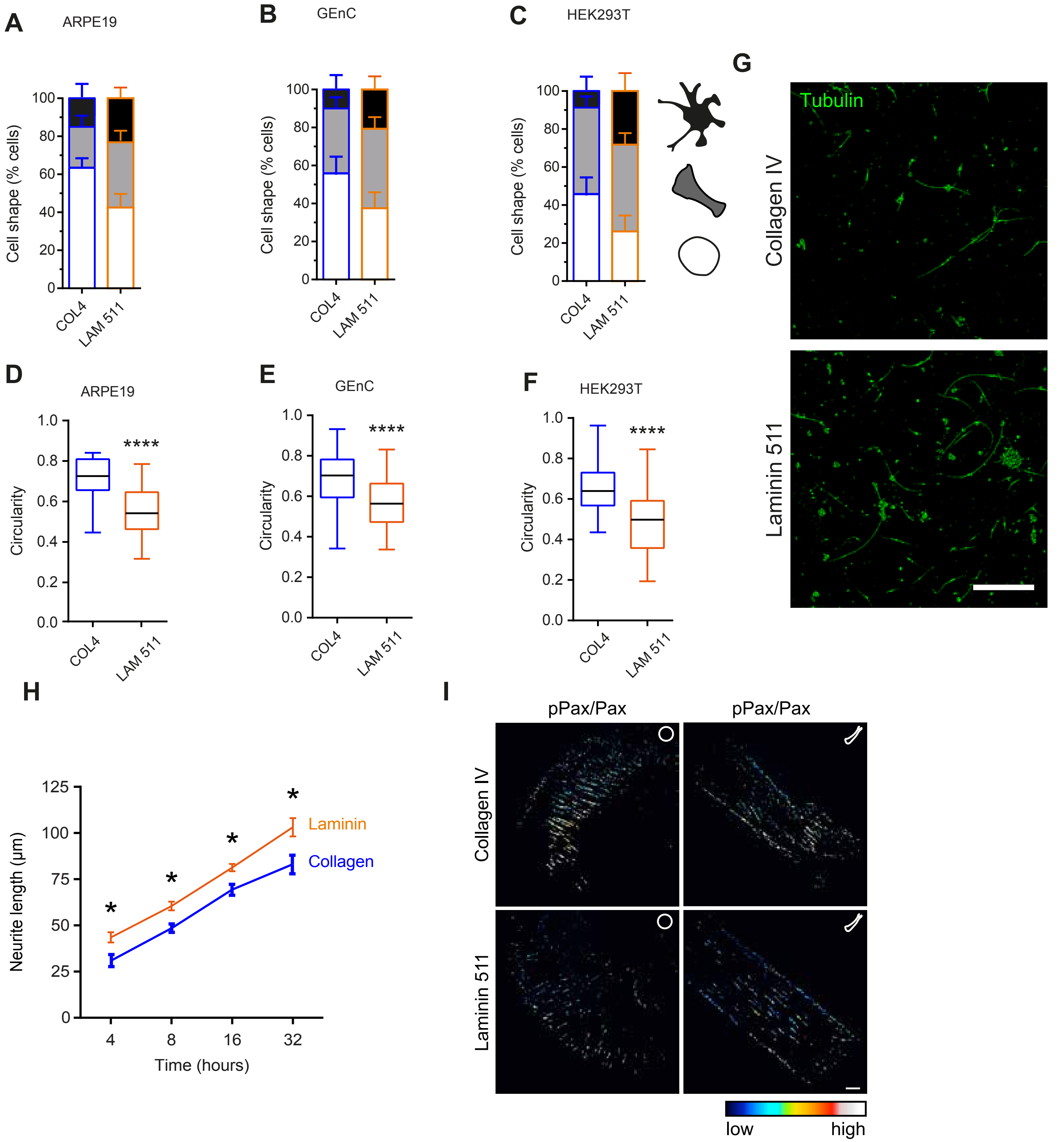

Supplementary Figure 2: Cell morphology on basement membrane ligands was conserved between cell types. (A-C) Cell morphology in response to collagen IV or laminin 511 was assessed in (A) ARPE19 cells, (B) GEnCs and (C) HEK cells. (D-F) Cell circularity and morphology in response to collagen IV or laminin 511 was assessed in (A) ARPE19 cells, (B) GEnCs and (C) HEK cells. (G-H) Dorsal root ganglion neurite out growth was assessed on collagen IV or laminin and length of outgrowth was compared using fluorescence imaging. For immunofluorescence and morphology determining experiments 20-40 cells were measured per experiment and each experiment was performed four times; (G) scale bar represents 200 µm; ****, $p < 0.0001$; LAM511, laminin-511; COL4, collagen IV; All bar graph measurements are shown as mean ± standard deviation. Box plots indicate 25th and 75th percentiles (lower and upper bounds, respectively), 1.5× interquartile range (whiskers) and median (black line). (I) Podocytes were spread on micropatterned circles or lines coated with Collagen IV and Laminin 511 (5 µg/ml) for 3.5h in serum-free media and stained for phospho-paxillin and paxillin. Ratiometric imaging was performed on adhesions, quantifying phosphorylated paxillin Y118 levels relative to paxillin levels. Yellow/red/white areas indicate high phospho-paxillin levels, whilst blue areas indicate low levels of pospho-paxillin specifically at focal adhesions. Scale bar represents 2 µm.

Supplementary Figure 3

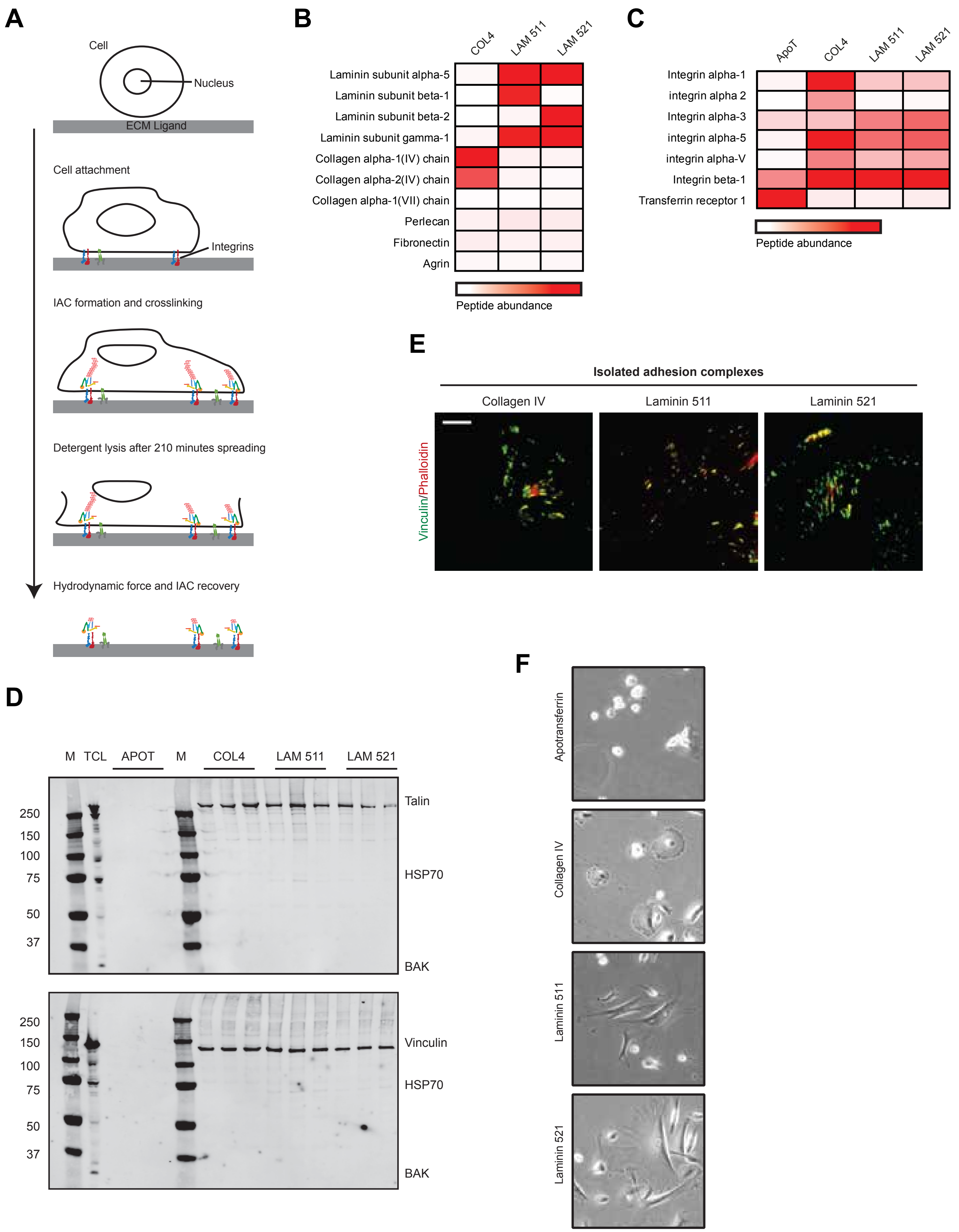

Supplementary Figure 3: (A) Schematic representation of adhesion complex isolation. (B) Heat map summary of peptide abundance of the top 10 most abundant matrix (defined by August 2014 matrisome55) proteins identified by MS in commercially available matrix-ligands. (C) Heat map summary of receptors identified in adhesion complexes by MS. (D,E) western blot and immunofluorescence of isolated adhesion complexes. Scale bar in (E) is 20 μm. (F) Phase contrast of cells after 210 minutes of spreading on either matrix-ligand or apotransferrin. LAM511, laminin-511; LAM521, laminin-521;COL4, collagen IV.

Supplementary Figure 4

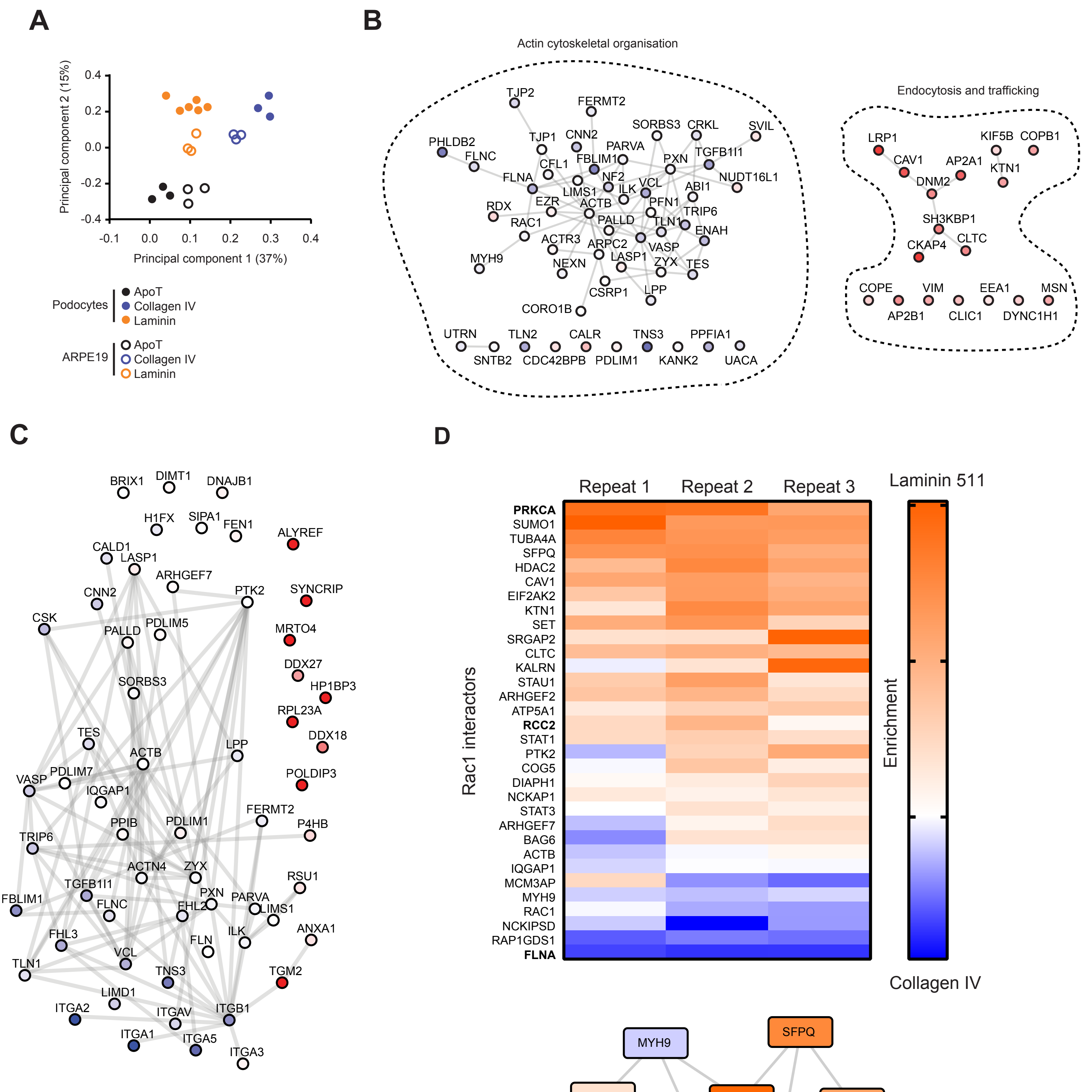

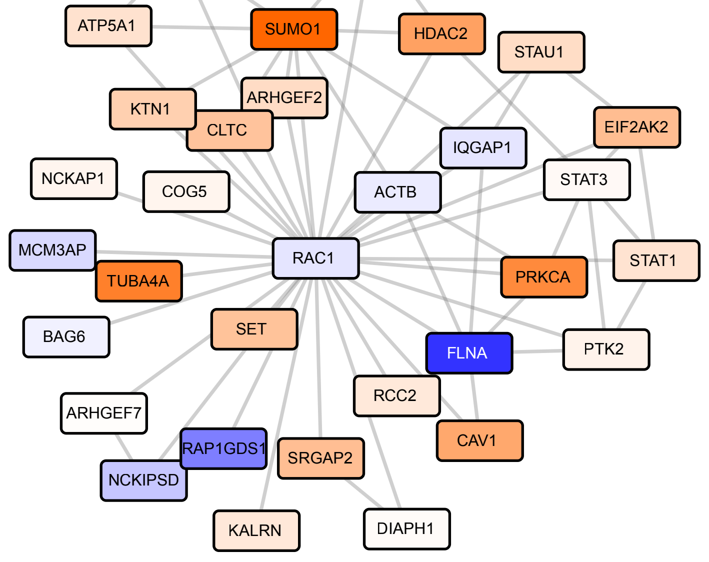

Supplementary Figure 4: (A) Principal component analysis of mass spectrometry data from podocytes and ARPE19 cells. Circles represent biological replicates. (B-C) Protein-protein interaction network constructed from known integrin adhesome components that were identified by mass spectrometry, sorted by Gene Ontology terms (B) or consensus adhesome components (C). Edges represent protein-protein interactions, nodes represent proteins. Coloured nodes represent those with significantly different abundance in laminin (511 and 521 combined) integrin adhesion complexes (IACs) compared with collagen IV IACs. (D) Analysis of Rac interacting proteins from MS datasets. Heat map and interaction network demonstrate the enrichment of proteins in either type IV collagen (blue) or laminin 511 (orange) adhesion complexes.

Supplementary Figure 5

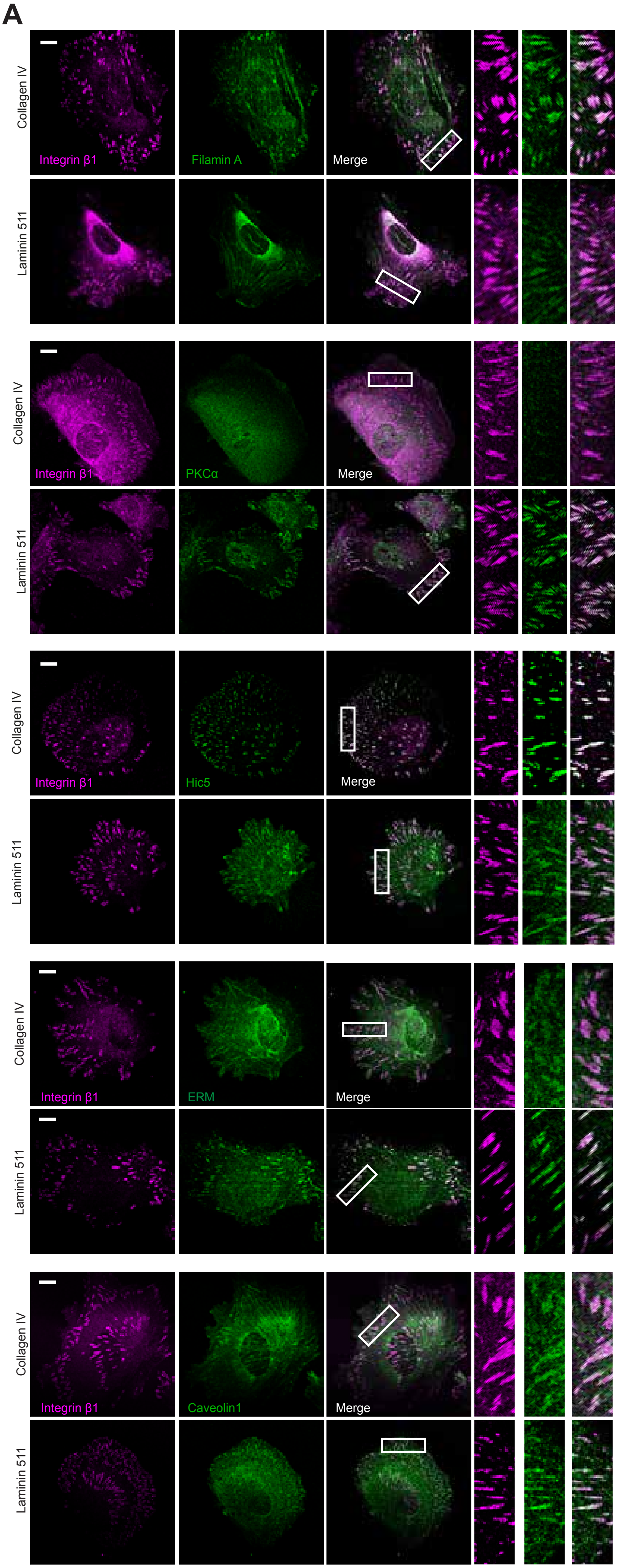

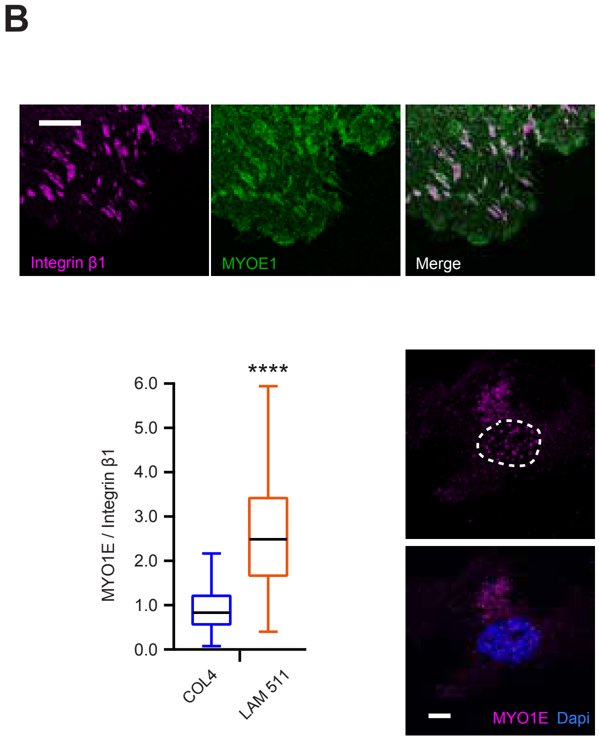

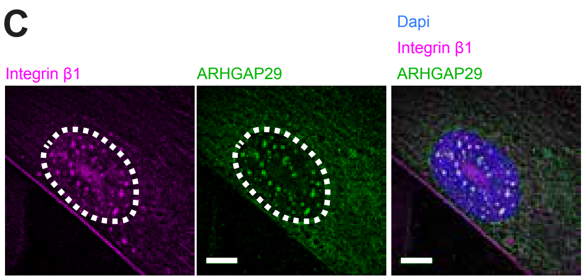

Supplementary Figure 5: (A-C) Immunofluorescence of integrin adhesion components. (A) Immunofluorescence of colocalization of integrin β1 with: Filamin A, PKCα, HIC5, ERM and Caveolin 1. (B) On laminin MYO1E localized to peripheral adhesions and circular adhesions underneath the nucleus. Ratio imaging box plots indicate 25th and 75th percentiles (lower and upper bounds, respectively), 1.5× interquartile range (whiskers) and median (black line). (C) ARHGAP29 localisation specifically to circular adhesions underneath the nucleus. Scale bar in (A) represents 10 μm, in top panel (B) 1μm, bottom panel 10 μm. Scale bar in (c) represents 2μm.

Supplementary Figure 6

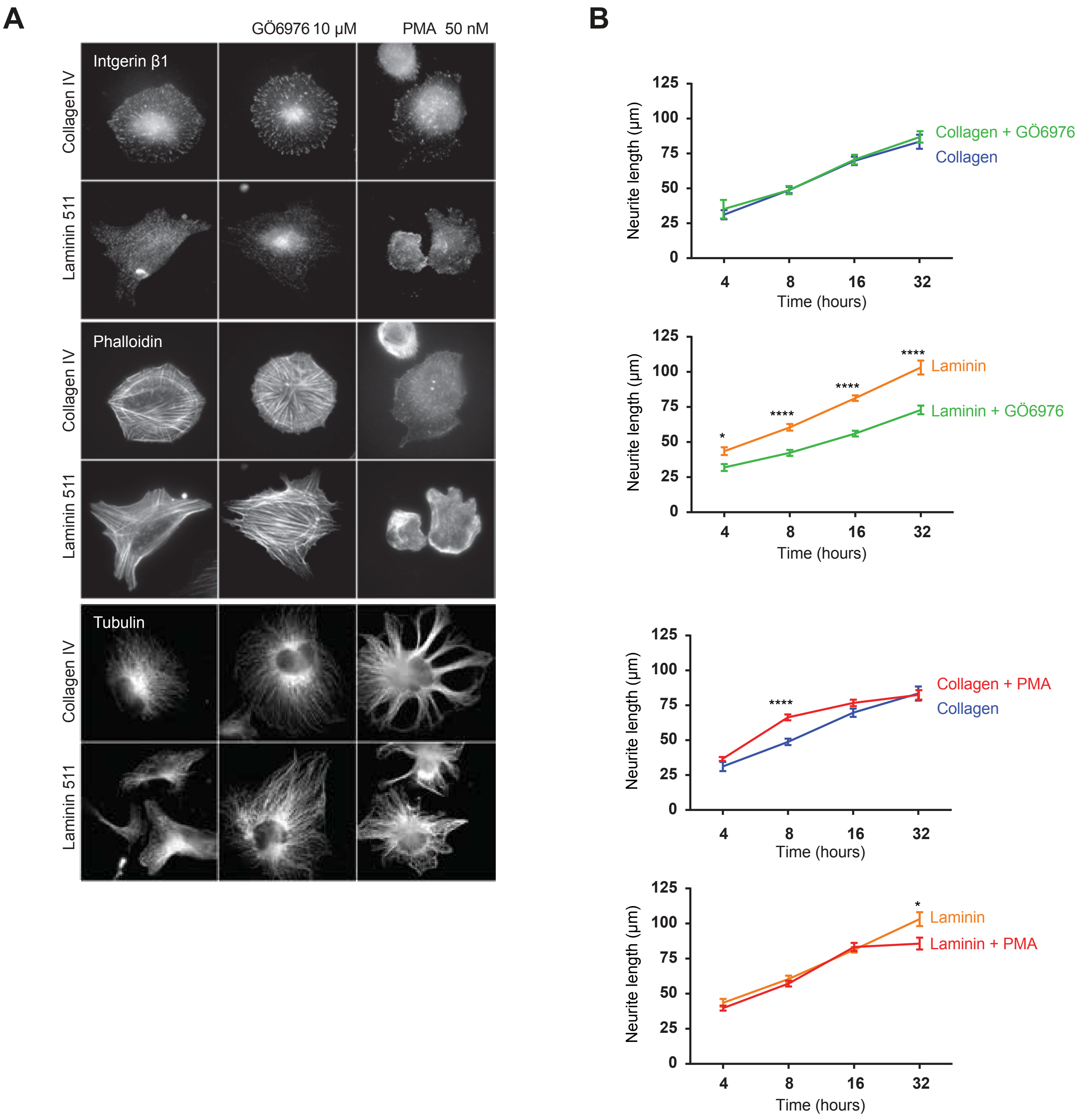

Supplementary Figure 6: (A) Immunofluorescence of podocyte actin and microtubule cytoskeleton response to PKC perturbation during cell spreading on matrix ligand. (B) Dorsal root ganglion neurite out growth was assessed on collagen IV or laminin under perturbation of PKC and length of outgrowth was compared using fluorescence imaging. ****, $p < 0.0001$
