# Supplementary material for: Basement membrane ligands initiate distinct signalling networks to direct cell shape
Source: Matrix Biol. 2020 Aug;90:61–78. doi: 10.1016/j.matbio.2020.02.005 (PMC7327512; doi:10.1016/j.matbio.2020.02.005)
Supplement: Multimedia component 2 [file mmc2.pdf]

Supplementary Table 1: Ligands and receptors identified by mass spectrometry. Peptide intensity is shown for each protein and there are 3 biological replicate samples for each of the four ligands used (APO- apotransferrin, COL4- collagen IV, LAM511- laminin 511 and LAM521- laminin 521).

**Ligands**																
Official Symbol	Uniprot	Peptide count	Peptides used for quantification	Confidence score	APO	APO	APO	COL4	COL4	COL4	LAM511	LAM511	LAM511	LAM521	LAM521	LAM521
COL4A1	P02462	16	10	579	716	2191	731	4834	4393	6919	2908	1609	1514	1060	1632	1152
COL4A2	P08572	26	18	667	2635	2682	2114	8537	8131	7637	4368	3530	3320	4208	4126	3947
LAMA5	O15230	65	42	2137	2844	369	1008	1155	922	1252	5332	5374	5506	5167	7531	4824
LAMB1	G3XAI2	25	17	632	303	73	68	130	189	144	2428	1905	1923	236	730	311
LAMB2	P55268	29	16	829	844	201	183	2500	2452	2580	1182	854	858	3653	3749	2742
LAMC1	P11047	36	27	1119	2485	1208	1845	1310	1333	1310	5612	4134	4346	4411	6036	4088
TF	P02787	46	30	2300	214310	266682	216682	521	542	1049	470	403	542	517	403	459
**Receptors**																
Official Symbol	Uniprot	Peptide count	Peptides used for quantification	Confidence score	APO	APO	APO	COL4	COL4	COL4	LAM511	LAM511	LAM511	LAM521	LAM521	LAM521
ITGA1	P56199	8	5	148	34	30	6	480	524	434	123	78	91	96	121	96
ITGA2	E7EMF1	7	3	108	5	5	1	171	160	219	16	9	18	12	23	13
ITGA3	P26006	17	10	378	74	65	75	130	76	118	232	211	226	250	291	260
ITGA5	P08648	9	4	235	30	15	31	856	813	1062	295	179	297	265	328	269
ITGAV	P06756	10	4	107	4	14	29	197	195	278	120	116	120	147	161	164
ITGB1	P05556	15	9	411	173	176	275	1944	2030	2659	764	582	803	895	964	908
TFRC	P02786	16	11	343	1631	3052	3042	39	37	38	38	21	25	35	38	35

Supplementary Table 2: Adhesome components identified in different cell lines by mass spectrometry. Peptide intensity is shown for each protein and there are 3 biological replicate samples for each of the four ligands used (APO- apotransferrin, COL4- collagen IV, LAM511- laminin 511 and LAM521- laminin 521).

		ARPE19 cells									Podocytes							
Official Symbol	Uniprot	Peptide count	Unique peptide	Conf. score	COL4	COL4	COL4	L511	L511	L511	Pep tide cou nt	Unique peptide	Conf. score	COL4	COL4	COL4	L511	L511	L511
ACTN4	O43707	73	21	5161	2892463	3095557	3050365	2557830	2469678	2616140	85	32	4531	31845	27101	32283	26053	26756	26940
ALYREF	Q86V81	12	6	360	138726	133772	114675	121258	105605	89521	8	7	244	411	303	325	1885	1931	1619
ANXA1	P04083	20	5	962	49302	55165	50532	64017	63266	63089	21	9	898	548	766	639	874	704	1017
ARHGEF7	B7Z6G2	5	4	85	3183	3415	4787	4300	3536	5407	10	6	152	303	253	172	234	300	286
BRIX1	Q8TDN6	6	2	95	14763	11602	14682	9882	10842	14026	11	9	258	237	340	353	277	432	308
CALD1	Q05682-3	25	10	752	295161	251532	189711	213847	171438	192990	41	4	1331	4268	3474	5767	3215	2692	3176
CNN2	B4DDF4	12	6	466	77228	74094	78234	67919	69818	66095	19	5	814	1595	1529	1871	780	884	939
CNN2	H3BQH0	12	6	466	77228	74094	78234	67919	69818	66095	14	3	361	101	104	128	278	351	263
DDX18	Q9NVP1	10	2	381	15501	13640	14177	15131	13348	13584	17	10	666	70	65	89	257	383	190
DDX27	Q96GQ7	10	4	222	12022	11393	10976	10740	9934	11028	20	15	502	112	75	64	219	297	186
DIMT1	Q9UNQ2	6	2	237	4581	5289	4897	6466	6769	6644	7	4	184	12	20	57	22	38	26
FERMT2	H0YJ34	13	8	280	21423	24139	26678	19853	23907	23755	13	7	351	340	410	311	237	212	295
FHL2	J3KNW4	10	8	350	36260	41179	33048	38621	25650	32940	18	11	559	3336	3647	4296	2503	2790	2256
FLNC	Q14315	127	77	5969	2566299	2619060	2498742	2457435	2212373	2543131	109	65	3990	15633	14171	12694	9775	8444	9402
H1FX	Q92522	4	3	119	28974	27746	26493	19691	16886	20548	4	3	64	19	34	22	12	16	11
HP1BP3	Q5SSJ5	18	10	478	103810	104679	110743	96770	91868	105379	19	12	607	174	77	119	1096	1231	784
ILK	Q13418	9	6	149	11160	13526	13182	16302	16687	13338	21	13	471	1531	1213	1511	1162	1040	1261
IQGAP1	P46940	107	66	5939	3257534	3433643	3484294	3325096	2934795	3337063	126	91	5424	58443	52010	49045	49209	51594	48037
ITGB1	P05556	7	4	221	34367	37771	46657	46857	38056	40750	15	9	411	1944	2030	2659	764	582	803
LASP1	Q14847	3	2	139	21848	21061	18466	19128	13056	14377	16	11	423	1651	1487	1775	1821	1767	2099
LPP	Q93052	4	2	81	4210	5378	2575	3666	2370	2984	16	16	226	1878	1563	1535	1273	995	1294
MRTO4	Q9UKD2	10	5	184	150169	109136	98829	89281	68079	83318	14	12	331	178	158	258	1223	1726	1164
P4HB	P07237	17	5	419	38900	37245	36225	33302	28272	23054	26	3	744	142	134	178	207	231	283
PALLD	Q8WX93	26	3	825	11555	13050	15191	12891	7335	14173	32	23	915	3429	3185	3851	3903	3945	4148

PDLIM1	O00151	9	8	283	114318	86551	88769	84113	58756	74418	18	12	465	4025	3166	3691	3825	3733	4384
PDLIM5	Q96HC4	18	14	626	118742	127849	106886	125389	95147	114955	19	15	506	1405	835	917	1126	1101	1225
PDLIM7	Q9NR12	13	8	445	73174	84784	75513	93133	83416	92344	19	17	558	7722	6508	7108	6321	6126	6931
POLDIP3	Q9BY77	6	5	238	29729	27658	27625	28392	29180	27854	14	3	333	10	9	7	160	197	124
PPIB	P23284	10	6	437	101093	109971	112188	92466	93962	87152	16	9	465	1493	1535	1885	1425	1633	1658
PTK2	J3QT16	5	2	47	14169	10417	14238	13687	23059	18166	18	3	289	155	80	37	116	154	130
PXN	F5GZ78	2	2	104	1978	1849	1799	3892	4670	2510	8	3	183	444	309	297	297	283	275
RSU1	Q15404	4	3	53	45473	43532	27324	27418	14031	11166	11	4	208	707	565	465	643	755	842
SYNCRIP	O60506	28	15	1432	426828	413700	426892	337794	281470	302930	35	2	1307	1	3	2	93	131	63
TES	H7BYK1	7	3	61	4257	6417	4637	8695	5468	8373	8	3	128	28	220	144	26	98	100
TGM2	B4DIT7	24	13	754	151273	158544	173095	216946	185012	203628	40	27	1260	1361	999	1158	12481	11385	13789
TLN1	Q9Y490	121	84	6490	1407104	1449259	1472505	1592539	1420759	1555057	170	119	7731	90616	76348	71483	55978	49593	55287
TNS3	E7ERH3	16	10	459	72977	76562	64043	34617	38939	35959	18	9	376	857	795	670	279	211	285
VASP	P50552	9	5	130	25375	25415	14361	15476	18790	13417	17	14	717	5592	5199	6649	2949	2774	3538
VCL	P18206	48	32	1820	375167	372640	376119	373810	277707	360511	69	40	2826	23267	20614	24173	10934	8196	9551
ZYX	B4DQX7	9	8	229	93369	76702	53282	80818	74449	65885	8	5	260	533	584	692	495	470	517

Supplementary Table 3: Consensus adhesome proteins identified by mass spectrometry in podocyte integrin adhesion complexes. Peptide intensity is shown for each protein and there are 3 biological replicate samples for each of the four ligands used (APO- apotransferrin, COL4- collagen IV, LAM511- laminin 511 and LAM521- laminin 521).

Official Symbol	Uniprot	Peptide count	Peptides used for quantification	Confidence score	APO	APO	APO	COL4	COL4	COL4	LAM511	LAM511	LAM511	LAM521	LAM521	LAM521
ACTN4	O43707	85	32	4531	12292	15641	17227	31845	27101	32283	26053	26756	26940	27613	25544	27138
ALYREF	Q86V81	8	7	244	679	733	725	411	303	325	1885	1931	1619	1825	1700	1926
ANXA1	P04083	21	9	898	330	198	502	548	766	639	874	704	1017	1026	1091	810
ARHGEF7	B7Z6G2	10	6	152	145	60	44	303	253	172	234	300	286	249	232	258
BRIX1	Q8TDN6	11	9	258	323	130	246	237	340	353	277	432	308	252	189	331
CALD1	Q05682-3	41	4	1331	871	218	245	4268	3474	5767	3215	2692	3176	3027	3200	3484
CNN2	B4DDF4	19	5	814	119	208	327	1595	1529	1871	780	884	939	952	966	1086
CNN2	H3BQH0	14	3	361	24	72	96	101	104	128	278	351	263	267	235	251
CSK	P41240	9	5	215	7	18	8	452	479	359	201	154	202	270	221	234
DDX18	Q9NVP1	17	10	666	348	140	230	70	65	89	257	383	190	178	154	196
DDX27	Q96GQ7	20	15	502	163	92	127	112	75	64	219	297	186	164	126	240
DIMT1	Q9UNQ2	7	4	184	117	132	129	12	20	57	22	38	26	37	35	39
DNAJB1	P25685	8	2	79	16	4	8	16	23	23	33	22	23	27	19	26
FBLIM1	Q8WUP2	10	7	228	55	91	71	2044	1853	2468	736	698	719	916	883	790
FEN1	P39748	12	5	314	91	109	288	193	150	473	355	457	411	213	208	333
FERMT2	H0YJ34	13	7	351	66	8	54	340	410	311	237	212	295	342	325	269
FHL2	J3KNW4	18	11	559	193	327	485	3336	3647	4296	2503	2790	2256	3359	2910	2732
FHL3	Q13643	3	3	94	38	4	8	164	157	170	67	64	57	87	79	77
FLNC	Q14315	109	65	3990	3389	3007	3047	15633	14171	12694	9775	8444	9402	9977	10563	10144
H1FX	Q92522	4	3	64	19	42	12	19	34	22	12	16	11	48	14	14
HP1BP3	Q5SSJ5	19	12	607	869	230	314	174	77	119	1096	1231	784	734	577	1023
ILK	Q13418	21	13	471	206	164	218	1531	1213	1511	1162	1040	1261	1344	1332	1295
IQGAP1	P46940	126	91	5424	28136	17317	18721	58443	52010	49045	49209	51594	48037	42999	44821	45189
ITGA5	P08648	9	4	235	30	15	31	856	813	1062	295	179	297	265	328	269
ITGAV	P06756	10	4	107	4	14	29	197	195	278	120	116	120	147	161	164

ITGB1	P05556	15	9	411	173	176	275	1944	2030	2659	764	582	803	895	964	908
LASP1	Q14847	16	11	423	1134	316	492	1651	1487	1775	1821	1767	2099	2436	1960	2516
LIMD1	C9JRJ5	4	2	110	1	4	0	72	38	61	26	28	31	44	26	40
LIMS1	P48059-3	9	5	239	301	861	810	251	228	228	198	207	248	296	206	211
LPP	Q93052	16	16	226	134	43	83	1878	1563	1535	1273	995	1294	1425	1444	1204
MRTO4	Q9UKD2	14	12	331	438	494	681	178	158	258	1223	1726	1164	1354	1161	1379
P4HB	P07237	26	3	744	495	169	826	142	134	178	207	231	283	249	198	263
PALLD	Q8WX93	32	23	915	2451	886	923	3429	3185	3851	3903	3945	4148	4093	3966	4039
PARVA	J3KNQ4	11	6	411	47	52	133	857	691	643	535	435	627	800	685	681
PDLIM1	O00151	18	12	465	2339	898	802	4025	3166	3691	3825	3733	4384	5245	4366	4976
PDLIM5	Q96HC4	19	15	506	5833	125	190	1405	835	917	1126	1101	1225	1169	1127	1155
PDLIM7	Q9NR12	19	17	558	1110	674	624	7722	6508	7108	6321	6126	6931	8031	6957	7719
POLDIP3	Q9BY77	14	3	333	29	17	18	10	9	7	160	197	124	136	85	189
PPIB	P23284	16	9	465	785	918	1316	1493	1535	1885	1425	1633	1658	2361	2103	1674
PTK2	J3QT16	18	3	289	64	18	45	155	80	37	116	154	130	94	137	106
PXN	F5GZ78	8	3	183	64	47	126	444	309	297	297	283	275	339	318	296
RPL23A	K7EJV9	12	3	247	57	102	168	18	23	22	85	98	126	118	93	105
RSU1	Q15404	11	4	208	91	115	151	707	565	465	643	755	842	744	1009	819
SIPA1	F6RY50	11	2	115	1	1	3	8	12	0	19	38	20	13	19	4
SORBS3	O60504	7	4	82	115	97	21	266	233	221	342	581	86	142	101	75
SYNCRIP	O60506	35	2	1307	24	17	7	1	3	2	93	131	63	66	42	86
TES	H7BYK1	8	3	128	8	24	10	28	220	144	26	98	100	112	108	80
TGFB1I1	O43294	5	5	125	13	8	5	303	229	290	123	109	113	139	129	99
TGM2	B4DIT7	40	27	1260	3200	1908	2506	1361	999	1158	12481	11385	13789	13628	15175	13126
TLN1	Q9Y490	170	119	7731	8913	11154	11430	90616	76348	71483	55978	49593	55287	58141	58931	54493
TNS3	E7ERH3	18	9	376	85	65	47	857	795	670	279	211	285	235	293	233
TRIP6	Q15654	11	7	197	193	97	97	878	754	609	456	386	392	461	354	393
VASP	P50552	17	14	717	255	389	471	5592	5199	6649	2949	2774	3538	3625	3413	3762
VCL	P18206	69	40	2826	6435	1959	2673	23267	20614	24173	10934	8196	9551	10992	11324	11107

ZYX	B4DQX7	8	5	260	209	140	136	533	584	692	495	470	517	678	609	640

Supplementary Table 4: Geiger adhesome proteins identified by mass spectrometry in podocyte integrin adhesion complexes. Peptide intensity is shown for each protein and there are 3 biological replicate samples for each of the four ligands used (APO- apotransferrin, COL4- collagen IV, LAM511- laminin 511 and LAM521- laminin 521).

Official Symbol	Uniprot	Peptide count	Peptides used for quantification	Confidence score	APO	APO	APO	COL4	COL4	COL4	LAM511	LAM511	LAM511	LAM521	LAM521	LAM521
ABI1	A6NFN2	5	5	95	32	130	394	400	166	445	264	225	386	243	209	418
ACTB	P60709	59	21	3146	117153	396404	315653	527512	590540	434895	414823	575547	495075	414417	506263	409800
ARHGEF2	Q92974	19	6	296	68	132	141	65	61	76	154	183	133	118	113	115
ARHGEF7	B7Z6G2	10	6	152	145	60	44	303	253	172	234	300	286	249	232	258
ARPC2	O15144	17	8	493	177	285	370	1826	1853	2165	1396	1560	1830	1972	1771	1982
CALR	P27797	6	3	163	36	111	175	38	41	20	35	47	132	38	55	85
CAPN1	P07384	16	10	424	137	185	340	383	437	531	732	650	771	633	687	687
CAPN2	P17655	12	7	219	296	216	424	357	295	381	604	559	668	490	608	498
CAV1	Q03135	13	10	457	1101	1378	2080	594	681	799	2169	2908	2437	2623	2397	2156
CFL1	E9PK25	12	4	415	1359	3246	3690	1690	1782	2525	1570	3208	1103	1229	2042	996
CORO1B	Q9BR76	18	12	480	2292	283	327	1524	1656	1943	1583	1995	1699	1917	1653	1745
CORO2A	Q92828	8	2	99	21	5	9	24	23	15	33	33	55	30	31	37
CRKL	P46109	3	3	27	45	100	100	557	127	153	181	241	186	236	182	172
CSK	P41240	9	5	215	7	18	8	452	479	359	201	154	202	270	221	234
CSRP1	P21291	12	9	506	1289	574	671	2962	2859	2713	2538	2937	2837	3151	2841	2825
DNM2	F5H4R9	22	6	487	117	73	78	75	77	79	221	222	233	201	191	186
ENAH	Q8N8S7-2	14	7	282	93	117	88	1692	1644	1915	853	726	841	1060	969	1013
EZR	P15311	48	18	1712	5239	3011	3622	2876	2259	3142	3058	4431	3281	2937	3144	3486
FBLIM1	Q8WUP2	10	7	228	55	91	71	2044	1853	2468	736	698	719	916	883	790
FERMT2	H0YJ34	13	7	351	66	8	54	340	410	311	237	212	295	342	325	269
FHL2	J3KNW4	18	11	559	193	327	485	3336	3647	4296	2503	2790	2256	3359	2910	2732
FLNA	P21333	165	98	7418	16964	33005	36745	137430	126907	136438	63601	54963	59340	69322	72300	71334
GIT1	Q9Y2X7-3	11	2	182	18	6	15	195	182	248	102	98	120	128	90	143
HRAS	P01112	5	4	99	40	63	75	168	235	206	149	203	164	186	190	130
HSPA2	P54652	23	2	997	15	17	20	19	9	5	43	52	30	30	26	33
HSPB1	P04792	5	2	175	59	26	86	55	67	59	106	81	152	152	147	138
ILK	Q13418	21	13	471	206	164	218	1531	1213	1511	1162	1040	1261	1344	1332	1295
ILKAP	Q9H0C8	10	2	195	8	5	8	3	3	7	27	28	26	26	10	32
ITGA1	P56199	8	5	148	34	30	6	480	524	434	123	78	91	96	121	96
ITGA2	E7EMF1	7	3	108	5	5	1	171	160	219	16	9	18	12	23	13
ITGA3	P26006	17	10	378	74	65	75	130	76	118	232	211	226	250	291	260
ITGA5	P08648	9	4	235	30	15	31	856	813	1062	295	179	297	265	328	269
ITGAV	P06756	10	4	107	4	14	29	197	195	278	120	116	120	147	161	164
ITGB1	P05556	15	9	411	173	176	275	1944	2030	2659	764	582	803	895	964	908
KTN1	Q86UP2	47	17	1375	527	203	251	228	74	91	334	416	351	304	189	280
LASP1	Q14847	16	11	423	1134	316	492	1651	1487	1775	1821	1767	2099	2436	1960	2516
LIMS1	P48059-3	9	5	239	301	861	810	251	228	228	198	207	248	296	206	211
LPP	Q93052	16	16	226	134	43	83	1878	1563	1535	1273	995	1294	1425	1444	1204
LRP1	Q07954	27	12	267	55	44	44	34	17	24	109	120	156	118	107	78
MACF1	E9PLY5	47	2	1108	22	53	53	98	79	62	95	140	112	139	119	139
MSN	P26038	68	33	2929	5451	4645	5351	3774	3764	4888	9243	12056	8710	8290	8412	8693
MYH9	P35579	251	154	13045	40231	65220	59768	320896	332203	305759	263969	256400	257718	249990	270972	238406

NEXN	Q0ZGT2	24	8	426	224	28	25	73	70	82	67	76	78	62	54	46
NF2	P35240-2	11	4	156	94	183	91	119	134	180	85	80	80	92	86	91
NUDT16L1	K7EIN2	4	3	73	9	31	30	33	29	70	49	63	60	65	58	81
PALLD	Q8WX93	32	23	915	2451	886	923	3429	3185	3851	3903	3945	4148	4093	3966	4039
PARVA	J3KNQ4	11	6	411	47	52	133	857	691	643	535	435	627	800	685	681
PDPK1	C9JWR9	4	2	31	3	8	1	2	3	1	2	7	7	2	3	6
PEAK1	Q9H792	15	8	178	119	40	125	275	168	173	295	297	234	276	192	238
PFN1	P07737	9	6	378	904	933	1479	247	255	374	267	386	318	279	249	233
PLEC	Q15149-4	385	2	17616	357	86	136	1130	1135	935	919	758	817	983	788	904
PLEC	Q15149	389	2	17555	9	20	23	12	14	27	31	34	32	44	41	39
PPFIA1	Q13136	30	14	704	162	264	239	3015	2498	2942	1397	1053	1276	1502	1527	1534
PRKACA	P17612	3	2	62	8	8	19	86	114	125	67	63	92	89	79	118
PRKCA	P17252	8	3	62	13	6	10	7	9	12	57	66	46	27	36	14
PTK2	J3QT16	18	3	289	64	18	45	155	80	37	116	154	130	94	137	106
PTPN1	B4DSN5	8	5	128	312	417	485	2251	2063	3118	1649	1769	1975	1877	1639	2320
PTPN12	Q05209	5	2	79	23	7	5	36	40	40	52	38	57	40	32	46
PXN	F5GZ78	8	3	183	64	47	126	444	309	297	297	283	275	339	318	296
RAC1	P63000	9	4	246	448	598	607	657	870	1124	640	615	747	1012	1020	635
RASA1	B4DTL2	8	3	75	16	11	13	28	23	22	33	35	44	38	30	30
RAVER1	E9PAU2	19	15	315	142	226	202	251	239	287	753	802	650	547	558	765
RDX	P35241	43	14	1487	344	623	610	672	573	836	968	1233	1089	982	1001	1281
ROCK1	Q13464	26	2	186	8	3	14	5	8	4	7	9	1	4	7	7
SDC4	P31431	4	3	72	10	7	30	156	132	117	145	114	148	126	155	185
SH3KBP1	Q96B97	6	4	86	81	39	24	54	29	62	147	191	157	122	140	83
SLC16A3	O15427	2	2	23	12	28	19	17	22	5	31	20	15	12	14	12
SORBS3	O60504	7	4	82	115	97	21	266	233	221	342	581	86	142	101	75
STAT3	K7ENL3	17	15	551	399	254	368	502	375	433	506	579	545	480	427	508
SVIL	O95425	79	3	2166	12	6	3	91	104	133	157	140	161	159	113	176
TES	H7BYK1	8	3	128	8	24	10	28	220	144	26	98	100	112	108	80
TGFB1I1	O43294	5	5	125	13	8	5	303	229	290	123	109	113	139	129	99
TLN1	Q9Y490	170	119	7731	8913	11154	11430	90616	76348	71483	55978	49593	55287	58141	58931	54493
TRIO	O75962	38	17	374	1334	378	498	680	474	514	582	636	673	523	526	562
TRIP6	Q15654	11	7	197	193	97	97	878	754	609	456	386	392	461	354	393
VASP	P50552	17	14	717	255	389	471	5592	5199	6649	2949	2774	3538	3625	3413	3762
VCL	P18206	69	40	2826	6435	1959	2673	23267	20614	24173	10934	8196	9551	10992	11324	11107
VIM	P08670	83	59	5306	360297	578949	636434	137829	123408	188736	312518	282733	343806	359354	332240	368291
ZYX	B4DQX7	8	5	260	209	140	136	533	584	692	495	470	517	678	609	640

Supplementary Table 5: All proteins identified by mass spectrometry in podocyte integrin adhesion complexes. Peptide intensity is shown for each protein and there are 3 biological replicate samples for each of the four ligands used (APO- apotransferrin, COL4- collagen IV, LAM511- laminin 511 and LAM521- laminin 521).																
Official Symbol	Uniprot	Peptide count	Peptides used for quantification	Confidence score	APO	APO	APO	COL4	COL4	COL4	LAM511	LAM511	LAM511	LAM521	LAM521	LAM521
AAAS	Q9NRG9	9	3	268	24	26	37	6	5	13	78	84	65	53	31	82
AARS	P49588	14	8	210	92	71	183	132	104	1953	87	88	483	108	66	107
AATF	Q9NY61	10	6	251	79	68	57	44	75	102	51	66	44	53	25	34
ABCD3	E7EUE1	15	8	314	1415	81	94	272	93	118	310	322	302	319	277	277
ABCE1	P61221	8	3	113	154	119	152	62	81	87	126	153	210	125	111	139
ABCF2	Q9UG63	9	3	74	4	10	0	142	177	119	76	64	69	93	78	68
ABCF3	Q9NUQ8	6	2	79	4	10	15	39	45	26	21	30	25	36	37	25
ABHD12	Q8N2K0	4	2	31	8	2	4	5	8	11	10	12	16	21	9	16
ABI1	A6NFN2	5	5	95	32	130	394	400	166	445	264	225	386	243	209	418
ABLIM1	Q5T6N4	14	7	190	82	17	37	280	294	271	198	177	212	197	173	189
ABLIM3	O94929-3	11	5	368	57	35	27	372	401	248	360	399	411	378	390	346
ABT1	Q9ULW3	4	3	41	130	123	61	34	24	51	28	45	34	31	20	41
ACAA1	H7C131	2	2	43	33	11	23	214	151	224	129	146	123	178	179	122
ACAA2	P42765	2	2	55	10	19	63	0	1	4	1	3	5	3	2	4
ACACA	Q13085	28	4	449	31	3	11	15	6	6	9	14	11	8	7	10
ACACB	F8W8T8	19	2	224	1	1	4	20	1	16	25	25	30	34	39	24
ACADVL	P49748-3	17	6	358	430	121	192	106	77	142	116	73	119	129	86	123
ACAT1	P24752	7	2	126	162	12	18	24	0	4212	710	1	219	3	2	2747
ACAT2	Q9BWD1	12	9	257	286	188	427	363	262	368	278	405	407	363	249	369
ACBD3	Q9H3P7	8	3	99	14	5	55	113	116	141	68	55	48	66	79	72
ACBD5	Q5T8D3	7	4	67	100	163	213	202	233	259	411	383	421	518	410	518
ACIN1	Q9UKV3-5	25	12	672	1069	227	242	72	7	41	174	164	102	89	59	100
ACLY	P53396	37	27	1127	1372	1342	1799	1466	1231	1264	1969	2201	2350	1966	1863	2199
ACO1	P21399	8	2	100	18	17	18	30	29	41	74	76	124	73	77	95
ACO2	A2A274	3	2	75	14	0	16	1	2	4	4	3	6	4	1	3
ACOT7	O00154-6	12	3	208	203	29	26	33	29	24	36	42	57	27	27	52
ACOT9	Q9Y305	7	3	122	31	22	26	10	4	14	12	18	24	14	11	10
ACOX1	Q15067	8	4	79	129	28	13	19	10	6	16	26	37	20	22	15
ACTB	P60709	59	21	3146	117153	396404	315653	527512	590540	434895	414823	575547	495075	414417	506263	409800
ACTBL2	Q562R1	19	2	938	3	15	8	85	59	84	49	59	61	66	49	68
ACTC1	P68032	38	6	1805	1325	974	975	1018	444	766	540	1874	1931	683	900	640
ACTL6A	O96019	10	6	343	277	79	210	89	103	126	421	465	354	439	294	488
ACTN3	Q08043	27	2	888	45	164	145	271	254	286	318	282	303	314	381	295
ACTN4	O43707	85	32	4531	12292	15641	17227	31845	27101	32283	26053	26756	26940	27613	25544	27138
ACTR1A	P61163	14	5	378	627	265	234	581	453	304	980	1240	1099	1059	944	985
ACTR2	P61160	25	6	836	145	362	512	866	903	1051	607	766	838	769	822	725
ACTR3	P61158	26	15	927	2005	2554	2776	6914	8033	6703	6571	8156	8156	7989	8878	8410
ADAMTS1	Q9UHI8	5	3	67	76	67	60	240	378	472	84	44	60	58	78	77
ADAMTS20	J3QT00	9	2	47	75	75	84	19	10	13	38	25	38	37	26	38
ADAMTS5	Q9UNA0	7	3	126	44	52	39	70	84	60	97	66	89	75	91	85
ADAR	E7ENU4	45	32	1387	1005	1049	1350	527	604	696	1916	2123	1548	1540	1171	2012
ADD1	A2A3N8	19	6	527	423	338	422	277	147	139	369	515	384	458	421	431

ADD3	Q9UEY8-2	18	13	468	529	953	919	850	806	704	1670	1473	1657	1685	1685	1616
ADH5	P11766	6	4	103	124	52	64	86	65	30	99	107	73	54	77	61
ADK	P55263	7	3	130	10736	30	56	1525	5	13	409	20	18	250	15	19
ADNP	Q9H2P0	20	14	444	175	59	55	31	13	32	119	202	91	77	55	87
ADRM1	Q16186	2	2	77	21	12	55	44	41	20	23	38	26	45	45	39
ADSL	E7ERF4	7	2	123	41	38	60	13	10	6	15	22	20	24	16	25
AFAP1	Q8N556	12	5	115	335	22	27	181	141	467	116	135	182	149	133	151
AFAP1L2	F5GZE1	12	5	287	70	18	7	147	72	50	41	30	37	32	25	26
AFG3L2	Q9Y4W6	8	3	138	8	42	16	233	49	174	242	59	94	101	68	201
AGPS	O00116	6	3	49	12	1	11	0	4	3	12	12	10	5	2	3
AGRN	O00468-6	38	4	1121	95	65	51	361	371	195	703	594	804	647	777	791
AHCTF1	Q8WYP5-2	38	17	741	767	549	614	2051	2188	2666	1186	1027	1228	1651	1615	1494
AHCY	P23526	16	10	488	8247	685	914	1113	475	506	560	479	584	608	480	674
AHCYL1	O43865	8	2	98	20	37	21	11	6	5	33	32	23	34	25	24
AHNAK	Q09666	402	299	13365	105732	22582	25998	127687	99315	82412	89518	76137	73392	80665	73518	76130
AHNAK2	Q8IVF2	47	13	360	749	2028	1852	409	376	590	825	891	759	889	965	916
AIFM1	O95831	9	2	134	6	1	4	2	2	0	4	11	10	5	8	4
AIM1	Q9Y4K1	8	3	71	272	6	28	71	61	36	60	32	43	42	30	27
AIMP1	Q12904	7	6	157	429	67	111	202	135	133	356	342	389	316	215	331
AIMP2	Q13155	5	3	181	1054	196	218	194	82	56	268	256	286	244	231	196
AIP	O00170	6	3	112	36	18	51	24	30	29	61	58	72	76	41	75
AJUBA	Q96IF1	7	2	56	22	9	6	21	22	34	34	23	28	20	30	35
AKAP12	Q02952	19	9	284	101	174	217	297	209	270	358	279	360	254	269	314
AKAP13	Q12802	18	7	157	577	489	270	1580	1387	1357	1013	1155	1008	1146	1228	1001
AKAP17A	Q02040	15	5	139	70	144	61	219	145	313	83	87	105	113	97	136
AKAP2	Q9Y2D5-4	28	13	800	794	566	635	1065	1100	1088	2023	1918	1796	1830	1812	1987
AKAP8	O43823	7	3	94	9	17	16	15	13	25	19	38	13	26	21	15
AKAP8L	Q9ULX6	6	3	102	236	810	828	301	272	300	678	778	1026	937	694	970
AKAP9	Q99996	61	19	744	1886	648	755	1005	648	770	1247	1426	1457	1066	1104	1191
AKR1B1	P15121	14	7	285	1307	1318	1716	429	420	636	744	881	874	895	791	812
AKR7A2	H3BLU7	4	2	71	53	60	49	7	10	20	7	9	14	12	9	12
ALB	P02768	17	10	499	12389	25262	6908	16332	7852	22349	2976	824	1352	1210	1228	1021
ALDH16A1	Q8IZ83	15	8	546	184	178	163	307	213	285	358	492	365	319	314	393
ALDH18A1	P54886	15	5	271	122	32	93	11	10	21	34	34	44	30	20	32
ALDH7A1	F8VS02	5	2	62	141	26	28	81	110	15	61	44	32	127	90	106
ALDOA	P04075	25	15	782	2961	4129	5538	1379	1412	1921	2093	2653	3144	3439	3010	3491
ALDOC	P09972	10	3	186	37	18	94	65	53	49	118	107	161	148	122	132
ALYREF	Q86V81	8	7	244	679	733	725	411	303	325	1885	1931	1619	1825	1700	1926
AMPD2	H0Y360	9	5	62	120	122	99	63	105	131	118	72	137	81	96	135
ANAPC7	Q9UJX3	8	2	116	8	3	55	126	87	100	97	107	104	85	77	90
ANK3	Q12955	34	8	156	108	407	339	1640	1449	93	1208	1051	1100	1179	1440	1254
ANKFY1	Q9P2R3-4	16	2	248	194	246	286	29	41	38	47	55	36	49	37	40
ANKHD1	Q8IWZ3	14	2	191	0	0	0	7	7	4	16	18	20	15	8	18
ANKRD12	Q6UB98	15	4	64	1430	2020	492	424	1028	772	1545	1844	1140	844	1835	1383
ANP32B	Q92688	8	3	250	183	229	236	32	23	71	64	98	52	54	46	69
ANP32E	Q9BTT0	6	3	200	64	46	68	9	15	34	26	39	21	18	9	24
ANXA1	P04083	21	9	898	330	198	502	548	766	639	874	704	1017	1026	1091	810

ANXA11	B4DVE7	11	4	182	125	76	163	73	128	124	133	115	152	158	131	147
ANXA2	P07355-2	44	11	2227	12096	17683	16776	22574	23058	21169	23538	27735	29766	29982	26671	22577
ANXA3	P12429	8	5	150	388	291	517	96	99	144	90	108	113	107	79	101
ANXA4	P09525	10	4	299	132	110	170	32	29	34	60	74	56	78	53	57
ANXA5	P08758	18	11	528	824	814	1077	227	222	388	362	555	355	352	288	334
ANXA6	P08133	25	13	623	1035	529	751	342	304	365	556	615	619	505	497	484
ANXA7	B4DT77	11	5	270	120	134	53	108	103	136	198	218	210	189	186	225
AP1B1	Q10567	34	6	1012	92	173	181	46	45	38	105	197	56	83	39	62
AP1G1	B3KXW5	13	10	407	276	142	152	105	63	84	181	197	204	143	104	135
AP1G2	O75843	6	3	78	39	6	13	9	7	7	27	28	20	15	5	7
AP1M1	Q9BXS5	9	4	236	142	52	139	43	36	85	55	143	102	73	48	64
AP2A1	O95782	42	17	1377	687	641	1161	659	592	778	2112	1933	2483	2767	2178	2103
AP2A2	O94973	32	12	1209	919	177	310	254	177	145	833	788	979	846	804	737
AP2B1	P63010	46	16	1837	1365	967	1245	525	401	860	1652	2311	1817	1328	1287	1009
AP2M1	Q96CW1	21	5	591	459	85	197	77	80	67	639	645	763	620	556	494
AP2S1	M0QYZ2	6	4	158	94	111	134	107	116	111	391	318	419	459	388	393
AP3B1	O00203	20	9	406	69	105	158	166	158	213	310	292	307	340	247	277
AP3D1	O14617	15	5	214	40	120	212	27	16	46	185	257	257	173	124	194
AP3M1	Q9Y2T2	3	3	78	21	2	15	7	12	14	44	53	67	55	38	53
APBB1IP	Q7Z5R6	7	2	51	3	52	55	23	31	41	36	36	52	49	39	42
APEH	C9JIF9	5	3	107	7	1	14	16	29	11	45	49	51	30	30	43
APEX1	P27695	12	10	215	211	493	502	432	290	470	1349	1858	1317	1425	1014	1219
APOBEC3B	B0QYD3	7	2	125	7	8	4	3	2	1	3	3	4	2	4	5
APOBEC3C	Q9NRW3	6	4	124	115	44	31	72	68	56	142	244	152	149	162	108
APOBEC3G	Q9HC16	6	2	56	58	32	51	66	43	69	75	93	93	91	68	67
APOL2	J3KQL8	5	3	104	21	30	54	14	13	14	16	29	26	27	30	17
APPL2	Q8NEU8	6	3	74	17	24	11	3	4	7	14	9	11	9	6	9
AQR	O60306	24	13	460	86	49	62	37	58	46	141	164	118	98	84	124
ARAP1	Q96P48	14	6	108	242	84	38	41	73	138	62	101	101	246	124	86
ARCN1	P48444	20	3	488	11	36	40	184	203	225	191	241	238	274	212	199
ARF4	P18085	9	3	306	132	31	25	68	64	33	105	103	122	98	119	69
ARF6	P62330	3	2	126	61	68	116	98	101	97	120	158	173	119	176	77
ARFGAP3	Q9NP61	6	2	96	8	15	5	8	0	1	18	23	15	11	13	7
ARFGEF2	Q9Y6D5	24	3	228	120	283	507	50	26	92	194	327	308	186	163	206
ARHGAP29	Q52LW3	21	9	375	49	43	32	109	92	97	133	153	157	161	130	160
ARHGEF1	M0QZR4	7	5	136	30	25	100	52	75	58	136	166	150	136	107	154
ARHGEF2	Q92974	19	6	296	68	132	141	65	61	76	154	183	133	118	113	115
ARHGEF28	Q8N1W1-3	13	4	84	21	63	19	42	50	53	35	70	73	84	100	86
ARHGEF40	Q8TER5-3	15	7	134	73	27	37	148	168	261	167	158	211	214	188	192
ARHGEF7	B7Z6G2	10	6	152	145	60	44	303	253	172	234	300	286	249	232	258
ARID1A	O14497	13	8	79	38	63	67	87	64	45	230	126	139	125	96	163
ARID1B	G3XAA0	11	4	97	5	3	17	1	1	4	35	31	35	50	35	71
ARMCX3	Q9UH62	5	2	81	39	92	42	18	20	13	28	33	37	35	25	26
ARPC1B	O15143	20	12	598	433	561	697	1841	1770	1895	1829	1899	2095	2177	1817	2178

ARPC2	O15144	17	8	493	177	285	370	1826	1853	2165	1396	1560	1830	1972	1771	1982
ARPC3	O15145	12	3	259	51	49	55	363	351	492	245	309	347	390	294	299
ARPC4	P59998	10	3	287	250	412	586	677	733	858	440	516	560	648	603	621
ASAP1	Q9ULH1	7	3	97	53	4	4	26	14	16	27	31	16	17	18	27
ASCC3	Q8N3C0	27	10	427	48	43	56	73	70	70	234	197	250	182	165	274
ASH2L	F5H8F7	9	2	165	24	0	0	0	1	0	5	16	4	2	0	3
ASPM	Q8IZT6	35	4	155	45	94	120	63	60	80	152	204	215	239	197	179
ASUN	Q9NVM9	15	6	134	183	518	374	2363	3067	3457	2017	1612	2238	2523	2487	2948
ATAD2	Q6PL18	10	6	120	27	13	68	12	21	31	27	46	40	45	23	32
ATF7IP	G3V1U0	3	2	26	7	0	3	6	1	2	7	8	0	3	3	0
ATG7	O95352	10	2	137	175	30	296	100	122	168	97	62	126	133	158	141
ATIC	P31939	12	2	103	35	12	21	22	13	5	28	39	44	26	43	25
ATM	Q13315	29	11	192	57	125	105	266	365	587	219	200	250	224	206	241
ATP1A1	F5H3A1	9	3	198	10	33	64	18	18	24	22	22	25	24	19	26
ATP2A2	P16615	12	3	319	9	17	61	175	141	182	111	69	99	110	102	101
ATP2C1	H0Y9V7	5	2	170	5	2	1	5	4	7	37	27	30	50	38	39
ATP5A1	P25705	30	18	946	624	770	1562	550	442	577	762	860	1311	937	834	1049
ATP5B	P06576	27	3	1226	190	115	266	191	156	145	329	375	556	389	355	452
ATP5C1	B4DL14	5	3	79	82	150	219	22	24	34	30	40	61	58	40	54
ATP5H	O75947	3	3	76	69	29	53	9	8	9	12	27	31	26	21	24
ATP6AP1	Q15904	5	4	55	30	316	324	168	212	379	148	146	159	150	174	205
ATP6AP2	B7Z1I9	6	2	74	9	17	25	75	20	131	42	21	115	27	24	38
ATP6V0A1	F5H569	12	4	153	40	1	5	49	31	15	64	72	14	58	49	14
ATP6V0D1	F5GYQ1	15	10	512	296	170	182	281	315	371	237	343	293	317	251	293
ATP6V1A	B7Z1R5	20	14	749	686	817	1400	631	483	810	1188	1280	1580	1319	1101	1448
ATP6V1B2	P21281	20	5	683	52	71	106	29	36	30	28	65	64	66	55	57
ATP6V1D	G3V2S6	2	2	78	13	2	22	32	18	17	36	35	40	38	27	32
ATP6V1E1	P36543	8	3	245	26	22	27	48	50	47	75	83	59	72	79	72
ATP6V1G1	O75348	3	3	105	5	18	28	17	13	13	16	24	28	24	23	15
ATP6V1H	G3V126	7	6	228	147	208	165	60	25	50	34	86	69	29	30	19
ATRX	P46100	25	10	382	91	73	53	162	111	107	121	126	112	145	143	139
ATXN10	Q9UBB4	12	8	295	283	309	516	166	165	106	196	141	199	237	192	272
ATXN2	H0YH87	12	6	210	74	121	105	461	280	90	371	316	335	297	312	311
ATXN2L	Q8WWM7	8	2	98	10	3	19	14	25	1	53	61	53	56	48	72
B2M	F5H6I0	3	2	52	136	979	222	44	44	1764	29	80	198	26	94	603
BAG2	O95816	7	4	53	268	87	209	90	80	110	104	106	129	122	104	99
BAG6	B0UX83	9	4	129	787	9	19	86	30	33	53	47	51	39	40	49
BAIAP2L1	Q9UHR4	12	3	112	28	73	58	35	62	79	103	127	111	92	84	111
BAX	Q07812-5	2	2	66	161	2	0	26	5	2	30	40	29	34	18	21
BAZ1A	Q9NRL2	18	9	302	22000	102	54	2585	41	48	543	83	79	312	56	62
BAZ1B	Q9UIG0	35	20	801	305	244	271	176	221	312	357	417	306	297	246	340
BAZ2A	F8VU39	11	3	170	215	4	8	17	12	17	10	8	12	10	12	13
BBX	Q8WY36	5	2	65	355	556	636	647	614	827	770	594	785	859	749	822
BCAP31	P51572	5	2	120	23	18	28	16	19	19	46	65	68	48	54	61
BCAS2	O75934	5	2	49	45	26	42	149	146	203	83	158	111	145	136	103
BCCIP	Q9P287	8	5	197	109	118	152	130	126	49	338	349	396	290	259	296
BCLAF1	Q9NYF8	16	7	383	374	64	119	114	43	40	235	282	191	141	119	198

BGN	A6NLG9	6	5	256	13194	884	274	3189	205	311	729	43	169	723	104	150
BICC1	Q9H694	7	3	80	20	22	24	95	135	12	107	70	72	75	78	82
BIRC6	Q9NR09	22	7	172	1642	731	730	6869	6145	7378	2753	3025	2784	3911	3109	3792
BLM	H0YNU5	10	2	86	7	6	7	3	1	2	54	9	8	4	3	8
BMP1	P13497	6	2	53	18	7	14	61	77	50	64	38	61	46	65	62
BMP2K	Q9NSY1	8	2	97	79	5	22	6	2	8	17	7	13	17	14	14
BMS1	Q14692	28	12	669	378	254	161	120	77	120	116	150	99	99	79	120
BNC1	F5GY04	6	2	86	9	3	6	1	1	0	13	23	12	8	7	12
BOD1L1	Q8NFC6	23	4	200	6	6	7	2	3	3	13	22	13	13	7	13
BOP1	Q14137	16	10	433	343	152	233	37	78	1927	217	306	392	164	111	689
BPIFB6	Q8NFQ5	5	2	32	13	19	15	60	46	82	76	70	93	92	74	115
BPTF	Q12830	19	2	137	19	7	16	7	4	10	12	11	14	14	10	11
BRCC3	P46736	5	2	164	1	9	2	3	5	1	17	20	17	12	12	18
BRD4	M0QZD9	8	3	77	170	13	174	50	10	54	50	75	104	71	43	74
BRE	F8W733	4	3	89	60	39	32	19	12	9	80	117	124	105	80	107
BRIX1	Q8TDN6	11	9	258	323	130	246	237	340	353	277	432	308	252	189	331
BROX	Q5VW32	7	2	68	1	5	16	0	2	2	9	15	8	4	9	7
BSG	I3L4S8	3	2	69	20	8	37	19	16	6	16	16	24	18	18	15
BST1	A6NC48	8	2	84	31	41	31	109	123	130	51	73	93	57	62	92
BST2	Q10589	3	2	52	79	1	10	86	89	2	4	98	73	99	95	72
BTAF1	O14981	4	3	23	3	12	25	18	9	11	53	50	68	52	43	79
BTF3	P20290	2	2	26	39	57	36	14	59	22	28	116	13	13	66	11
BUB3	O43684	12	6	333	278	405	688	231	244	338	508	506	423	451	391	539
BUD31	C9JNV2	7	4	164	13	45	26	14	25	20	43	57	51	74	52	58
BYSL	Q13895	13	6	380	172	30	63	33	32	56	76	119	79	87	53	109
BZW1	Q7L1Q6	10	3	123	33	52	84	70	62	64	71	61	67	69	58	70
C10orf68	Q9H943	10	2	78	26	0	1	90	0	1	3	2	1	4	0	2
C14orf166	Q9Y224	11	4	385	108	290	208	91	84	122	115	133	119	119	126	114
C17orf85	Q53F19	7	4	179	17	13	15	4	6	9	30	52	30	27	16	16
C1QBP	Q07021	5	3	81	40	44	5	21	18	12	37	64	26	53	41	27
C22orf28	Q9Y3I0	16	9	475	574	1231	1221	607	586	749	873	869	922	858	948	1052
C2orf4	Q5R2V8	4	2	66	1	5	0	0	1	0	0	1	0	1	1	1
C3	P01024	12	4	63	49	48	60	59	71	78	99	97	133	153	142	132
C3orf17	Q6NW34	7	4	149	80	106	49	108	169	197	156	192	156	214	183	176
C5orf34	Q96MH7	4	2	38	10	17	13	6	5	93	10	8	13	11	8	15
C9	P02748	4	2	78	263	9	14	44	4	5	17	16	12	14	9	11
C9orf114	Q5T280	3	2	75	107	15	11	13	3	7	35	43	24	17	9	17
CAB39	Q9Y376	9	4	53	6	1	4	21	13	18	38	41	37	46	34	36
CACNA1I	A8MPS3	8	3	43	12	34	37	48	50	62	65	53	68	79	74	83
CACYBP	Q5R370	8	5	143	117	117	131	117	80	76	156	192	229	184	162	218
CAD	F8VPD4	33	18	657	279	134	203	289	241	198	330	357	418	256	241	353
CALD1	Q05682-3	41	4	1331	871	218	245	4268	3474	5767	3215	2692	3176	3027	3200	3484
CALM2	H0Y7A7	5	4	209	302	198	370	534	509	544	621	611	636	728	536	652
CALR	P27797	6	3	163	36	111	175	38	41	20	35	47	132	38	55	85
CAMK2B	H7BZC6	8	3	104	821	935	622	6019	5824	10329	3356	2961	3982	3864	3488	5086
CAMK2D	D6R938	10	4	209	69	95	89	86	87	46	102	163	174	155	172	166
CAMK2G	Q5SWX3	7	2	237	4	44	23	33	40	74	33	38	46	33	28	52

CAMTA1	H0YJY7	4	2	31	21	6	9	47	42	53	43	57	62	51	57	70
CAND1	Q86VP6	22	11	598	148	316	416	192	169	252	325	326	333	278	251	316
CANX	B4DGP8	9	5	119	260	106	147	40	27	51	77	71	104	63	62	61
CAP1	Q01518	26	17	882	5474	1454	1816	1732	1319	1273	2281	2115	1960	2242	2030	2033
CAPG	B4DU58	4	3	198	114	87	178	72	58	70	125	129	139	135	124	153
CAPN1	P07384	16	10	424	137	185	340	383	437	531	732	650	771	633	687	687
CAPN2	P17655	12	7	219	296	216	424	357	295	381	604	559	668	490	608	498
CAPNS1	P04632	8	5	245	346	511	652	472	566	588	663	650	911	983	1096	910
CAPRIN1	Q14444	12	5	301	348	509	592	520	576	429	689	649	642	752	730	896
CAPZA1	P52907	18	12	455	858	1482	2130	4479	4340	3499	3884	4153	4362	4647	4412	4631
CAPZA2	P47755	15	7	340	186	248	418	1023	1100	785	1176	1235	1306	1073	1133	1163
CAPZB	B1AK87	19	15	742	971	1904	1697	3650	3233	3745	2799	2890	3202	3338	3007	3394
CARD10	Q9BWT7	12	3	78	400	26	10	173	215	218	122	174	160	181	170	142
CARM1	Q86X55	7	2	120	10	0	0	5	3	6	3	4	5	4	4	7
CARS	P49589-3	8	2	80	714	1200	1239	244	239	267	575	420	705	656	825	789
CASK	O14936	23	16	333	211	208	209	679	690	677	1155	1042	1274	1208	1280	1258
CAT	P04040	7	4	115	287	82	104	75	71	52	82	56	70	55	44	43
CAV1	Q03135	13	10	457	1101	1378	2080	594	681	799	2169	2908	2437	2623	2397	2156
CAV2	P51636	4	4	189	290	364	440	380	335	199	686	882	880	953	691	752
CBFB	Q13951	4	2	134	11	6	9	8	18	14	26	31	13	22	9	31
CBX1	J3KS05	8	3	288	37	40	33	7	17	9	44	65	33	28	21	36
CBX3	Q13185	8	3	440	70	114	256	73	98	112	261	373	255	332	200	284
CBX5	G3V1X9	7	5	311	51	80	127	64	99	64	93	113	70	89	81	94
CBX8	Q9HC52	7	3	49	55	5	9	6	11	4	2	2	4	2	6	8
CCAR1	Q8IX12	18	3	312	94	24	11	45	39	29	51	49	36	37	40	50
CCDC132	B4DS55	9	3	57	4	3	2	3	6	10	15	25	18	19	12	19
CCDC137	I3L0U5	6	2	99	28	5	1	3	3	1	3	14	6	5	0	3
CCDC144A	A2RUR9	13	3	91	211	71	58	42	62	85	34	38	50	57	77	93
CCDC30	B4DXQ2	5	3	53	2	288	605	117	3	379	19	23	287	45	14	293
CCDC63	B4DY03	6	2	65	110	19	4	127	13	26	51	27	24	39	18	16
CCNH	D6RG18	3	2	25	8	51	3	214	321	126	97	79	55	103	203	64
CCNK	G3V2Q3	3	3	27	121	20	27	21	4	17	30	43	32	19	18	23
CCT2	P78371	34	22	1658	2090	1602	2739	1794	828	1219	2313	2519	2326	2065	2488	2160
CCT3	P49368	31	17	1098	2561	1359	2106	1780	1435	1575	3385	3173	3769	3570	3418	3709
CCT4	P50991	29	19	1064	529	729	1547	894	825	893	2131	2050	2242	2033	2306	2241
CCT5	E9PCA1	31	2	770	102	31	49	155	220	223	114	157	58	52	71	47
CCT6A	P40227	21	8	659	534	785	1368	480	508	528	1009	1025	1190	1015	1039	1158
CCT8	P50990	29	11	1122	2431	1463	1847	1198	1032	1089	1857	1474	1948	1892	1989	2121
CD2AP	Q9Y5K6	21	13	331	97	323	267	472	458	607	632	581	615	587	567	693
CD59	E9PNW4	3	2	169	140	233	309	45	62	63	54	63	71	74	76	71
CD63	F8VV56	3	2	44	137	100	122	11	5	26	4	8	5	5	3	2
CDC16	Q13042	6	2	45	3	4	4	4	8	12	10	11	14	9	12	11
CDC23	Q9UJX2	12	8	186	892	1273	1286	551	596	818	639	665	659	660	634	616
CDC27	G3V1C4	8	3	68	87	10	12	37	35	34	50	54	71	56	48	68
CDC40	O60508	9	6	133	200	57	40	46	57	68	73	80	72	79	61	80
CDC42BPA	F5H5N0	21	3	159	4	7	8	57	61	104	50	42	46	83	36	99
CDC42BPB	H0YLR5	5	2	46	948	0	3	76	3	4	6	3	3	5	4	5

CDC42BPB	Q9Y5S2	36	13	415	291	104	151	231	237	225	234	352	364	261	299	463
CDC5L	Q99459	32	23	935	1398	565	758	312	255	310	638	798	531	589	507	645
CDC73	Q6P1J9	21	10	328	79	128	182	183	264	398	312	346	220	204	150	191
CDH2	A8MWK3	4	4	96	100	70	48	501	526	514	405	358	425	401	415	477
CDK1	E5RIU6	5	2	185	112	183	174	109	107	150	56	59	51	37	26	56
CDK11B	J3QR44	18	4	300	19	30	44	15	18	19	44	41	23	21	18	27
CDK2	E7ESI2	6	2	147	22	35	39	9	11	16	11	10	6	9	16	7
CDK5RAP2	Q96SN8	19	2	145	175	136	185	90	162	42	494	82	372	282	231	439
CDK7	P50613	6	2	53	0	0	8	22	11	7	39	47	42	29	24	31
CDK9	P50750	9	3	108	25	13	22	4	5	6	14	40	20	8	14	20
CDKN1A	P38936	3	3	25	112	196	154	91	174	166	101	25	51	36	31	42
CDKN2A	J3QRG6	3	2	32	18	42	43	26	17	27	12	12	14	15	16	13
CDKN2A	Q8N726	4	2	96	2	3	1	0	0	0	3	4	1	3	1	3
CDKN2AIP	Q9NXV6	8	4	244	46	89	107	770	581	1004	898	1141	1080	926	844	1031
CDY2A	C9JMH9	4	2	44	157	8	8	14	13	12	23	23	17	19	19	24
CDYL	Q9Y232-2	9	4	164	222	30	16	61	520	56	77	195	51	50	226	55
CEBPZ	Q03701	27	18	622	149	203	150	47	65	96	151	212	120	117	102	157
CELF1	G5EA30	7	5	188	403	40	100	103	50	53	203	308	174	194	138	170
CENPB	P07199	8	6	248	50	84	24	13	13	13	51	55	43	33	19	52
CENPE	Q02224	33	2	220	0	11	20	2	2	18	9	7	9	10	12	11
CENPV	Q7Z7K6	2	2	48	3	4	6	0	0	3	2	3	2	0	0	0
CEP170	Q5SW79-2	22	10	239	3688	63	335	377	63	94	292	294	227	263	156	194
CEP170B	J3KQR7	16	5	84	210	844	392	778	650	727	312	334	452	436	382	512
CEP97	Q8IW35	6	2	50	4	21	14	50	72	97	51	45	57	58	67	58
CFL1	E9PK25	12	4	415	1359	3246	3690	1690	1782	2525	1570	3208	1103	1229	2042	996
CFL2	Q9Y281	10	3	244	22	16	33	95	104	7	93	150	106	105	119	59
CGNL1	Q0VF96	24	8	282	32	18	20	132	119	112	175	159	194	201	160	239
CHAD	O15335	8	6	397	48324	1786	83	6107	647	337	2687	318	281	2831	477	299
CHAF1A	Q13111	4	2	35	521	675	290	239	307	298	203	60	111	94	72	114
CHAMP1	Q96JM3	10	4	104	477	42	13	64	22	43	63	56	50	34	27	45
CHCHD3	C9JRZ6	10	6	144	1418	64	70	277	147	160	252	188	247	258	239	269
CHD1	O14646	24	3	242	437	16	11	85	23	12	38	26	23	36	21	21
CHD2	O14647	27	6	306	133	19	20	199	226	263	98	103	138	124	117	125
CHD3	E9PG89	26	4	471	5	13	8	37	41	60	24	23	24	35	27	24
CHD4	F5GWX5	56	20	1273	715	211	292	150	77	113	438	548	396	229	173	237
CHD7	Q9P2D1	20	3	163	111	229	140	827	798	1116	530	459	498	491	514	724
CHERP	J3QK89	16	7	325	470	98	120	109	64	105	245	251	199	169	169	259
CHN1	P15882	5	2	57	104	787	420	1471	1065	2492	736	604	893	851	1032	1455
CHST14	Q8NCH0	3	2	42	2	0	6	32	33	27	33	32	38	31	37	36
CHTOP	Q9Y3Y2	16	6	334	23	22	25	9	12	14	175	312	184	220	128	229
CIRBP	Q14011	6	5	123	76	118	139	747	314	410	610	930	756	945	658	1002
CIRH1A	Q969X6	12	6	274	628	263	538	206	146	217	300	335	280	259	248	254
CKAP2	Q8WWK9	7	3	80	83	11	9	19	81	83	33	62	73	106	81	92
CKAP4	Q07065	40	23	1503	922	1136	1640	768	665	895	2954	3437	3605	3603	2881	3587
CKAP5	Q14008	26	9	301	245	50	66	429	318	388	368	317	244	399	355	376
CKB	P12277	2	2	107	15	0	8	21	7	5	14	9	5	18	13	16
CLASP1	H0Y5T1	10	2	163	12	16	7	1011	935	412	176	342	153	487	438	196

CLASP1	B7ZLX3	17	5	339	135	138	162	84	74	45	118	110	108	108	102	145
CLASP2	F5H604	16	6	318	2150	2342	1101	327	261	352	264	190	208	222	185	241
CLIC1	O00299	17	12	707	1924	3300	3848	5353	4796	5008	8153	9883	9895	10993	10512	9290
CLIC4	Q9Y696	14	9	379	474	281	459	820	712	799	850	1029	976	1164	1041	884
CLINT1	Q14677-2	3	2	58	14	0	10	5	4	2	20	22	44	14	23	21
CLIP1	P30622-2	29	2	232	22	1	3	233	165	151	127	182	164	219	133	237
CLTC	Q00610	125	72	5614	8503	14308	25959	9911	8342	11401	26127	26833	31638	27794	23852	25812
CMAS	Q8NFW8	14	9	337	245	127	215	172	156	108	728	891	683	555	552	610
CMPK1	Q5T0D2	2	2	50	38	164	150	15	25	31	476	664	279	350	212	347
CNDP2	Q96KP4	13	8	181	125	209	175	234	227	143	458	500	478	462	431	572
CNN2	B4DDF4	19	5	814	119	208	327	1595	1529	1871	780	884	939	952	966	1086
CNN2	H3BQH0	14	3	361	24	72	96	101	104	128	278	351	263	267	235	251
CNN3	Q15417	25	2	949	525	191	71	919	313	42	561	798	292	326	484	182
CNOT1	A5YKK6	53	15	1262	111	188	206	836	666	922	1026	1068	1162	1030	880	1148
CNOT10	Q9H9A5-6	9	4	189	12	10	9	16	29	19	49	32	26	33	25	56
CNOT2	Q9NZN8	4	2	51	69	160	113	65	54	76	94	93	109	97	92	120
CNOT3	O75175	7	4	86	133	121	163	932	915	1336	578	546	564	659	624	709
CNOT7	Q9UIV1	3	3	68	42	17	32	145	148	130	169	160	200	200	185	245
CNP	P09543	5	3	76	245	10	19	69	33	52	127	183	179	122	151	56
COBLL1	Q53SF7	12	4	179	95	241	84	23	24	40	128	135	118	121	108	105
COG1	E9PBL8	18	6	144	94	171	95	44	40	80	56	47	53	51	53	56
COG3	Q96JB2	9	5	118	40	7	9	18	13	4	32	43	27	43	28	50
COG4	J3KNI1	15	4	120	4	9	34	20	16	43	50	102	133	117	71	133
COG5	Q9UP83	7	3	91	48	7	12	38	17	32	37	41	42	42	32	32
COG6	Q9Y2V7	5	3	73	145	7	9	19	9	8	14	19	13	13	11	14
COIL	P38432	9	3	141	24	19	48	9	13	12	57	75	43	45	34	53
COL11A1	P12107	5	3	76	3117	196	694	630	51	66	164	72	23	141	81	15
COL12A1	D6RGG3	19	4	166	3009	454	34	257	232	176	505	174	115	516	117	122
COL18A1	P39060	15	10	363	184	104	149	1043	1140	1273	642	546	693	859	1007	1014
COL1A1	P02452	16	9	245	15995	118	43	1666	49	88	313	51	47	149	42	38
COL1A2	P08123	11	5	208	854	22	59	60	10	21	17	18	24	19	14	23
COL4A1	P02462	16	10	579	716	2191	731	4834	4393	6919	2908	1609	1514	1060	1632	1152
COL4A2	P08572	26	18	667	2635	2682	2114	8537	8131	7637	4368	3530	3320	4208	4126	3947
COL4A3	Q01955	13	3	72	77	151	208	25	32	32	91	113	96	96	79	94
COL7A1	Q02388	35	16	628	218	109	129	584	587	568	1342	1075	1323	1559	1431	1301
COL8A1	P27658	6	3	79	21	58	99	69	96	42	251	205	267	233	241	195
COLGALT1	Q8NBJ5	8	3	94	178	68	154	60	55	60	102	84	112	107	89	94
COMMD3	H0Y4E5	7	5	106	14	10	19	18	14	34	31	34	31	29	37	43
COMT	E7EMS6	2	2	42	30	134	126	21	32	76	29	26	38	30	27	37
COPA	P53621	44	34	1381	1211	756	1580	649	637	817	1572	1473	1830	1462	1401	1514
COPB1	P53618	25	15	754	388	294	412	260	275	249	623	618	803	491	562	589
COPB2	B4DZI8	28	15	629	517	291	448	307	294	282	627	597	760	528	602	589
COPE	O14579	17	12	385	525	635	529	490	469	562	732	713	1015	924	917	845
COPG1	Q9Y678	25	10	868	7723	135	189	1029	115	186	662	356	502	492	415	421
COPG2	Q9UBF2	14	3	413	51	4	13	35	26	8	25	23	41	15	19	28
COPS2	P61201	9	7	141	139	36	31	134	133	101	135	142	151	117	107	180
COPS3	H7C3P9	3	3	108	151	35	48	59	17	13	120	134	163	78	124	68

COPS5	Q92905	10	6	233	83	33	58	68	48	79	93	135	153	109	131	125
COPS6	E7EM64	3	2	63	19	12	15	49	46	67	56	55	70	73	62	72
COPS7B	J3KQ34	4	3	36	92	21	70	181	27	15	444	115	95	529	86	134
COPS8	H7C3S9	2	2	70	85	3	33	44	21	40	44	48	58	62	58	62
COPZ1	F8VVA7	4	4	164	105	292	346	189	274	125	639	867	604	406	505	246
CORO1B	Q9BR76	18	12	480	2292	283	327	1524	1656	1943	1583	1995	1699	1917	1653	1745
CORO1C	A7MAP0	27	17	1041	2930	6013	6920	13851	13490	14936	10850	11758	10643	10225	11019	9747
CORO2A	Q92828	8	2	99	21	5	9	24	23	15	33	33	55	30	31	37
CORO2B	Q9UQ03	9	5	179	151	34	56	125	100	155	117	335	222	115	170	224
COX4I1	P13073	6	3	45	179	134	239	20	11	21	15	10	13	13	12	15
CPNE1	B0QZ18	5	3	126	36	61	85	43	76	78	155	113	170	140	146	157
CPNE3	O75131	10	8	140	52	82	98	365	365	492	316	318	321	352	302	258
CPSF1	Q10570	21	10	427	295	45	87	86	88	58	194	168	149	142	117	172
CPSF2	Q9P2I0	11	8	252	119	87	109	33	48	29	209	251	164	189	116	215
CPSF3	Q9UKF6	14	12	211	82	47	70	34	34	56	114	151	115	96	96	133
CPSF3L	Q5TA45	6	3	62	142	12	10	49	26	26	54	54	61	55	55	63
CPSF4	C9JEV9	5	3	84	4	2	1	1	0	1	5	17	6	8	5	9
CPSF6	F8WJN3	8	7	415	375	625	408	153	132	166	493	505	393	461	415	530
CPSF7	Q8N684-3	11	7	293	77	95	179	83	78	78	300	334	252	241	221	329
CRIP2	H0YFA4	9	3	90	244	141	115	149	113	50	401	440	355	506	427	361
CRKL	P46109	3	3	27	45	100	100	557	127	153	181	241	186	236	182	172
CRNKL1	Q5JY65	14	5	132	237	144	114	28	24	36	64	63	50	50	28	96
CROCC	B1AKD8	33	8	299	41	66	36	63	67	41	81	80	102	97	110	98
CRTAP	C9JP16	5	3	95	47	32	46	22	18	22	29	31	37	41	29	41
CS	O75390	9	4	116	212	310	352	76	74	108	67	49	73	65	57	70
CSDA	P16989	9	4	299	25	111	118	124	110	109	428	306	398	416	353	448
CSDE1	G5E9Q2	13	7	230	251	430	429	1168	1593	1518	928	965	1034	1073	1126	919
CSE1L	P55060	38	24	1339	2997	1098	1353	908	489	577	822	931	789	671	584	795
CSK	P41240	9	5	215	7	18	8	452	479	359	201	154	202	270	221	234
CSNK1A1	Q71TU5	6	5	125	15	58	100	313	345	409	233	205	256	304	265	245
CSNK2A1	E7EU96	11	4	361	93	104	187	260	257	395	427	465	535	436	409	623
CSNK2A1	P68400-2	7	2	138	34	80	13	533	586	576	413	471	388	454	436	355
CSNK2A2	P19784	10	8	213	422	103	157	113	72	216	285	341	310	188	199	236
CSNK2B	Q5SRQ6	7	4	204	26	33	84	48	60	64	108	129	110	143	126	126
CSRP1	P21291	12	9	506	1289	574	671	2962	2859	2713	2538	2937	2837	3151	2841	2825
CSRP2	F8VW96	9	8	314	129	102	93	1100	1040	1054	752	1078	1275	1501	1477	1303
CSTB	P04080	3	2	70	63	102	146	117	84	121	115	108	136	136	107	159
CSTF1	Q05048	10	9	317	176	146	121	63	64	59	180	298	249	183	161	212
CSTF2	P33240	13	4	242	27	28	23	3	3	15	13	21	26	11	9	33
CSTF3	Q12996	16	10	302	143	63	307	110	74	180	206	214	197	181	135	208
CTBP1	D6RAX2	6	3	101	7	20	31	6	5	3	22	39	40	25	19	18
CTBP2	P56545-2	14	6	169	244	125	114	71	30	47	85	111	80	81	64	95
CTCF	P49711	4	2	40	16	14	17	18	2	24	2	8	21	6	14	57
CTNNA1	P35221	37	15	1147	630	381	308	1703	1130	866	1235	1426	1305	1047	1172	1152
CTNNB1	B4DGU4	20	10	803	147	228	249	843	877	907	530	480	566	543	538	567
CTNNBL1	B4DE16	10	4	223	149	25	41	43	26	45	62	85	59	49	49	64
CTNND1	O60716-11	24	17	709	881	752	863	778	590	809	838	731	825	732	686	719

CTPS1	P17812	8	5	192	114	40	80	88	63	55	75	63	67	55	50	63
CTR9	Q6PD62	12	6	130	146	50	68	28	27	30	37	46	24	22	31	35
CTTNBP2NL	Q9P2B4	13	8	232	243	25	15	439	340	474	310	291	274	354	300	325
CUBN	O60494	16	4	111	111	113	361	256	401	272	234	83	143	113	153	108
CUL1	Q13616	16	8	304	34	25	148	14	17	45	52	103	63	58	51	60
CUL3	Q13618	11	3	131	6	6	11	23	14	23	42	41	31	21	11	11
CUL4A	Q13619	16	5	302	38	11	6	9	107	10	17	71	20	19	57	20
CUL4B	K4DI93	15	4	298	42	29	28	149	104	66	106	110	94	95	91	79
CUL5	Q93034	8	3	108	3	4	0	3	1	3	10	18	14	8	3	11
CWF19L1	Q69YN2	9	2	75	40	13	15	10	6	10	53	71	43	25	36	48
CYB5R3	P00387	3	2	84	81	187	176	2	6	15	21	23	43	26	26	32
CYCS	C9JFR7	4	3	156	1790	1753	828	462	254	385	242	217	210	320	240	190
CYFIP1	Q7L576	25	2	576	11	13	14	45	39	19	58	37	31	55	59	32
CYR61	O00622	5	4	223	85	175	173	61	136	123	164	162	165	140	251	152
DAB2IP	G3XA90	13	4	124	1047	144	82	254	217	388	207	155	168	267	233	186
DAP3	P51398	5	3	47	25	10	24	13	11	15	20	24	26	37	18	35
DAPK3	O43293	11	7	158	79	43	45	315	220	279	242	275	243	244	253	230
DARS	P14868	29	11	804	1284	1779	2029	835	1196	1278	1487	1148	1562	1404	1334	1498
DAZAP1	Q96EP5	5	4	92	1007	42	89	174	94	121	441	497	390	381	329	476
DBN1	A8MV58	19	8	483	477	567	679	1939	1659	1638	1752	1515	1686	1683	1598	1937
DBNL	Q9UJU6	9	3	411	89	54	124	214	161	79	296	353	345	290	295	270
DCAF13	Q9NV06	14	10	293	384	112	67	137	75	95	138	155	137	132	97	154
DCAF7	P61962	2	2	27	7	13	20	0	0	0	4	13	12	8	4	0
DCD	P81605	4	4	135	2713	2987	2434	1309	1341	1056	868	610	734	921	1038	824
DCDC2	Q9UHG0	4	2	53	8	12	23	7	15	10	22	35	38	30	25	32
DCP1A	Q9NPI6	6	4	64	3	19	15	56	62	47	100	111	114	90	96	118
DCTN1	E7EX90	31	18	621	475	177	234	232	198	288	487	622	607	541	429	538
DCTN2	F5H223	13	7	543	60	60	79	109	88	77	431	417	489	397	329	431
DDB1	Q16531	38	25	986	1309	1852	2264	942	944	1072	2775	2685	2792	2539	2643	3077
DDB2	Q92466	10	5	172	122	52	71	18	24	20	151	189	106	104	86	100
DDOST	P39656	9	4	306	176	36	78	34	9	22	15	51	40	33	26	43
DDX1	Q92499	24	15	578	429	740	659	372	368	319	1308	1489	1228	959	1094	1192
DDX10	Q13206	16	7	283	67	9	124	223	154	278	223	214	210	188	163	222
DDX17	H3BLZ8	41	18	1543	1658	1015	1288	1486	1297	1798	3391	3268	2849	3104	2642	3939
DDX18	Q9NVP1	17	10	666	348	140	230	70	65	89	257	383	190	178	154	196
DDX21	Q9NR30	33	22	1103	3502	1251	1428	1048	577	548	1262	1401	758	882	740	903
DDX23	Q9BUQ8	27	17	550	345	176	215	116	108	134	251	328	193	198	158	261
DDX24	Q9GZR7	19	9	188	56	24	10	66	59	63	80	113	92	91	75	116
DDX27	Q96GQ7	20	15	502	163	92	127	112	75	64	219	297	186	164	126	240
DDX31	Q9H8H2	9	5	94	195	182	300	140	143	167	701	517	649	763	721	711
DDX39A	O00148	27	8	797	179	360	357	44	53	114	262	504	128	183	141	171
DDX39B	F8VQ10	33	3	1175	191	433	703	56	52	173	336	591	305	333	207	399
DDX3X	O00571	31	4	1120	320	213	133	346	361	441	358	425	330	304	359	407
DDX41	J3KNN5	14	3	128	50	30	83	13	8	6	53	65	46	37	26	48
DDX42	Q86XP3	19	11	292	205	323	326	245	240	367	389	365	295	296	261	258
DDX46	Q7L014	26	13	669	336	80	154	71	70	99	98	131	96	76	74	66

DDX47	Q9H0S4	9	3	278	113	102	98	50	120	69	179	191	77	75	84	77
DDX5	B4DLW8	39	10	1498	318	813	893	892	1095	1156	2230	2327	1728	1948	1703	2291
DDX50	Q9BQ39	18	9	326	495	188	102	103	29	39	83	126	91	61	52	96
DDX51	Q8N8A6	9	5	174	925	223	189	136	194	273	147	108	124	106	130	172
DDX54	H0YHH7	8	2	77	1	11	6	1	2	3	9	9	6	5	4	11
DDX54	Q8TDD1	18	9	350	120	77	55	34	29	31	65	117	60	46	31	72
DDX56	Q9NY93	7	3	234	2	0	1	4	1	1	4	8	5	4	3	7
DDX58	O95786	24	11	309	61	29	46	102	81	25	205	208	182	144	147	107
DDX6	P26196	15	13	429	887	346	391	1723	1551	1756	1516	1413	1706	1589	1481	1823
DDX60	Q8IY21	11	4	190	7	21	11	42	4	5	20	45	24	45	32	46
DDX60L	Q5H9U9	13	4	57	56	600	30	12	17	5	92	129	70	99	72	96
DECR1	B7Z6B8	8	3	115	25	10	30	18	27	29	19	14	23	21	14	16
DEK	P35659	18	7	466	558	146	287	1241	1386	1480	1267	1449	1289	1555	1418	1381
DENND5A	Q6IQ26	10	4	81	92	46	37	141	82	84	170	182	201	235	196	202
DERA	Q9Y315	20	7	630	164	91	104	265	225	267	242	303	310	321	245	302
DES	P17661	17	4	592	1072	1798	2081	204	291	400	1275	1910	1969	2544	2933	2463
DFFA	O00273	3	2	19	3	13	21	3	17	34	91	166	135	126	92	150
DFNA5	O60443	9	5	187	310	53	78	99	81	56	176	148	215	134	151	180
DGCR8	Q8WYQ5	5	2	29	2	4	5	42	40	58	42	36	39	31	27	45
DHRS7B	J3KRS1	5	2	57	3476	3366	1448	319	209	384	78	34	46	66	43	37
DHX15	O43143	28	19	907	1219	781	873	182	94	317	651	902	634	460	407	652
DHX16	B0V0F8	10	2	102	24	77	70	27	39	45	81	60	61	90	64	54
DHX30	Q7L2E3	11	3	187	8	7	7	0	6	5	26	42	29	20	16	12
DHX33	Q9H6R0	10	2	114	2	2	3	0	1	1	4	6	5	2	2	4
DHX36	F5GZS0	8	4	74	8	92	52	39	76	39	62	82	71	61	67	50
DHX37	Q8IY37	16	8	226	7463	2857	926	1788	646	970	826	246	338	1140	478	436
DHX38	Q92620	17	5	136	99	106	135	63	200	328	281	310	268	327	226	284
DHX8	F5H658	13	4	181	26	142	176	71	98	101	136	111	115	126	98	118
DHX9	Q08211	63	45	2312	8904	3391	5604	3885	3321	3706	8377	8427	7974	7485	7293	8849
DIAPH1	H9KV28	14	8	262	164	53	64	95	61	47	104	83	91	65	54	61
DIDO1	Q9BTC0	17	8	276	177	15	15	139	7	4	116	67	12	15	25	19
DIEXF	Q68CQ4	10	4	219	243	53	84	10	9	14	10	15	11	6	7	7
DIMT1	Q9UNQ2	7	4	184	117	132	129	12	20	57	22	38	26	37	35	39
DIS3	Q9Y2L1	16	9	377	191	44	92	115	132	184	120	119	107	82	93	113
DKC1	O60832	20	12	437	431	176	290	130	62	86	326	442	268	261	191	372
DKFZP586 J0619	A4D212	26	10	454	82	54	38	16	32	36	26	154	113	105	76	133
DKFZp781 D1416	Q68DW7	19	7	426	403	20	156	288	216	218	559	150	21	503	242	42
DLAT	P10515	9	6	235	90	78	141	24	9	21	25	46	49	23	22	28
DLD	E9PEX6	12	6	184	149	97	139	63	38	56	74	85	79	66	59	81
DLG1	Q12959	16	9	323	1386	1899	597	964	891	999	1098	1213	1291	1241	1195	1253
DLG5	Q8TDM6	22	6	160	59	14	17	149	153	133	84	73	98	56	81	86
DLST	P36957	12	8	453	491	320	566	91	100	134	142	149	193	192	147	223
DMD	E9PDN5	37	6	292	136	31	55	40	24	40	76	83	84	72	55	85
DNAJA1	P31689	9	4	222	145	228	294	155	120	141	226	212	241	249	195	233
DNAJA2	O60884	7	4	117	22	58	72	24	47	35	11	62	60	55	70	52

DNAJB1	P25685	8	2	79	16	4	8	16	23	23	33	22	23	27	19	26
DNAJC13	O75165	26	14	485	617	508	461	3344	5426	8046	2309	2101	2116	2618	2862	2833
DNAJC7	Q99615	9	5	136	44	45	50	176	137	278	198	126	266	157	169	360
DNAJC8	O75937	6	2	58	34	26	33	120	96	117	89	108	88	109	95	115
DNAJC9	Q8WXX5	5	4	78	52	42	37	17	6	26	526	58	25	38	22	29
DNM1	Q05193	13	2	92	6	14	10	8	11	2	13	7	13	10	8	9
DNM1L	O00429	19	3	379	47	29	40	26	18	25	61	71	59	34	44	47
DNM2	F5H4R9	22	6	487	117	73	78	75	77	79	221	222	233	201	191	186
DNMT1	F5GX68	22	4	193	1065	97	97	675	611	776	538	404	472	544	553	485
DNPEP	Q9ULA0	12	5	152	145	30	38	76	59	77	95	122	129	84	88	104
DNTTIP1	Q9H147	5	3	33	38	89	63	466	396	551	211	255	248	250	257	281
DNTTIP2	Q5QJE6	19	3	379	27	6	9	19	13	8	15	10	12	8	12	15
DOCK6	Q96HP0	11	5	133	53	72	205	93	94	91	189	190	186	214	177	211
DOCK7	Q96N67	28	16	690	260	140	134	559	518	450	479	611	652	558	493	576
DOCK9	Q9BZ29-4	18	5	101	41	90	44	96	97	139	99	120	124	133	116	150
DOPEY2	Q9Y3R5	14	3	93	23	20	33	87	100	152	86	75	108	102	90	122
DPF2	J3KMZ8	6	6	207	248	119	92	193	64	214	87	151	87	70	72	84
DPM1	H0Y368	11	2	109	18	17	38	144	209	257	164	132	208	234	263	201
DPYSL2	Q16555	14	6	447	407	130	277	552	407	539	876	712	898	840	925	873
DPYSL3	Q14195	12	8	395	256	318	424	423	451	554	755	751	797	738	728	957
DRG1	Q9Y295	6	3	129	277	423	532	159	238	213	198	165	228	237	226	278
DSC1	Q08554	8	4	73	532	614	422	244	481	224	272	98	160	139	172	173
DSG1	Q02413	11	9	385	291	262	260	280	590	314	383	146	223	143	199	158
DSG2	Q14126	10	6	202	47	33	39	67	61	36	27	30	32	20	23	21
DSP	P15924	110	60	2564	2606	2261	1957	2094	2637	2004	2781	2045	2166	1915	1820	1970
DSTN	F6RFD5	5	2	128	457	220	239	310	224	288	181	192	202	239	197	232
DTX3L	Q8TDB6	11	6	111	614	190	247	111	45	81	190	214	158	167	136	112
DUSP11	O75319	4	2	29	30	3	7	3	1	0	4	7	4	2	4	1
DUSP3	K7ES89	7	2	111	40	9	3	0	0	11	3	6	3	1	0	4
DUT	H0YKC5	5	3	135	8	7	11	1	0	4	2	2	2	1	1	3
DYNC1H1	Q14204	177	109	5152	3520	4221	6150	4751	4621	4841	8082	8124	9385	7742	7365	8138
DYNC1I2	B7ZA04	3	2	88	14	28	21	50	48	65	101	84	112	75	64	99
DYNC1LI1	Q9Y6G9	10	4	138	25	158	105	105	92	109	172	158	202	156	138	218
DYNC1LI2	J3KRZ2	5	2	101	18207	63213	38502	21	82	144	31	20	32	22	35	24
DYNC1LI2	O43237	13	3	281	103	21	32	81	77	73	139	171	227	155	163	201
DYNLL1	P63167	7	5	362	64	176	435	1327	957	730	544	589	548	527	510	419
DYNLL2	Q96FJ2	4	2	215	6	1	3	39	10	20	16	27	30	10	17	9
DYNLT1	P63172	2	2	47	117	103	146	64	61	83	69	73	94	98	103	110
EBNA1BP2	Q99848	11	7	316	300	28	42	50	23	37	115	165	75	63	42	85
ECH1	Q13011	6	2	150	32	35	54	15	15	15	16	18	30	18	10	16
ECI2	O75521-2	8	3	87	29	4	2	1	3	2	10	4	4	7	6	5
EDC3	Q96F86	7	3	144	30	24	11	115	69	25	67	120	74	119	153	59
EDC4	Q6P2E9	24	16	595	2141	2673	792	1463	873	1997	810	778	868	724	625	899
EDF1	O60869	6	3	151	26	32	74	907	907	1152	752	648	927	974	1122	778
EDIL3	O43854	13	9	368	148	167	243	810	1030	1193	921	814	1037	1056	1218	1078
EEA1	Q15075	45	16	897	249	366	364	444	440	486	677	645	673	637	594	677
EED	O75530	7	5	116	8	50	6	9	4	11	30	42	16	34	16	26

EEF1A1	P68104	27	10	984	4099	6693	8135	16435	16154	13395	21036	20910	22473	21507	22405	19480
EEF1B2	P24534	7	5	159	357	400	579	247	238	359	383	434	608	527	432	637
EEF1D	E9PRY8	16	2	455	2	4	5	2	1	1	8	13	13	10	5	7
EEF1G	B4DTG2	16	10	582	944	1885	2302	936	893	1086	1669	1607	1914	1927	1635	1916
EEF2	P13639	47	34	1816	6602	5237	6939	4963	4143	4922	6259	6863	6180	6227	5848	6208
EFHA1	Q8IYU8	4	2	42	5	0	2	4	5	56	5	14	4	2	5	3
EFTUD2	Q15029	43	28	1740	3396	2223	2800	1280	566	1110	2652	3024	1968	2219	1906	2322
EGLN3	F8W1G2	3	2	31	1	3	5	2	1	3	28	31	31	41	26	34
EHD1	Q9H4M9	17	9	413	503	149	147	255	111	110	316	283	331	192	253	153
EHD2	B4DNU6	8	4	143	96	97	64	34	31	52	98	82	111	66	77	72
EHD4	Q9H223	16	4	444	87	82	120	95	92	85	146	105	163	114	135	179
EHHADH	Q08426	6	2	77	61	27	12	13	15	8	25	21	28	26	33	38
EHMT1	Q9H9B1	9	3	106	70	3	6	8	5	7	16	17	12	9	6	28
EHMT2	B0UZY0	8	2	56	9	2	3	1	1	1	10	17	10	8	3	7
EIF1AX	P47813	4	2	64	37	124	150	60	62	71	71	70	79	110	100	69
EIF2A	Q9BY44	10	4	92	195	6	31	108	54	45	135	145	162	129	99	146
EIF2AK2	P19525	8	4	151	54	21	39	31	20	27	72	88	92	67	59	84
EIF2B2	P49770	5	2	72	12	28	87	27	16	17	83	117	78	59	75	44
EIF2C2	Q9UKV8	7	2	78	8	0	1	5	2	2	17	29	26	14	16	28
EIF2S1	P05198	22	3	683	831	1597	1354	607	624	946	649	339	619	721	917	853
EIF2S2	P20042	5	3	106	81	111	266	101	85	121	219	168	226	243	211	247
EIF2S3	P41091	18	10	475	1588	3242	3085	1525	1860	2408	2141	1876	2421	2072	2610	1854
EIF3A	Q14152	48	25	881	639	515	715	498	522	579	1358	1222	1063	1296	1012	1144
EIF3B	P55884	24	15	582	1018	1573	1409	650	672	603	1093	891	1125	946	958	979
EIF3C	H3BRV0	24	14	687	181	162	382	390	322	479	665	610	648	658	557	666
EIF3D	B4DVY1	15	9	311	406	112	351	204	172	153	467	511	695	553	496	609
EIF3E	P60228	19	2	650	14	720	518	344	280	318	335	281	370	354	391	396
EIF3F	B3KSH1	9	8	394	910	996	1284	1290	1210	1739	1224	964	1374	1295	1399	1514
EIF3G	O75821	8	4	127	48	107	142	101	83	64	199	163	258	242	202	251
EIF3I	Q13347	13	7	382	490	602	848	514	564	651	642	696	757	664	761	712
EIF3L	B0QY89	20	8	717	325	502	565	916	767	605	804	658	856	805	879	851
EIF3M	Q7L2H7	15	12	465	728	230	291	1221	952	1068	951	901	1250	1036	1029	1028
EIF3S3	B3KS98	8	6	178	278	661	348	1349	1270	1314	783	832	890	947	931	993
EIF4A1	P60842	26	6	1079	112	116	278	186	174	287	311	354	386	366	317	382
EIF4A3	I3L1H0	11	2	227	19	7	8	18	9	10	22	26	39	33	29	26
EIF4A3	P38919	28	14	1064	462	721	1399	205	193	368	1142	1160	848	1104	773	1199
EIF4G1	D3DNT2	27	9	661	393	127	144	234	176	171	472	424	504	421	442	449
EIF4G2	D3DQV9	20	12	282	135	260	204	265	294	329	395	339	430	359	346	363
EIF4G3	F5H564	21	4	204	49	44	34	39	16	25	61	66	63	59	30	68
EIF4H	Q15056-2	7	5	129	25	66	130	407	506	332	629	759	780	1105	844	821
EIF5	P55010	8	2	85	22	27	16	10	11	9	18	20	16	18	9	20
EIF5A	I3L397	9	2	218	158	124	205	57	60	79	129	110	95	147	119	123
EIF5B	O60841	14	4	103	60	126	185	36	34	50	145	119	120	86	98	109
EIF6	P56537	8	6	251	502	1453	1554	608	560	645	742	817	836	778	587	1066
ELAVL1	B4DVB8	15	7	528	565	477	578	615	515	572	1702	1924	1748	1811	1516	2172
ELMSAN1	Q6PJG2	4	2	46	18	80	56	32	57	32	32	17	18	16	19	20
EMC1	Q8N766-2	13	6	203	184	45	40	435	384	428	123	118	132	108	116	114

EMC3	Q9P0I2	3	2	46	29	117	2	7	83	37	65	89	19	43	122	35
EMD	P50402	4	3	114	20	20	17	6	15	9	13	16	23	5	10	17
EMG1	Q92979	8	6	183	270	32	104	196	188	279	327	407	366	387	372	447
EML4	B5MBZ0	6	3	59	88	153	59	20	23	9	56	63	57	61	47	58
ENAH	Q8N8S7-2	14	7	282	93	117	88	1692	1644	1915	853	726	841	1060	969	1013
ENO1	P06733	36	22	1663	5641	6411	8495	4616	3866	5690	6415	8797	6782	6501	6075	7146
ENO2	B7Z2X9	10	2	295	36	9	39	11	4	10	21	31	21	32	17	35
EP400	Q96L91	12	8	195	24	18	16	385	375	519	421	335	443	362	396	505
EPB41L3	Q9Y2J2-2	26	14	680	427	204	363	500	394	290	419	403	422	361	355	287
EPHA2	P29317	11	4	208	27	30	84	80	78	72	63	55	69	53	51	45
EPM2AIP1	Q7L775	6	2	86	17	6	10	3	4	2	6	8	6	9	8	7
EPPK1	P58107	68	16	2033	426	629	597	692	633	503	686	667	792	487	532	551
EPRS	P07814	53	38	1351	1124	963	1283	1049	964	1149	1556	1848	1867	1537	1414	1869
EPS15	P42566	13	3	173	145	133	55	21	26	53	39	39	44	41	32	34
EPS15L1	Q9UBC2	21	4	538	25	8	13	37	24	19	217	153	185	224	150	166
EPS8	Q12929	7	2	72	5	17	10	28	22	31	38	36	41	43	38	57
ERAP1	Q9NZ08	8	4	136	484	1063	638	260	346	279	233	107	168	151	174	169
ERBB2IP	Q96RT1-8	17	4	132	612	30	18	136	161	100	96	186	92	179	90	120
ERC1	G8JLD3	49	2	1047	1377	73	133	441	178	95	193	164	170	163	144	114
ERC2	O15083	27	2	329	15	8	10	19	16	6	22	22	23	17	14	17
ERCC2	P18074	8	2	61	2	8	5	3	4	0	1	3	7	2	3	3
ERCC3	P19447	7	4	111	47	9	52	34	24	18	9	32	22	23	17	29
ERH	P84090	6	3	154	58	106	107	5	11	6	142	317	167	211	124	179
ERLIN1	O75477	7	2	300	28	24	11	10	1	7	5	14	5	8	3	6
ERLIN2	E5RHW4	11	3	393	10	21	36	6	6	15	26	30	34	26	19	20
ERP44	Q9BS26	4	4	41	63	30	61	6	9	3	20	16	29	15	29	24
ESF1	Q9H501	6	3	58	56	96	69	53	50	81	53	57	50	61	63	59
ESYT1	Q9BSJ8	17	12	529	204	164	247	121	183	205	319	269	362	268	242	313
ESYT2	A0FGR8	9	3	122	60	28	28	76	46	45	83	87	58	70	55	53
ETF1	P62495	9	6	165	52	45	89	90	118	116	66	100	133	134	127	143
ETS1	P14921-4	4	2	90	17	3	2	3	3	4	7	3	4	3	3	5
ETV6	P41212	5	4	121	516	48	49	43	10	8	60	73	46	38	43	48
EWSR1	Q01844	12	7	228	210	300	334	242	262	181	1046	742	606	824	597	911
EXOC1	Q9NV70	10	6	138	351	237	388	116	115	166	187	171	238	221	180	255
EXOC2	Q96KP1	9	3	158	111	61	122	50	47	32	101	89	98	76	79	81
EXOC4	Q96A65	12	11	343	308	71	44	145	142	125	278	249	319	268	207	251
EXOC5	O00471	14	6	155	66	114	97	46	43	55	94	78	99	97	99	89
EXOC8	Q8IYI6	13	5	122	67	15	47	52	42	54	137	128	140	144	138	150
EXOSC1	B1AMU3	5	3	143	66	204	208	47	59	48	48	45	51	50	38	42
EXOSC10	Q01780	24	18	618	537	301	243	164	135	130	310	427	326	289	259	345
EXOSC2	Q13868	9	7	290	396	508	467	163	178	225	262	314	261	212	268	268
EXOSC3	Q9NQT5	7	4	146	68	6	43	22	9	13	35	47	28	21	28	37
EXOSC4	Q9NPD3	6	4	280	69	579	593	121	147	222	131	200	214	281	236	257
EXOSC5	Q9NQT4	6	3	205	695	429	350	92	55	63	76	88	75	85	85	77
EXOSC6	Q5RKV6	7	5	271	416	716	910	116	114	129	223	233	258	288	304	288
EXOSC7	Q15024	7	6	105	534	292	272	221	113	93	174	193	129	168	212	145
EXOSC8	Q96B26	9	4	138	116	250	227	22	34	33	49	58	55	44	52	66

EXOSC9	D6RIY6	7	2	148	44	99	173	22	22	35	83	87	58	59	53	61
EZH2	Q15910	6	3	94	304	21	16	33	11	11	23	13	16	20	11	12
EZR	P15311	48	18	1712	5239	3011	3622	2876	2259	3142	3058	4431	3281	2937	3144	3486
F8	P00451	14	3	104	277	3	22	41	17	11	24	27	31	26	10	78
FAM120A	Q9NZB2	17	9	528	100	153	227	121	135	137	375	488	548	497	386	527
FAM129A	Q9BZQ8	3	2	129	25	11	38	100	63	83	58	49	48	40	43	45
FAM129B	Q96TA1	10	5	132	69	13	87	165	132	103	237	211	222	222	197	212
FAM162A	F8W7Q4	2	2	26	7	2	2	3	1	0	51	56	38	48	29	25
FAM175B	Q15018	3	2	31	39	4	26	16	13	5	31	31	37	31	31	27
FAM192A	Q9GZU8	5	2	57	16	14	68	1	3	0	3	9	21	2	1	40
FAM208A	Q9UK61	8	3	95	69	26	30	214	169	239	161	151	222	197	160	217
FAM208B	Q5VWN6	20	3	138	4	3	7	68	211	140	47	50	66	75	57	68
FAM21C	Q9Y4E1	6	4	63	86	8	19	18	12	18	45	50	40	33	27	51
FAM49A	Q9H0Q0	8	2	147	73	112	75	15	17	20	15	20	23	25	13	18
FAM50B	Q9Y247	7	2	63	46	10	1	15	13	6	21	18	21	16	13	14
FAM65A	Q6ZS17-4	13	5	90	59	212	280	150	166	320	159	329	421	343	300	314
FAM91A1	E7ER68	5	3	26	34	17	5	12	8	3	12	16	17	10	10	6
FAM98A	Q8NCA5	5	4	153	12	17	23	23	20	25	118	116	112	112	95	106
FAM98B	Q52LJ0	8	4	427	173	462	499	243	263	370	387	321	292	351	405	397
FANCI	Q9NVI1	9	2	136	1	17	12	1257	4	10	267	0	0	13	1	1
FARP1	Q9Y4F1	13	6	174	48	97	32	264	220	285	308	270	358	278	358	291
FARSA	K7EPH2	10	3	96	22	6	8	9	9	160	75	93	63	61	48	68
FARSA	B4E363	6	2	67	877	72	44	258	33	8	66	24	29	30	22	6
FARSB	Q9NSD9	13	9	351	76	82	93	143	116	132	204	188	244	156	164	153
FASN	P49327	49	35	1128	514	477	642	744	532	493	626	566	579	618	409	459
FBL	P22087	21	12	631	682	1424	1654	119	110	431	619	898	552	548	469	770
FBLIM1	Q8WUP2	10	7	228	55	91	71	2044	1853	2468	736	698	719	916	883	790
FBN1	P35555	12	9	125	23	86	68	408	356	662	457	449	525	606	577	532
FBXL18	H0Y6I6	4	2	36	20	5	4	21	11	1	34	45	30	48	41	12
FBXO22	Q8NEZ5	8	4	163	292	239	62	8	9	9	36	46	34	35	28	39
FBXO28	Q9NVF7	9	2	123	25	92	99	206	231	226	176	145	193	163	171	200
FCF1	G3V1S4	4	2	109	2	32	22	1	1	3	18	28	12	20	10	25
FCHO2	Q0JRZ9	7	2	68	0	5	5	4	1	3	18	18	23	22	20	22
FDPS	P14324	7	4	35	125	128	323	66	59	56	45	132	38	135	130	26
FEN1	P39748	12	5	314	91	109	288	193	150	473	355	457	411	213	208	333
FERMT2	H0YJ34	13	7	351	66	8	54	340	410	311	237	212	295	342	325	269
FGF2	P09038	8	7	215	364	289	478	254	224	270	469	464	426	614	478	471
FH	P07954	8	5	134	126	65	148	28	14	35	103	130	78	37	50	45
FHL2	J3KNW4	18	11	559	193	327	485	3336	3647	4296	2503	2790	2256	3359	2910	2732
FHL3	Q13643	3	3	94	38	4	8	164	157	170	67	64	57	87	79	77
FHOD3	K7EKZ0	4	2	40	16	2	2	23	160	6	57	123	78	118	114	67
FHOD3	K7ER94	10	3	156	105	4	5	22	22	33	45	55	61	52	54	74
FIP1L1	Q6UN15	13	7	282	140	86	269	62	26	62	163	257	137	91	63	152
FKBP15	Q5T1M5	15	11	348	237	105	66	57	53	31	162	181	163	129	106	139
FKBP3	Q00688	5	2	137	20	42	55	104	89	108	137	164	146	212	154	170
FKBP4	Q02790	5	2	108	182	36	4	14	0	6	11	14	11	8	6	7
FLG	P20930	19	6	151	46	26	63	27	22	22	53	54	15	38	53	45

FLG2	Q5D862	6	3	129	22	530	518	409	418	248	269	137	187	141	157	176
FLII	Q13045	33	17	710	1807	300	289	955	910	789	706	899	646	537	669	513
FLNA	P21333	165	98	7418	16964	33005	36745	137430	126907	136438	63601	54963	59340	69322	72300	71334
FLNB	O75369-8	163	2	6595	5	9	4	139	120	126	21	36	43	59	44	65
FLNC	Q14315	109	65	3990	3389	3007	3047	15633	14171	12694	9775	8444	9402	9977	10563	10144
FLOT1	O75955	18	4	619	13	42	84	146	196	255	42	55	68	51	47	60
FLOT2	J3QLD9	22	13	551	292	63	99	368	322	267	116	145	127	79	74	81
FMNL2	Q96PY5-3	12	4	115	160	14	19	95	75	88	128	111	113	104	130	130
FMR1	G8JL90	7	4	96	14	56	48	65	70	90	357	460	342	334	257	380
FN1	E9PE77	95	81	3729	16126	23363	19329	127814	161700	176925	127652	96351	129454	149613	165897	127749
FNBP1L	Q5T0N5-4	13	2	94	854	22	46	182	53	68	92	41	56	76	60	50
FNDC1	Q4ZHG4	13	3	93	8	15	15	5	6	53	10	19	15	22	13	45
FNDC3B	Q53EP0	17	15	371	540	78	163	102	49	72	221	255	236	206	159	210
FRG1	Q14331	2	2	86	55	2	16	103	129	37	109	77	86	56	59	64
FRMPD3	Q5JV73	18	5	103	100	79	139	153	143	74	246	356	263	279	315	296
FRYL	F5GX82	19	5	222	17	22	28	9	5	14	79	86	70	69	73	85
FSCN1	J3KNT0	12	8	308	628	1133	1418	749	755	1167	894	958	932	1025	851	992
FTH1	P02794	3	2	90	9	7	11	20	29	10	52	95	39	81	50	72
FTL	P02792	8	4	116	21	40	45	81	64	60	128	186	220	210	175	173
FTSJ3	Q8IY81	27	14	748	2017	883	377	236	91	100	273	393	183	174	145	214
FTSJD2	Q8N1G2	3	3	55	35	45	26	5	4	5	10	19	11	9	6	12
FUBP1	B4DT31	16	10	550	242	376	436	236	285	344	2248	2324	1563	1828	1459	2445
FUBP3	Q96I24	19	11	389	94	118	129	184	144	114	886	1028	795	844	652	1153
FUS	H3BPE7	6	4	185	83	116	123	215	213	301	827	695	780	681	470	1312
FUT8	Q9BYC5	6	3	131	8	5	9	9	4	5	18	25	20	14	13	8
FXR1	P51114	16	9	237	75	90	181	519	506	369	675	618	723	665	689	749
FXR2	P51116	11	6	167	289	49	82	50	29	28	109	168	101	81	76	97
FYTTD1	Q96QD9-2	9	6	167	283	53	101	126	97	94	128	151	111	112	114	134
G3BP1	Q13283	16	13	482	1315	2216	2412	3126	3697	2998	4097	4357	4655	4851	4677	4918
G3BP2	Q9UN86	11	7	340	105	178	189	438	426	277	834	1173	908	679	970	672
G6PD	P11413	15	4	222	296	29	41	66	18	38	69	54	75	66	62	69
GAK	E9PGR2	10	6	129	32	15	43	33	29	35	68	79	111	79	58	76
GALNT2	Q10471	9	7	155	216	45	63	24	19	28	46	81	53	23	24	25
GAPDH	P04406	32	25	1394	21809	29459	41931	20962	17616	25472	21668	28599	26766	25152	23000	25517
GAPVD1	Q14C86	11	4	101	16	5	64	19	9	17	37	18	23	17	10	33
GAR1	Q9NY12	6	2	128	148	99	124	25	16	32	110	209	128	139	118	113
GARS	P41250	16	8	273	246	248	250	211	173	216	310	446	391	281	289	271
GART	P22102	21	13	294	389	741	1093	532	462	399	529	649	650	496	497	540
GATAD2A	B5MC40	13	6	418	55	7	16	18	10	29	61	74	29	34	27	43
GATAD2B	Q8WXI9	12	9	206	1056	1316	1158	80	29	29	110	142	78	77	40	99
GBE1	Q04446	7	3	59	8	3	7	11	11	3	43	57	52	29	29	30
GBF1	Q92538	13	2	276	169	17	10	37	4	5	18	5	10	7	8	4
GBP1	P32455	15	6	170	88	22	22	104	100	134	86	99	122	86	73	127
GCC2	Q8IWJ2	23	9	248	247	148	169	339	334	377	247	227	255	340	322	312
GCN1L1	Q92616	45	21	1001	729	291	334	141	140	203	468	548	517	380	344	446
GDF15	Q99988	10	6	147	2745	4958	3674	2861	2876	2952	1712	1529	1652	1834	2395	1938
GDI2	E7EU23	17	2	256	142	201	235	61	48	61	52	59	55	63	51	54

GEMIN5	Q8TEQ6	7	2	105	476	349	389	404	527	506	353	152	209	201	230	200
GFPT1	Q06210	17	10	505	226	139	221	1436	321	295	464	478	473	424	433	401
GFRA1	P56159	4	2	84	25	14	10	7	22	10	20	12	9	17	17	17
GFRA3	O60609	2	2	17	353	1553	1206	547	689	489	533	688	650	575	662	521
GIPC1	O14908	8	3	94	27	43	55	27	23	11	46	62	81	59	51	34
GIT1	Q9Y2X7-3	11	2	182	18	6	15	195	182	248	102	98	120	128	90	143
GLE1	Q53GS7	16	6	241	157	22	19	40	30	27	56	70	58	77	67	57
GLG1	Q92896	13	7	246	26	16	26	32	47	45	35	35	35	38	21	28
GLIPR2	Q9H4G4	7	6	253	463	1470	1223	3350	3791	4070	1832	1850	2281	2289	2909	2115
GLOD4	Q9HC38-2	3	2	46	95	17	51	10	14	15	15	8	18	20	10	18
GLRX3	O76003	7	4	93	308	79	89	43	30	35	71	102	73	59	37	62
GLS	O94925-3	17	2	492	18	7	37	41	97	42	105	45	165	123	146	98
GLS	O94925	18	2	453	7	25	147	1074	1418	1578	783	880	900	986	830	1094
GLTSCR2	Q9NZM5	4	2	105	91	0	2	12	1	0	9	8	3	6	3	2
GLUD1	P00367	13	3	233	75	7	87	32	15	25	9	14	10	17	10	10
GLYR1	Q49A26	18	9	649	69	70	150	17	13	60	148	284	121	114	92	156
GMPS	P49915	12	7	258	134	152	229	52	64	99	114	139	96	90	88	139
GNA13	Q14344	5	3	64	2355	12	18	194	15	1095	31	11	15	12	15	31
GNAI2	P04899-4	12	5	349	177	124	184	111	111	98	164	232	228	161	164	136
GNAS	Q5JWF2-2	12	3	124	23	25	56	13	6	10	14	14	20	13	12	12
GNG12	Q9UBI6	4	2	71	15	155	136	92	89	77	43	62	54	81	114	34
GNL2	H0YG10	9	2	121	49	44	104	71	70	98	97	93	104	105	97	106
GNL2	Q13823	13	4	348	91	53	106	41	29	37	62	73	70	65	67	70
GNL3	Q9BVP2	18	12	419	164	172	182	47	39	68	178	259	158	93	84	171
GNPDA1	D6R9P4	4	2	27	7	23	8	37	36	26	51	69	58	35	41	58
GNPNAT1	G3V5E4	2	2	77	18	4	1	14	10	7	11	18	19	18	17	11
GOLGA3	Q08378	29	17	668	208	104	128	185	150	158	379	403	404	375	287	404
GOLGA4	Q13439	38	12	613	367	60	143	335	323	346	338	352	371	474	393	401
GOLGB1	Q14789	81	19	1532	322	291	182	104	127	92	241	267	244	221	157	195
GOT2	P00505	7	3	212	257	125	294	33	19	57	28	25	35	43	33	47
GPATCH8	Q9UKJ3	12	3	67	12	12	36	21	18	19	43	43	44	39	42	31
GPI	P06744-2	19	9	436	455	361	816	394	350	383	471	506	552	572	506	622
GPS1	C9JFE4	9	4	108	218	48	52	44	22	40	55	50	41	47	40	41
GPX1	P07203	4	4	60	224	111	161	136	66	75	237	243	299	312	283	331
GPX4	K7ERP4	6	3	74	15	34	37	46	42	51	77	76	84	80	80	63
GREM1	O60565	7	6	138	94	91	104	64	55	71	333	517	322	346	305	281
GRIPAP1	Q4V328	13	7	218	1397	799	475	555	665	589	343	267	262	222	261	260
GSN	P06396-2	29	18	911	1653	1894	2186	2673	2965	2566	4153	5172	5384	4662	4933	5032
GSR	P00390	5	4	99	35	33	118	29	30	29	57	62	70	51	59	83
GSS	B6F210	5	2	61	25	27	40	228	130	91	178	108	193	236	237	210
GSTK1	E9PFN5	7	2	87	25	1	13	13	9	14	14	17	21	27	26	28
GSTO1	P78417	7	4	88	47	58	114	40	34	71	38	49	51	50	33	56
GSTP1	P09211	10	7	331	1211	1221	2271	758	708	1420	821	912	932	978	845	971
GTF2B	B1APE1	2	2	34	3	4	5	18	17	26	11	17	17	18	14	15
GTF2E1	P29083	5	2	81	5	5	14	4	2	4	16	24	23	13	6	23
GTF2F2	P13984	10	6	139	281	133	282	56	65	122	238	206	161	129	126	90
GTF2H1	P32780	9	4	72	22	28	69	4	13	6	8	39	38	36	30	35

GTF2I	P78347	33	17	752	246	195	240	464	719	623	981	1008	825	702	641	860
GTF3C1	Q12789	29	7	354	93	12	47	84	45	85	132	174	153	180	122	164
GTF3C2	Q8WUA4	6	3	61	80	171	189	18	32	66	210	345	203	96	152	81
GTF3C3	Q9Y5Q9	7	4	142	30	20	28	7	6	6	25	29	14	15	16	20
GTF3C4	Q9UKN8	5	3	112	3	10	13	3	4	3	13	20	14	15	11	13
GTPBP4	Q9BZE4	31	19	791	772	386	678	205	166	199	644	813	529	528	388	584
GULP1	Q9UBP9	7	3	179	24	47	22	17	20	22	79	85	96	107	88	79
H1FX	Q92522	4	3	64	19	42	12	19	34	22	12	16	11	48	14	14
H2AFY	O75367	20	15	1097	1519	4474	5245	1027	1840	1253	4083	5485	3692	3893	3231	5659
H2AFY2	Q9P0M6	17	6	475	496	85	150	63	22	28	312	405	227	267	185	282
HADHA	P40939	24	17	828	458	194	409	1230	1361	972	1653	1460	2131	1677	2152	2097
HADHB	P55084	14	10	366	1604	408	434	1025	996	1049	1017	877	1183	1131	1399	1448
HARS	D6REN6	17	3	269	137	136	411	2036	2010	2966	973	1204	1407	1162	1157	1568
HAT1	O14929	12	6	445	169	74	236	33	30	36	119	195	103	88	67	111
HAUS1	Q96CS2-2	5	2	63	24	222	206	2255	1931	1309	677	703	933	1056	920	1398
HAUS4	Q9H6D7	5	2	99	8	10	10	27	22	23	12	14	20	11	19	10
HAUS5	O94927	7	4	78	25	36	48	142	160	157	81	75	88	132	130	105
HAUS6	Q7Z4H7	12	5	73	40	31	56	124	141	124	106	71	80	122	114	129
HAUS7	Q99871	4	2	35	18	34	25	29	13	17	12	14	14	7	17	53
HBA1	P69905	5	3	98	1484	97	25	184	76	141	141	101	220	458	194	155
HBG2	E9PBW4	3	2	49	2837	28	31	277	17	20	157	117	211	478	197	140
HCFC1	P51610	22	15	376	311	72	111	84	59	108	210	293	182	150	114	225
HDAC1	Q13547	20	10	557	227	195	912	162	75	117	385	466	401	331	289	458
HDAC2	B3KRS5	15	7	490	78	191	191	67	49	48	185	285	185	193	152	229
HDGF	P51858	5	2	66	37	111	165	11	14	24	101	190	112	74	72	73
HDGFRP2	Q7Z4V5	7	4	123	27	37	15	13	22	12	49	41	31	17	20	202
HDLBP	H7C0A4	20	2	461	91	197	108	21	37	65	37	46	54	64	44	61
HEATR1	Q9H583	46	34	1283	298	273	444	95	135	907	294	574	257	271	206	302
HECTD1	Q9ULT8	17	2	108	0	2	9	11	11	16	21	16	16	22	14	12
HECTD4	J3KPF0	18	2	99	24	67	55	119	155	178	72	55	82	92	101	105
HELZ2	Q9BYK8	41	7	870	27	29	23	19	22	28	85	105	116	93	64	133
HERC2	O95714	27	4	175	40	56	81	148	111	66	169	175	228	158	162	214
HERC6	Q8IVU3	5	3	37	264	79	51	6	10	21	18	62	60	56	37	42
HEXIM1	O94992	2	2	66	17	2	6	1	4	0	7	12	5	4	4	2
HGS	O14964	7	4	136	12	38	60	41	36	40	81	60	83	60	54	69
HIP1R	O75146	18	7	252	30	26	21	67	69	43	143	148	176	137	145	145
HIST1H1B	P16401	10	7	418	275	320	362	96	97	142	606	693	357	452	255	496
HIST1H3A	P68431	16	2	434	8668	9383	29896	1437	1724	2945	775	4233	975	456	947	104
HIST1H4A	P62805	15	15	1184	23248	62809	57243	3651	5286	14070	58176	72423	53703	63479	48519	65007
HIST2H2A A3	Q6FI13	9	3	445	57509	3551	3489	2078	529	7048	3541	4242	4363	4261	3228	2758
HIST2H3A	Q71DI3	16	2	449	4635	5655	19839	1041	1181	4661	473	2007	1421	364	470	90
HK1	E7ENR4	13	3	241	0	5	10	25	25	25	14	21	22	17	19	28
HKDC1	Q2TB90	14	4	266	13	9	3	11	11	14	6	9	7	10	4	15
HLA-A	A9R9N7	12	3	356	50	109	146	49	39	55	52	49	60	43	43	40
HM13	Q8TCT9-5	2	2	20	75	8	5	11	2	0	12	8	11	11	9	5
HMGA2	Q1M185	3	2	42	58	58	26	7	9	33	79	38	17	20	29	55

HMGB1	P09429	10	4	408	93	297	298	173	171	275	510	602	416	607	388	511
HMGB2	P26583	8	4	305	16	23	28	125	106	145	135	183	141	168	136	111
HMGB3	E7EQU1	3	2	110	12	43	72	142	104	164	127	156	135	193	159	164
HMGCL	P35914	3	2	49	1	7	5	0	1	1	1	5	1	8	3	13
HNRNPA0	Q13151	15	12	548	1421	1919	2354	348	374	667	2005	2855	1667	1684	1548	2003
HNRNPA1	F8VRQ1	24	3	1233	1872	5905	6260	412	531	2052	2381	4743	2821	1824	2417	3056
HNRNPA2 B1	P22626	33	23	1295	10017	25373	34066	5437	5899	13830	13179	17960	13529	14090	14116	17057
HNRNPA3	P51991	26	20	1151	2103	3004	4621	1307	1227	1947	6900	8476	5202	6004	5320	7361
HNRNPAB	D6R9P3	14	11	515	1886	1079	1441	569	412	739	1195	1491	1191	1216	1095	1611
HNRNPC	G3V2Q1	24	2	1272	281	198	1328	47	52	299	67	259	55	74	37	47
HNRNPD	Q14103	23	17	742	1445	3273	3561	1243	1186	1621	3662	5033	3233	3448	3078	4161
HNRNPF	P52597	23	13	766	1575	2441	4166	218	285	826	1215	1671	1048	1188	850	1368
HNRNPH1	G8JLB6	22	4	911	1195	5738	5580	158	383	1503	714	940	876	706	697	913
HNRNPH2	P55795	24	9	853	551	162	445	286	67	83	300	356	357	312	249	307
HNRNPH3	P31942	14	10	542	2507	3273	3159	552	232	741	576	350	655	372	388	601
HNRNPK	P61978	37	2	1837	101	64	103	27	35	68	60	268	5	18	38	8
HNRNPL	P14866	28	2	1053	17	36	20	37	24	24	30	53	23	54	33	21
HNRNPL	M0QXS5	28	2	1012	565	45	89	152	68	68	263	250	276	283	221	205
HNRNPM	P52272	88	27	3577	1614	1949	1853	764	683	878	3535	3960	2614	2931	2250	3432
HNRNPR	O43390	32	20	1213	1002	2408	2308	362	424	740	3882	4853	2952	3330	2615	3949
HNRNPU	Q00839	56	34	2204	4847	10260	10786	1074	1467	3680	12297	16701	10059	8270	7816	9476
HNRNPUL 2	Q1KMD3	42	27	1172	1953	1098	1818	657	379	478	1754	2062	1255	1440	1264	1514
HNRPDL	O14979	21	8	529	464	357	498	144	111	137	269	347	288	268	212	345
HNRPLL	B7WPG3	5	3	65	31	24	21	522	501	408	383	351	318	403	326	346
HOOK3	Q86VS8	7	2	90	25	21	14	10	3	7	27	21	28	29	15	18
HP1BP3	Q5SSJ5	19	12	607	869	230	314	174	77	119	1096	1231	784	734	577	1023
HPRT1	P00492	4	2	88	2	5	4	27	32	15	17	24	24	18	17	19
HPS3	Q969F9-2	9	2	60	20	10	13	4	4	1	12	24	12	9	10	12
HRAS	P01112	5	4	99	40	63	75	168	235	206	149	203	164	186	190	130
HRNR	Q86YZ3	11	8	232	846	900	201	466	342	160	350	119	148	83	139	85
HSD17B10	Q99714	5	5	201	60	23	133	18	22	20	23	54	44	42	24	31
HSD17B12	Q53GQ0	6	2	69	11	19	14	62	99	70	122	156	152	163	148	118
HSP90AA1	P07900	49	17	1932	1702	1878	2970	1111	1022	1394	1835	1976	2145	1696	1648	1893
HSP90AB1	P08238	50	11	2064	2151	1880	2833	1721	1477	1882	2190	2148	2336	2128	1874	2275
HSP90B1	P14625	21	9	426	1328	1497	1843	192	211	295	236	269	321	296	221	272
HSPA1A	P08107	27	5	1226	197	297	266	147	120	126	378	383	418	367	289	424
HSPA2	P54652	23	2	997	15	17	20	19	9	5	43	52	30	30	26	33
HSPA4	P34932	20	9	581	489	195	293	160	118	128	340	381	357	298	247	308
HSPA5	P11021	31	25	1379	5888	5332	7919	1817	1824	2869	2858	3677	3947	3727	2793	3877
HSPA6	P17066	16	2	715	3	3	10	2	5	4	10	19	13	10	19	17
HSPA8	P11142	54	28	2850	17433	22236	26582	17575	15291	15192	20230	19611	19443	20759	19140	22280
HSPA9	P38646	27	9	704	717	688	1010	122	132	158	228	196	272	268	176	227
HSPB1	P04792	5	2	175	59	26	86	55	67	59	106	81	152	152	147	138
HSPBP1	Q9NZL4	3	2	81	1	1	7	2	3	2	7	8	4	4	3	7
HSPD1	P10809	26	13	676	1332	949	2049	723	313	534	458	334	460	339	243	395

HSPG2	P98160	69	52	1898	2046	622	720	8599	9433	9603	5943	4197	6098	5985	7513	5651
HSPH1	Q92598	22	2	558	61	21	29	44	38	33	96	109	134	63	73	85
HTATSF1	O43719	7	6	65	35	30	68	92	68	72	177	133	212	219	129	202
HUWE1	Q7Z6Z7	32	4	447	165	394	328	2797	3412	3488	1873	2382	2151	3087	2956	2381
HYOU1	E9PL22	7	2	125	79	4	4	15	9	12	46	42	41	66	51	43
IARS	P41252	38	22	989	1533	909	926	1069	564	565	1258	1228	1303	1120	1197	1191
ICAM1	P05362	4	2	61	10	6	14	39	40	76	32	28	29	30	33	38
ICT1	Q14197	5	4	68	59	75	84	49	49	35	57	45	54	57	35	60
IDH2	P48735	7	3	92	14	6	6	14	7	13	32	33	48	39	34	32
IFFO2	Q5TF58	3	2	22	86	5	4	20	6	4	6	15	5	8	6	15
IFI16	Q16666-2	25	13	450	401	181	170	302	328	409	703	716	686	596	575	744
IFI35	P80217	7	4	81	708	208	198	86	69	14	64	46	74	68	84	50
IFIT1	P09914	20	14	478	275	169	280	211	126	216	355	353	379	416	308	381
IFIT2	P09913	6	6	215	697	13	31	96	21	22	36	43	50	43	43	34
IFIT3	O14879	11	9	454	225	578	239	174	120	129	184	187	238	251	198	236
IGF2BP1	Q9NZI8	21	10	543	204	463	457	535	517	423	1366	1356	1453	1581	1286	1671
IGF2BP2	F8W930	19	5	577	41	29	32	70	56	57	218	233	267	271	232	284
IGF2BP3	O00425	18	8	540	193	134	117	274	155	127	692	865	833	728	615	871
IGF2R	P11717	20	10	276	142	205	268	93	98	134	148	147	205	161	154	206
IGFBP7	Q16270	12	9	220	54	70	90	489	696	874	369	453	416	574	626	521
IK	Q13123	14	5	245	287	50	63	63	64	65	52	124	75	79	74	102
IKBIP	Q70UQ0-4	17	11	466	711	633	758	206	181	221	585	646	690	667	505	678
IKBIP	Q70UQ0	14	2	307	56	29	40	4	4	4	7	11	19	14	8	13
IKBKB	O14920-2	10	5	163	3112	2995	4483	888	904	1342	965	1062	1035	1301	1256	1643
IL15RA	H0YD11	2	2	17	33	10	15	5	5	3	0	2	5	6	3	5
IL1A	P01583	5	3	182	25	32	29	32	49	45	40	34	32	42	40	21
IL4I1	Q96RQ9	7	5	253	28	11	19	19	26	47	46	68	72	91	77	80
ILF2	Q12905	25	21	1051	7958	12301	13047	2288	2307	5480	8247	10203	6849	7680	6701	9888
ILF3	G5E9M5	48	17	1562	994	1121	1480	468	421	704	3750	3932	2563	2641	2318	3601
ILK	Q13418	21	13	471	206	164	218	1531	1213	1511	1162	1040	1261	1344	1332	1295
ILKAP	Q9H0C8	10	2	195	8	5	8	3	3	7	27	28	26	26	10	32
IMMT	B9A067	33	2	886	4	13	10	21	11	4	37	27	12	14	23	13
IMP3	Q9NV31	10	7	213	298	77	105	137	70	94	151	200	137	174	142	160
IMP4	E7ENR5	11	6	187	605	71	70	67	85	53	97	125	83	67	66	66
IMPDH2	H0Y4R1	19	14	562	186	332	487	437	334	446	366	378	407	389	313	322
INA	Q16352	23	9	707	109	85	252	127	102	148	190	192	227	234	187	267
INF2	Q27J81	13	3	139	21	37	8	10	9	9	30	20	20	21	18	16
INO80	Q9ULG1	16	6	154	29	15	26	96	96	119	122	134	159	165	153	161
INTS2	J3KMZ7	7	3	52	4	36	72	3	2	13	45	40	44	31	25	43
INTS4	Q96HW7	15	3	174	40	5	12	16	2	19	9	13	10	13	3	9
INTS5	Q6P9B9	7	4	162	29	54	46	28	34	65	31	38	41	31	21	36
INTS6	Q9UL03	9	3	53	7	10	5	3	1	6	14	17	13	8	8	17
INTS7	Q9NVH2	6	4	214	21	2	4	4	4	5	8	11	7	2	1	4
IPO11	F8WDV0	7	5	151	68	58	55	13	14	37	29	49	38	36	24	34
IPO4	Q8TEX9	10	5	293	85	6	2	21	5	20	14	11	15	8	6	11
IPO7	O95373	23	16	576	740	381	917	263	222	687	446	566	487	406	334	691
IPO8	O15397	11	5	143	125	15	28	33	13	16	48	54	40	34	28	33

IPO9	Q96P70	13	10	330	465	325	391	228	232	244	262	294	306	275	236	292
IQGAP1	P46940	126	91	5424	28136	17317	18721	58443	52010	49045	49209	51594	48037	42999	44821	45189
IQGAP3	F2Z2E2	22	12	469	57	99	105	296	303	263	102	89	93	99	82	95
IRF2BP1	Q8IU81	3	2	26	7	1	1	5	4	5	14	18	11	10	6	27
IRF2BP2	Q7Z5L9	6	2	68	28	5	8	26	25	35	41	39	43	33	26	46
IRF2BPL	Q9H1B7	11	3	286	155	202	190	45	47	107	57	69	82	61	128	822
ISG15	P05161	8	6	352	476	655	614	596	597	583	1742	2415	1711	2304	1826	2142
ISG20	Q96AZ6	8	4	150	104	35	14	335	305	385	280	303	382	638	542	602
ISY1	Q9ULR0-1	9	4	185	367	7	8	43	19	21	31	39	22	25	14	27
ITCH	Q96J02	4	2	51	66	8	0	7	4	2	7	7	6	7	4	4
ITGA1	P56199	8	5	148	34	30	6	480	524	434	123	78	91	96	121	96
ITGA2	E7EMF1	7	3	108	5	5	1	171	160	219	16	9	18	12	23	13
ITGA3	P26006	17	10	378	74	65	75	130	76	118	232	211	226	250	291	260
ITGA5	P08648	9	4	235	30	15	31	856	813	1062	295	179	297	265	328	269
ITGAV	P06756	10	4	107	4	14	29	197	195	278	120	116	120	147	161	164
ITGB1	P05556	15	9	411	173	176	275	1944	2030	2659	764	582	803	895	964	908
ITPR1	E7EPX7	24	4	152	5296	17	20	603	49	41	125	57	64	74	66	65
ITPR2	Q14571	28	7	296	83	204	112	93	111	147	85	119	139	105	86	108
ITPR3	Q14573	36	14	452	83	63	63	61	85	81	106	131	124	111	92	105
ITSN2	Q9NZM3	16	4	145	12	7	24	22	16	33	244	225	142	219	137	181
IVNS1ABP	Q9Y6Y0	4	3	59	17	2	5	27	10	11	21	25	22	12	12	18
IZUMO2	M0R2L8	3	3	32	95	7	14	11	17	13	232	221	185	223	154	173
JUP	P14923	30	9	1079	698	306	470	682	959	753	937	676	640	587	631	565
KALRN	H7BXZ5	17	3	115	57	31	160	150	76	18	140	116	171	108	146	138
KANK1	Q14678	14	2	121	12	18	3	55	42	27	45	53	54	47	49	30
KANK2	Q63ZY3	31	18	634	287	360	243	1846	1739	1683	1500	1166	1773	1733	1903	1727
KARS	Q15046-2	23	13	433	880	220	327	289	226	269	448	426	470	440	374	492
KAT7	O95251-5	8	3	109	14	8	4	0	5	8	30	38	33	19	12	33
KDM1A	F6S0T5	12	10	349	184	41	114	34	26	24	125	165	102	93	63	109
KHDRBS1	Q07666	8	2	157	38	95	54	17	41	30	349	481	264	265	263	405
KHDRBS3	O75525	6	2	76	11	28	4	5	5	26	74	93	57	68	42	76
KHSRP	Q92945	31	9	979	1324	183	170	180	34	45	620	633	329	319	244	470
KIAA0020	Q15397	28	15	737	821	495	475	423	369	416	1460	1142	1212	1771	1374	1539
KIAA0196	Q12768	19	5	290	50	26	28	49	53	56	109	90	122	95	96	100
KIAA0368	J3KN16	39	25	816	314	111	312	207	170	280	287	348	338	277	248	299
KIAA1033	Q2M389	9	3	112	22	17	32	23	13	15	66	72	96	74	61	79
KIAA1109	Q2LD37	28	7	155	414	127	139	202	243	303	233	183	251	271	261	244
KIAA1429	Q69YN4-3	22	10	430	54	55	52	49	29	32	84	93	51	77	46	61
KIAA1524	Q8TCG1	8	2	73	5	5	8	33	20	27	12	15	15	10	10	8
KIAA1731	Q9C0D2	21	2	182	373	17	49	204	133	199	213	203	210	283	165	234
KIAA1967	Q8N163	32	20	1105	3196	1787	1951	868	421	676	2076	2430	1636	1633	1389	2034
KIDINS220	Q9ULH0	17	2	125	2	1	3	7	9	9	18	13	19	19	18	24
KIF1C	O43896	13	2	110	2	33	63	1	21	14	18	38	24	28	25	17
KIF23	H7BYN4	12	3	137	29	34	19	6	3	4	1	3	3	3	5	1
KIF2A	O00139	17	4	373	12	41	36	65	59	97	135	146	124	127	98	137
KIF2C	B7Z6Q6	10	3	60	126	213	172	905	1085	508	908	644	958	1032	1315	869
KIF3B	O15066	10	2	80	4	2	14	195	22	195	57	16	204	35	25	34

KIF4A	O95239	22	2	227	5	9	0	1	3	0	3	5	9	2	1	0
KIF4B	Q2VIQ3	21	2	120	12	3	43	20	37	24	25	18	28	24	26	39
KIF5B	P33176	34	19	1180	449	615	926	733	621	614	1140	1010	1072	1090	889	917
KIF5C	O60282	17	2	420	10	2	1	9	0	23	15	2	16	0	0	3
KIN	F5GXB3	7	2	77	29	3	10	9	3	8	13	15	13	9	6	10
KLC1	F8W6L3	13	6	324	100	82	122	122	78	133	184	197	235	194	174	224
KLHL5	H0Y9Y5	8	2	46	33593	18399	37657	14099	18350	12297	9480	5286	7054	11131	11955	11559
KNTC1	P50748	18	3	87	2	2	2	20	20	12	7	7	9	5	4	4
KPNA2	P52292	11	7	238	169	88	244	207	164	246	166	162	259	126	114	203
KPNA3	O00505	9	3	176	102	91	359	67	18	259	28	62	67	41	19	179
KPNA4	O00629	11	7	406	118	321	464	94	83	148	187	313	168	115	138	170
KPNA6	O60684	11	2	433	142	2	4	25	1	2	5	9	8	2	6	6
KPNB1	Q14974	35	23	1365	3986	2080	2465	949	866	1237	1916	2075	1634	1692	1220	1772
KPRP	Q5T749	7	5	87	435	710	373	357	712	473	278	161	154	179	204	172
KRI1	Q8N9T8-2	7	2	208	20	9	4	2	4	1	11	8	3	3	3	5
KRR1	Q13601	10	4	174	40	22	34	14	8	14	54	90	46	53	43	61
KRT1	P04264	59	39	3417	171230	185923	135943	139222	199867	134998	139129	54959	89551	76171	91452	74955
KRT10	P13645	42	23	2584	77156	87881	64529	52475	84313	54328	49819	23849	30742	25693	30336	31500
KRT13	K7ERE3	20	2	917	162	144	2	0	1	48	0	0	0	0	1	1
KRT14	P02533	43	8	1818	6329	4973	3211	3571	4925	4095	3287	993	1846	1517	1840	1511
KRT16	P08779	38	14	1491	2322	1865	1941	1956	3788	2563	2923	504	1410	1062	1131	718
KRT18	P05783	59	40	2549	21191	24654	26086	11332	9829	12766	19796	18849	18918	21864	20375	20982
KRT19	P08727	54	24	2938	9173	17116	17275	5881	5807	8893	10568	10077	11686	14838	12891	13822
KRT2	P35908	51	31	3047	69953	87489	72293	44880	75138	51591	46266	22355	29798	20176	25614	30827
KRT35	C4AM86	15	4	307	192	12	10	24	26	20	12	14	14	21	14	13
KRT4	F5H8K9	11	4	297	249	499	444	27	29	83	54	44	58	64	48	75
KRT5	P13647	46	21	1896	5067	7427	4733	2808	4792	3871	2897	997	1682	1177	1622	1592
KRT6A	P02538	40	2	1713	409	497	456	150	371	307	237	36	110	99	154	82
KRT7	P08729	49	20	2796	5727	15054	17993	4641	4818	9019	5469	5746	6260	7351	5873	7527
KRT71	Q3SY84	12	2	319	51	1	1	4	3	2	26	10	11	4	4	18
KRT8	P05787	80	20	4137	8589	19562	23627	7650	6978	11321	12913	12684	13610	14103	13732	15752
KRT80	Q6KB66	13	3	202	9	16	16	36	72	40	43	19	30	37	24	29
KRT82	Q9NSB4	12	4	139	472	1466	891	2993	2935	4216	1291	1316	1334	1751	1569	1765
KRT85	P78386	20	2	695	12	25	8	7	9	2	5	10	10	9	6	12
KRT9	P35527	45	10	2269	28295	41394	21817	20346	27627	19335	19338	9051	11453	11610	12663	9999
KTN1	Q86UP2	47	17	1375	527	203	251	228	74	91	334	416	351	304	189	280
LACTB	P83111	14	8	437	933	268	681	141	144	204	202	281	273	275	309	347
LAMA3	Q16787	20	3	87	26	88	11	22	15	41	24	30	36	24	24	27
LAMA5	O15230	65	42	2137	2844	369	1008	1155	922	1252	5332	5374	5506	5167	7531	4824
LAMB1	G3XAI2	25	17	632	303	73	68	130	189	144	2428	1905	1923	236	730	311
LAMB2	P55268	29	16	829	844	201	183	2500	2452	2580	1182	854	858	3653	3749	2742
LAMB3	Q13751	9	2	81	6	7	6	17	9	15	30	6	8	30	1	7
LAMC1	P11047	36	27	1119	2485	1208	1845	1310	1333	1310	5612	4134	4346	4411	6036	4088
LAMC2	Q13753	12	3	95	19	20	35	11	3	6	18	31	22	260	10	24
LAMTOR1	Q6IAA8	4	4	101	26	17	19	87	74	43	74	58	92	90	72	87
LAMTOR2	Q9Y2Q5	3	3	87	24	18	14	20	20	13	16	21	16	19	24	23
LANCL1	E9PHS0	9	4	80	98	34	25	61	45	41	113	176	156	133	128	114

LANCL2	Q9NS86	5	2	105	2	4	1	1	3	1	7	11	7	1	7	3
LAP3	P28838	9	6	312	120	76	145	59	55	73	100	143	132	151	96	120
LARP4B	Q92615	9	2	149	22	12	8	10	8	4	39	38	58	48	45	54
LARP7	Q4G0J3	4	3	114	31	8	4	9	3	3	25	32	14	7	4	12
LARS	F5H698	36	23	929	587	294	600	496	411	549	678	704	782	635	545	686
LAS1L	Q9Y4W2	21	15	699	711	221	303	151	67	101	427	600	363	313	254	366
LASP1	Q14847	16	11	423	1134	316	492	1651	1487	1775	1821	1767	2099	2436	1960	2516
LBR	Q14739	6	3	129	509	25	52	102	13	38	128	180	101	75	57	55
LCP1	P13796	23	12	680	1168	162	220	1475	1566	1840	909	844	981	857	803	963
LDHA	P00338-3	27	7	1053	2560	1663	2686	954	646	848	1026	1002	1027	1567	1192	1714
LDHB	P07195	15	4	521	3857	329	912	552	284	411	332	368	317	379	278	396
LEMD2	Q8NC56	5	4	214	36	20	14	48	46	57	107	104	116	107	102	88
LEMD3	Q9Y2U8	9	4	249	33	39	25	14	6	9	20	44	43	19	35	38
LENG8	E7EQP8	7	2	66	79	7	12	8	4	11	22	24	16	22	11	11
LETM1	O95202	7	3	61	36	17	47	34	51	34	53	37	39	67	53	61
LGALS1	P09382	7	7	294	2192	2462	3054	981	886	1165	752	856	858	991	873	941
LGALS3	P17931	4	3	38	17	34	32	65	51	67	44	73	81	71	65	70
LGALS3BP	Q08380	6	3	106	24	23	23	12	6	16	57	48	89	87	96	93
LGALS8	F6V2D4	8	4	94	67	75	105	158	168	217	306	470	348	401	313	436
LGALS9B	Q3B8N2	5	2	168	63	91	21	7	10	15	62	37	117	73	58	142
LIG1	B4DTU4	5	3	32	558	1734	11	52	2	11	545	9	2	3	5	4
LIG3	P49916	12	7	277	386	102	77	1421	2638	617	850	628	699	1214	999	1559
LIMCH1	Q9UPQ0-4	19	10	484	179	91	54	255	206	128	427	533	379	517	570	462
LIMD1	C9JRJ5	4	2	110	1	4	0	72	38	61	26	28	31	44	26	40
LIMS1	P48059-3	9	5	239	301	861	810	251	228	228	198	207	248	296	206	211
LMAN1	P49257	8	5	153	67	54	112	8	17	34	31	36	53	47	33	49
LMAN2	D6RBV2	5	3	48	1469	1388	2518	586	634	980	877	753	1062	977	1017	1064
LMF2	H0Y9R0	10	4	147	15	8	15	13	6	8	38	57	69	44	29	40
LMNB1	P20700	78	52	3543	8702	12177	14600	1330	1181	3609	7230	8119	4675	4574	3916	5399
LMNB2	J9JID7	69	43	2890	3525	3903	5139	973	897	1306	4505	4439	3324	3355	2805	4473
LOXL2	Q9Y4K0	14	7	301	278	167	147	1376	1373	1728	533	434	575	583	680	519
LPP	Q93052	16	16	226	134	43	83	1878	1563	1535	1273	995	1294	1425	1444	1204
LRCH1	Q9Y2L9-2	9	7	105	45	42	114	137	140	179	137	160	183	160	133	157
LRP1	Q07954	27	12	267	55	44	44	34	17	24	109	120	156	118	107	78
LRPPRC	P42704	39	20	1115	11498	387	331	594	151	291	668	475	314	321	226	287
LRRC16A	Q5VZK9	16	11	236	78	57	94	106	90	74	165	180	186	172	160	170
LRRC47	Q8N1G4	8	2	95	1	1	2	48	50	70	99	102	129	106	95	127
LRRC59	Q96AG4	11	7	228	229	215	245	406	478	705	1141	1569	1402	1251	1227	1319
LRRFIP1	Q32MZ4	8	2	189	13	6	7	1	4	1	0	23	29	0	13	47
LRRFIP2	Q9Y608	16	3	293	67	14	8	126	90	107	80	91	71	72	65	63
LRWD1	Q9UFC0	9	5	207	20	30	12	12	18	17	44	47	43	51	46	41
LSM1	O15116	5	2	43	10	11	8	23	19	10	52	62	62	53	52	48
LSM14A	B4DTG6	3	2	71	33	94	75	27	43	20	60	93	69	57	55	54
LSM14B	Q5TBQ0	2	2	41	27	11	23	37	54	10	31	27	44	67	53	133
LSM2	Q9Y333	7	5	245	103	101	114	187	253	67	276	311	322	366	275	375
LSM4	Q9Y4Z0	7	3	83	23	71	69	11	11	17	47	89	82	61	62	56
LSM6	P62312	5	2	137	7	114	92	56	48	69	90	85	77	110	44	97

LUC7L2	B7Z4Q3	8	4	204	66	31	23	6	2	9	41	47	20	18	16	19
LUC7L3	J3KPP4	11	6	262	131	24	64	49	45	48	136	146	119	57	60	98
LUM	P51884	6	2	137	14700	314	30	2473	70	15	886	104	9	568	26	11
LUZP1	Q86V48	54	38	1389	1064	380	383	4789	4570	5450	3888	3513	3811	4045	4043	4477
LYAR	Q9NX58	6	3	169	22	9	10	7	12	8	37	50	45	30	43	22
LYZ	P61626	4	2	59	69	79	81	53	96	44	42	24	136	27	25	14
MACF1	E9PLY5	47	2	1108	22	53	53	98	79	62	95	140	112	139	119	139
MAD1L1	Q9Y6D9	32	3	723	52	37	17	6	7	21	19	37	30	20	17	32
MADD	Q8WXG6-5	10	3	53	131	287	282	53	56	90	253	257	215	279	188	257
MAEA	B4DVN3	5	3	59	2	6	7	10	6	5	15	19	22	23	15	24
MAGED2	Q5H909	9	2	129	88	46	22	15	5	11	16	19	8	16	10	12
MAGOHB	Q96A72	7	3	295	62	163	180	91	104	196	225	298	209	240	198	237
MAGT1	Q9H0U3	2	2	89	138	21	33	18	7	8	26	20	23	20	13	25
MALT1	Q9UDY8	4	2	30	40	47	38	26	11	14	16	27	28	24	18	26
MAN2A1	Q16706	13	6	244	308	42	90	32	39	46	42	29	49	30	29	35
MAP1B	P46821	41	26	1027	1127	454	698	995	1008	917	2063	1769	2011	1835	1683	2290
MAP1S	B4DH53	7	2	93	5	15	22	27	25	28	49	45	58	53	43	59
MAP3K2	Q9Y2U5	9	2	68	13	17	31	10	10	7	48	55	46	44	37	16
MAP4	E7EVA0	49	8	915	837	1756	1071	710	655	638	1414	1259	1192	1280	1079	1548
MAP4K4	G5E948	23	8	409	100	108	41	88	114	120	182	179	165	205	228	227
MARK2	Q7KZI7-10	12	3	70	42	4	31	14	14	12	77	91	67	64	52	80
MARS	P56192	23	10	577	199	171	291	153	143	173	427	526	550	365	332	453
MAST4	E7EWQ5	16	4	116	58	24	34	154	164	174	219	154	257	257	233	271
MAT2A	B4DN45	6	4	151	104	30	45	22	16	15	25	50	39	21	36	38
MATR3	A8MXP9	40	32	1668	5874	5753	6463	1500	1298	2055	5089	6569	3132	3258	2998	4025
MAVS	Q7Z434	3	3	80	19	0	6	1	0	3	4	5	2	2	2	1
MB21D1	Q8N884	16	11	205	85	32	52	146	133	135	148	149	189	151	159	203
MB21D2	Q8IYB1	7	2	147	10	7	15	10	0	14	3	6	6	1	4	3
MBD3	K7EIE8	5	2	93	345	2	6	51	2	4	16	7	9	9	6	17
MBOAT7	Q96N66	3	3	93	2	4	26	8	9	1	30	57	66	62	55	62
MCM2	P49736	25	19	798	454	531	1202	90	87	247	347	226	354	170	145	260
MCM3	B4DWW4	28	18	1002	1226	856	1039	911	773	450	804	583	560	457	623	551
MCM3AP	O60318	9	4	133	118	52	64	65	99	161	113	63	87	117	65	97
MCM4	J3KPV4	25	15	716	426	464	785	282	320	457	433	392	392	310	370	398
MCM5	P33992	26	12	532	534	225	646	393	380	661	366	332	338	295	275	321
MCM6	Q14566	24	19	635	932	1719	2375	594	658	907	709	796	1317	865	1013	940
MCM7	P33993	37	27	1125	809	798	971	507	487	631	691	673	789	489	561	628
MCMBP	Q9BTE3	5	2	46	13	53	17	8	8	7	11	17	12	13	11	10
MCTS1	Q9ULC4	3	2	86	71	124	120	84	88	83	115	119	116	95	109	104
MDC1	Q14676-3	15	6	161	34	118	101	20	23	44	39	61	30	27	31	27
MDH1	P40925	9	5	192	268	331	930	162	138	178	157	164	182	206	159	198
MDH2	P40926	13	8	391	619	583	772	551	409	554	308	333	311	386	281	300
MDN1	Q9NU22	34	10	365	272	776	129	381	213	911	79	77	64	72	57	66
MECP2	P51608	4	2	124	19	6	26	10	1	0	13	23	2	7	4	0
MED1	Q15648	7	3	76	1996	28	24	252	9	44	87	40	37	67	26	49
MED12	H0Y7P1	12	4	188	17	6	5	1	1	4	7	7	3	5	3	4
MED16	Q9Y2X0	8	5	137	85	33	48	46	21	24	66	67	58	42	28	42

MED17	Q9NVC6	9	2	119	17	3	6	0	1	0	17	18	19	5	3	11
MED23	H7BYY3	14	3	248	5	1	10	0	3	2	9	19	11	6	6	15
MED24	F8W9R9	14	5	322	102	40	15	141	147	9	103	99	143	152	133	159
MED27	Q6P2C8	8	6	72	52	21	25	13	7	29	19	24	24	14	14	39
MED4	Q9NPJ6	7	4	129	14	16	11	10	2	5	20	44	24	12	28	21
MEN1	E7EN32	6	3	131	41	8	11	8	4	19	12	28	5	8	8	10
METAP1	P53582	5	4	95	262	518	394	95	73	82	105	153	99	136	106	84
METTL14	Q9HCE5	5	2	44	19	3	4	0	2	3	8	14	9	8	6	6
METTL3	Q86U44	5	2	35	66	6	11	5	8	4	11	15	15	15	7	14
MFAP1	P55081	8	6	216	81	144	84	11	39	22	85	85	40	50	33	58
MGAT1	P26572	8	5	164	40	7	22	7	9	7	15	20	24	18	16	13
MGST3	Q5VV89	4	3	128	86	16	44	53	38	17	63	69	83	83	65	55
MIA3	Q5JRA6	25	3	292	0	2	8	31	29	28	30	34	23	16	26	11
MICAL2	O94851	12	4	94	12	8	7	11	13	12	26	29	26	23	21	27
MICAL3	Q7RTP6	22	5	167	54	28	31	514	642	972	200	82	171	182	168	120
MIF	P14174	2	2	47	184	296	299	474	546	545	497	481	388	599	514	439
MKI67	P46013	41	18	318	694	1959	1617	577	771	1022	1171	1026	1321	1208	1015	1185
MKI67IP	Q9BYG3	12	3	377	271	98	140	45	14	16	115	174	106	118	90	120
MLEC	F5H1S8	3	2	79	19	13	43	3	1	2	2	4	11	1	1	3
MLF2	Q15773	3	2	53	16	44	93	17	14	17	15	33	19	26	20	23
MLL	E9PQG7	21	5	228	17	16	19	21	20	28	33	49	51	36	36	48
MLLT4	H0Y7R8	15	2	218	9	15	106	4	132	19	44	531	89	6	410	6
MMS19	Q96T76	13	3	226	21	16	20	13	11	11	28	31	28	22	19	22
MOGS	Q13724	18	13	344	1930	915	842	403	375	267	736	738	1013	863	820	924
MON2	Q7Z3U7	15	6	183	182	4751	642	63	5498	3325	263	1288	2493	336	1689	2271
MORC3	Q14149	7	4	136	6	5	11	4	2	5	12	18	7	4	0	5
MORF4L1	H0YNE0	7	2	55	119	420	77	96	141	233	110	125	127	128	126	134
MOV10	Q9HCE1	28	2	636	19	18	23	40	26	19	83	104	108	122	98	122
MPDZ	F5H1U9	16	6	122	73	40	62	60	60	59	155	62	77	51	65	55
MPHOSPH10	O00566	12	8	221	726	57	33	108	59	32	124	126	81	126	59	112
MPHOSPH8	Q99549	7	3	72	127	23	12	12	32	11	22	24	20	22	26	31
MPP5	Q8N3R9	11	4	151	52	26	42	36	28	34	70	124	80	53	32	78
MPRIP	H0Y2S9	38	2	933	8	43	37	34	41	64	43	41	43	45	43	50
MPRIP	Q6WCQ1	43	13	1226	811	69	68	1247	1062	1393	929	914	863	871	871	959
MRC2	Q9UBG0	3	2	51	42	128	173	9	4	5	10	15	18	9	7	6
MRE11A	B3KTC7	15	10	332	223	211	408	80	86	186	207	344	230	155	129	306
MROH1	Q8NDA8	11	2	130	13	23	8	7	8	8	19	17	24	11	13	17
MROH8	Q9H579-2	4	2	44	1	1	2	28	15	32	10	14	12	12	8	12
MRPL1	Q9BYD6	3	2	39	125	19	47	25	13	10	43	58	72	59	45	87
MRPL15	Q9P015	4	3	45	13	6	9	16	15	10	25	30	20	15	12	16
MRPS22	G5E9W7	7	3	139	5	11	9	26	10	9	13	12	10	11	6	12
MRPS27	B4DRT2	5	2	50	1	1	5	27	29	46	38	60	88	84	78	82
MRPS31	Q92665	4	2	55	28	26	6	2	3	6	8	16	13	7	10	10
MRTO4	Q9UKD2	14	12	331	438	494	681	178	158	258	1223	1726	1164	1354	1161	1379
MSH2	P43246	22	14	490	552	221	319	331	332	353	305	356	288	308	307	308

MSH6	P52701	23	11	410	214	38	28	46	39	45	47	69	39	65	34	38
MSN	P26038	68	33	2929	5451	4645	5351	3774	3764	4888	9243	12056	8710	8290	8412	8693
MST4	B4E0Y9	12	2	400	0	3	1	19	11	1	11	17	12	6	14	3
MTA1	E7ESY4	15	4	294	45	20	53	13	8	24	42	55	108	24	22	62
MTA2	O94776	26	14	687	814	318	444	120	98	129	589	723	518	488	367	663
MT-CO2	P00403	3	2	59	222	148	410	14	15	45	20	27	25	29	22	30
MTDH	Q86UE4	8	5	248	129	44	30	40	15	21	100	112	99	75	58	75
MTHFD1	P11586	22	2	641	0	0	51	3	3	5	9	13	18	5	7	7
MTHFD1L	Q6UB35	9	2	130	8	0	6	0	1	1	3	3	2	2	1	0
MTOR	P42345	15	5	162	117	36	15	31	17	10	59	50	56	38	26	27
MTX2	Q8IZ68	3	3	59	531	55	19	94	128	115	86	67	54	94	101	75
MUC16	B5ME49	35	10	181	263	390	580	908	803	935	2026	2382	1844	1771	1498	2190
MVP	Q14764	25	14	651	805	416	619	360	380	454	752	659	1005	892	889	950
MX1	P20591	39	24	1087	1281	768	1251	523	306	489	1679	2150	1738	1568	1612	1403
MX2	P20592	46	28	1539	3485	970	1135	860	734	549	1232	1097	1053	820	725	907
MYADM	Q96S97	4	4	95	73	138	192	517	611	706	375	397	401	336	380	412
MYBBP1A	Q9BQG0	48	2	1465	5	2	6	6	2	10	48	69	33	27	17	34
MYEF2	Q9P2K5	14	5	397	130	88	104	175	162	337	178	209	246	200	205	202
MYH10	F8W6L6	174	2	7416	22	34	47	122	118	178	113	123	148	109	121	133
MYH11	P35749	55	4	1733	251	19	21	84	91	87	86	107	83	97	90	104
MYH14	F2Z2U8	50	6	1048	77	219	143	484	553	557	565	507	486	521	430	555
MYH7	P12883	27	3	198	55	50	50	27	17	24	59	52	53	46	57	57
MYH7B	A7E2Y1	28	3	192	529	46	32	87	25	15	29	43	28	22	53	19
MYH9	P35579	251	154	13045	40231	65220	59768	320896	332203	305759	263969	256400	257718	249990	270972	238406
MYL12A	J3QRS3	16	6	882	5987	14114	13686	15125	15638	19124	9656	12824	10896	13733	13345	12004
MYL6	G8JLA2	17	4	895	343	693	1344	1537	1847	2075	1160	1196	972	1495	1193	1350
MYL9	P24844	13	3	697	40	98	73	170	185	170	245	333	274	250	263	201
MYLK	Q15746	43	31	1148	851	536	447	3739	3831	3549	2708	2748	3008	2833	2953	3088
MYO18A	J3KNX9	47	23	632	267	257	272	764	1018	825	608	667	658	646	743	649
MYO1B	E9PDF6	40	19	1060	481	442	678	846	855	573	1907	2102	2301	1777	1893	2133
MYO1D	J3QRN6	18	4	209	44	20	12	32	20	32	37	45	49	26	35	45
MYO1E	Q12965	16	6	241	90	83	56	107	103	137	210	190	251	221	179	227
MYO5A	G3V394	25	3	186	3	5	6	68	67	96	49	61	34	93	69	81
MYO6	Q9UM54-6	25	13	563	307	253	236	421	517	546	480	475	479	392	388	416
MYO9B	M0R0P8	17	6	115	44	45	144	31	63	39	136	181	174	156	125	265
MYOF	F8W8J4	46	14	962	243	127	131	73	45	50	197	193	201	175	109	175
MYPN	F5GWA6	8	3	108	10	3	4	2	2	2	5	12	1	4	5	3
NAA15	Q9BXJ9	9	3	128	17	50	59	32	28	25	33	39	42	33	34	37
NAA38	O95777	4	2	212	34	336	242	20	12	42	11	29	11	11	7	5
NACA	E9PAV3	12	6	354	5970	5918	6573	1653	1694	2621	1356	997	1363	1572	1488	1468
NACC1	Q96RE7	7	4	136	13	8	10	64	2	2	10	7	9	23	2	5
NAGK	Q9UJ70-2	11	2	183	0	6	5	46	54	71	70	67	103	109	78	67
NAMPT	P43490	13	6	237	65	107	215	80	73	134	205	280	273	291	203	364
NANS	Q9NR45	7	2	81	12	3	16	37	28	48	73	79	91	99	73	91
NAP1L1	H0YIV4	7	2	322	63	63	94	189	174	109	243	232	198	199	159	214
NAPA	M0R2M1	6	3	184	19	18	41	11	5	8	15	26	21	11	24	16
NAPG	B4DFC9	5	2	40	16	63	83	515	373	52	203	180	184	187	252	195

NARS	O43776	13	7	191	124	65	76	112	105	156	192	188	216	151	172	197
NASP	P49321	7	5	119	255	93	183	18	17	31	68	63	41	74	64	83
NAT10	Q9H0A0	36	22	1029	1313	327	583	369	228	358	539	661	477	478	350	576
NBN	O60934	18	7	362	271	9	20	54	58	58	63	70	39	38	38	39
NCAPD2	Q15021	13	6	231	37	87	54	35	19	62	11	14	24	11	10	23
NCAPG	Q9BPX3	12	3	272	1	0	18	15	19	19	16	17	9	12	12	11
NCAPG2	H0Y6U5	8	3	52	1616	34	54	593	395	528	587	589	678	745	556	781
NCBP1	Q09161	18	9	601	344	127	181	102	92	139	238	248	160	173	152	160
NCBP2	P52298	4	2	107	57	14	41	13	15	23	40	56	56	44	39	49
NCKAP1	Q9Y2A7	17	9	340	37	56	63	178	180	203	248	218	301	233	195	248
NCKIPSD	Q9NZQ3	6	5	112	51	132	100	396	588	438	320	206	289	448	391	541
NCL	P19338	42	27	1718	9520	10824	12896	3030	1837	3225	6307	7653	4302	4340	4024	4464
NCOA5	Q9HCD5	16	10	198	119	140	195	348	202	291	233	319	238	215	257	253
NCOR1	O75376-2	20	6	218	38	42	32	80	70	72	77	74	60	81	77	74
NDUFA10	E7ESZ7	7	3	48	15	45	18	10	13	17	12	24	16	12	10	20
NDUFA5	F8WAS3	2	2	43	0	20	18	19	8	23	26	24	35	38	22	51
NDUFA9	Q16795	8	5	86	724	41	47	134	18	29	37	60	51	46	44	43
NDUFS1	P28331	14	10	293	26	28	33	46	44	46	56	78	74	79	48	78
NDUFS2	O75306	6	2	102	9	8	18	74	96	95	38	45	55	50	51	42
NDUFS3	O75489	7	4	142	77	1374	1176	1192	1566	1405	260	739	1208	1359	1136	1399
NDUFV1	P49821	8	4	152	46	148	32	11	10	16	8	10	10	15	13	10
NDUFV2	E7EPT4	3	2	33	37	34	36	4	7	19	6	7	9	7	4	9
NEB	F8WCP0	59	6	239	53287	1394	1291	6108	535	443	1840	536	602	1308	574	640
NECAP2	Q9NVZ3	4	3	75	38	61	30	31	18	21	154	124	137	140	86	112
NEFL	P07196	27	10	696	269	211	306	203	136	237	282	277	284	286	328	287
NEK7	Q8TDX7	4	2	44	0	6	5	13	15	15	17	24	16	15	32	11
NEK9	Q8TD19	4	2	46	2	8	19	17	7	5	94	80	67	68	59	80
NELFE	B4DUN1	6	3	157	20	28	3	1	0	10	14	7	3	6	2	6
NEURL4	I3L100	9	3	72	96	99	218	398	293	254	552	507	653	618	554	539
NEXN	Q0ZGT2	24	8	426	224	28	25	73	70	82	67	76	78	62	54	46
NF1	P21359	11	4	61	2394	4220	2255	890	1301	1925	547	424	680	927	1581	1166
NF2	P35240-2	11	4	156	94	183	91	119	134	180	85	80	80	92	86	91
NFIC	K7EMU1	4	2	69	32	33	8	7	3	19	10	19	2	3	1	2
NFKB1	P19838	9	3	223	181	81	65	198	146	40	231	245	274	230	231	316
NFKB2	Q00653	20	11	337	64	227	240	255	250	406	434	514	600	440	376	515
NHP2L1	B1AHD1	9	6	323	128	107	239	52	52	69	165	235	147	195	157	159
NID1	P14543	10	7	161	42	43	83	408	473	622	288	209	309	304	349	301
NIN	C9J066	24	7	196	259	505	628	417	502	687	1125	782	934	1179	979	1311
NIP7	Q9Y221	6	4	97	49	73	161	86	77	98	169	303	219	151	181	194
NIPBL	Q6KC79	26	8	319	137	509	121	700	681	536	476	335	503	561	492	606
NKAP	Q8N5F7	5	2	45	0	7	3	13	13	5	21	13	32	25	14	35
NKRF	G3V1N1	19	9	317	231	67	103	59	71	25	87	141	62	69	49	73
NME1-NME2	Q32Q12	13	9	371	1235	1872	2042	587	583	746	431	557	601	613	546	620
NME7	Q9Y5B8-2	4	2	30	5	7	12	6	12	22	31	59	46	42	28	28
NMNAT1	Q9HAN9	6	4	107	24	18	26	22	16	28	38	50	43	35	25	34
NMT1	P30419	3	3	29	50	43	13	29	26	26	44	46	54	44	40	55

NNMT	P40261	5	3	188	34	157	437	137	104	116	263	286	287	304	226	242
NO66	Q9H6W3	8	5	101	187	90	49	60	37	68	90	120	103	122	87	104
NOC2L	Q9Y3T9	13	9	376	439	220	100	286	49	59	611	427	176	215	164	244
NOC3L	A6NJZ9	23	12	503	339	130	160	111	104	130	268	284	189	208	177	182
NOC4L	Q9BVI4	16	6	394	14	18	39	11	8	18	43	53	33	32	30	53
NOL10	F5H6G7	11	6	201	34	52	61	22	8	16	7	33	11	18	1	10
NOL11	Q9H8H0	14	6	340	62	20	42	12	10	13	112	142	78	75	51	115
NOL6	Q9H6R4	23	17	685	578	593	806	144	235	671	217	407	297	195	239	528
NOL7	Q9UMY1	6	3	127	213	46	13	16	17	10	39	54	28	21	12	31
NOL8	Q76FK4	10	4	152	25	10	20	11	9	12	27	20	19	20	11	15
NOL9	Q5SY16	14	10	244	471	117	98	119	126	98	247	299	223	171	160	237
NOLC1	Q14978	13	6	300	438	118	145	44	22	97	160	301	86	101	96	143
NOMO1	Q15155	6	2	48	6	1	0	2	2	3	8	4	4	13	9	4
NONO	H7C367	29	2	754	32	3	61	32	35	24	58	45	61	56	51	66
NONO	Q15233	44	15	1542	1495	1398	1496	473	466	542	4257	5293	3440	3441	2742	4731
NOP14	P78316	17	7	514	371	80	122	31	19	37	45	63	29	18	19	16
NOP16	Q9Y3C1	6	3	152	12	5	5	4	2	1	6	14	2	6	4	6
NOP2	P46087	24	17	744	620	327	459	112	195	334	559	745	491	501	394	660
NOP56	O00567	28	11	1048	378	264	212	87	61	99	485	594	384	398	275	551
NOP58	Q9Y2X3	26	21	1216	1105	1098	1165	403	331	435	1433	1742	968	1074	1333	1200
NOP9	Q86U38	8	4	121	29	27	53	20	5	8	22	36	33	11	10	19
NOSIP	Q9Y314	6	2	103	129	4	7	18	12	5	20	24	28	20	17	26
NPEPPS	P55786	15	5	373	51	34	75	80	59	97	89	107	123	94	75	131
NPHS1	O60500	11	9	211	77	25	48	58	51	61	59	101	56	71	45	54
NPM1	P06748	16	12	963	10282	30560	35440	5332	3548	12591	11559	18457	11776	8230	10910	9961
NPM3	O75607	3	2	54	46	162	187	25	34	59	99	131	101	68	55	69
NR3C1	P04150-5	4	2	63	2	5	2	9	13	3	17	15	11	12	8	10
NSA2	O95478	5	3	88	25	5	9	31	24	58	15	21	26	26	14	50
NSF	P46459	17	6	146	248	103	105	65	28	45	82	68	71	74	51	66
NSFL1C	Q9UNZ2-4	7	5	56	127	13	171	22	14	27	28	22	45	30	19	46
NSMCE4A	Q9NXX6	4	2	101	3	9	16	0	2	0	5	11	6	2	4	10
NSUN2	Q08J23	22	15	459	561	204	290	176	178	221	433	440	398	333	312	490
NSUN5	Q96P11-2	8	4	113	18	12	42	58	39	57	96	82	90	117	71	109
NT5C2	B7Z382	5	5	42	47	30	79	68	31	38	62	64	69	65	42	68
NT5C3	Q9H0P0	3	2	31	13	23	13	4	5	5	6	9	6	8	5	5
NT5E	P21589	33	16	1220	1345	3433	3726	3823	4250	5964	6142	6961	7184	6994	8396	7920
NTPCR	Q5TDF0	2	2	62	14	15	7	62	118	98	143	130	197	200	219	230
NUCB1	H7BZI1	5	3	128	1977	29	5	292	6	5	55	5	2	62	3	1
NUDC	Q9Y266	9	4	253	13	3	24	33	23	22	79	59	84	75	67	91
NUDT16L1	K7EIN2	4	3	73	9	31	30	33	29	70	49	63	60	65	58	81
NUDT21	O43809	16	10	454	1692	1861	2668	497	671	839	1247	1406	1373	1496	1341	1608
NUFIP2	Q7Z417	8	4	84	127	15	18	20	17	13	84	88	90	93	65	107
NUMB	P49757	6	5	101	58	9	13	32	35	25	69	77	86	66	95	72
NUP107	P57740	28	13	840	227	137	172	247	305	323	545	458	456	486	384	504
NUP133	Q8WUM0	36	23	938	1000	665	900	343	323	365	629	890	621	454	441	511
NUP153	P49790	27	17	817	189	74	206	127	134	166	394	381	269	245	148	251
NUP155	E9PF10	44	32	1388	1379	461	678	334	250	288	720	758	637	557	452	709

NUP160	Q12769	34	24	1040	591	321	371	208	164	188	455	564	434	437	555	425
NUP188	Q5SRE5	26	19	574	252	303	426	222	121	401	197	229	277	194	180	178
NUP205	Q92621	48	31	1412	762	426	637	230	217	324	828	801	741	598	504	676
NUP210	Q8TEM1	39	27	1206	665	536	953	884	661	720	1786	1879	2051	1295	1078	1594
NUP35	B4DYB4	10	4	332	81	44	36	11	9	13	146	173	130	115	80	137
NUP37	Q8NFH4	7	5	157	892	743	546	302	243	267	291	241	251	253	224	259
NUP43	Q8NFH3	6	5	202	153	164	269	95	97	114	192	263	215	178	149	228
NUP50	Q9UKX7-2	6	3	113	9	162	324	7	6	7	35	30	30	13	10	21
NUP54	B4DT35	16	12	580	238	106	187	96	81	124	318	347	320	255	200	313
NUP62	P37198	4	3	161	42	46	13	7	13	11	48	43	45	32	26	53
NUP85	Q9BW27	19	11	828	447	245	164	147	107	111	350	425	351	356	254	396
NUP88	Q99567	21	12	389	70	258	365	50	73	113	199	208	162	153	151	161
NUP98	P52948	40	25	1384	1288	515	759	468	406	332	804	944	723	754	762	794
NUPL1	Q5JRG1	10	6	220	75	82	73	15	18	28	53	92	34	68	39	43
NUTF2	P61970	3	2	95	14	15	18	1	0	0	4	14	6	6	1	4
NVL	O15381	13	8	398	18	25	14	5	8	12	25	35	33	16	13	35
NXF1	Q9UBU9	15	7	411	73	50	39	46	61	69	109	112	70	58	62	73
NXT1	Q9UKK6	4	3	97	68	22	28	33	36	24	46	47	40	42	45	39
OAS2	P29728	12	7	399	96	71	48	17	17	16	46	54	47	52	44	59
OAS3	Q9Y6K5	22	9	566	108	121	213	311	425	357	739	781	807	816	903	787
OASL	Q15646	15	11	399	134	76	78	37	24	35	106	175	146	112	103	136
OCIAD2	Q56VL3	5	3	178	99	95	118	48	57	69	75	82	76	127	102	52
OCRL	Q01968	9	2	86	15	4	8	4	3	4	12	13	22	4	10	19
OGDH	F5H801	13	5	204	194	203	148	36	27	33	59	46	58	72	60	97
OGT	O15294	19	10	395	327	103	135	137	129	129	140	156	161	123	140	153
OLA1	J3KQ32	10	3	154	45	59	107	66	43	77	53	50	63	57	55	66
OPTN	Q96CV9-3	11	2	153	13	11	19	49	38	38	52	58	60	65	47	53
ORC1	Q13415	6	2	58	7	10	0	2	0	0	4	5	3	4	2	5
ORC3	Q9UBD5	10	2	93	19	16	38	2	14	9	14	25	19	11	10	10
ORC5	O43913	7	2	106	19	9	17	4	5	8	11	12	9	10	11	7
OSBPL3	Q9H4L5	8	5	146	934	1296	1376	1882	1607	2034	2189	1745	2058	2306	2082	2141
OSBPL9	B1AKJ6	5	2	33	3	1	6	11	14	12	24	22	28	23	23	36
OTUB1	Q96FW1	5	3	94	43	39	65	16	16	27	27	27	32	31	26	31
P4HA1	P13674	10	4	259	42	21	55	11	14	11	20	40	37	29	24	35
P4HA2	O15460	5	4	112	70	104	154	43	61	89	77	107	96	88	65	84
P4HB	P07237	26	3	744	495	169	826	142	134	178	207	231	283	249	198	263
PA2G4	F8VTY8	8	4	98	150	230	359	70	75	87	131	113	150	138	109	135
PACSIN3	Q9UKS6	4	2	94	16	4	12	4	5	3	8	13	9	3	3	4
PAF1	M0QX35	11	6	267	64	87	56	22	17	40	72	105	78	46	48	69
PAFAH1B1	P43034	9	5	135	121	42	40	25	34	27	68	72	65	63	67	51
PAICS	P22234	18	12	555	954	516	808	1157	1103	973	1281	1148	1170	1173	1184	1051
PAK1IP1	Q9NWT1	6	3	126	76	44	111	12	11	16	11	23	10	12	11	15
PAK2	Q13177	8	2	139	17	12	19	20	13	14	32	34	21	28	39	37
PALLD	Q8WX93	32	23	915	2451	886	923	3429	3185	3851	3903	3945	4148	4093	3966	4039
PAN2	Q504Q3	7	2	51	687	228	1057	30	18	20	27	31	35	24	25	33
PANK4	Q9NVE7	7	2	31	30	10	6	21	15	15	47	45	51	38	29	41
PAPD5	E9PC61	6	3	92	410	250	339	271	260	126	955	959	684	1077	692	47

PAPSS1	O43252	13	7	217	43	30	80	28	19	21	101	119	111	73	50	99
PAPSS2	E7ER89	14	9	341	291	137	243	85	107	164	243	261	245	218	228	339
PARN	B4DSB0	5	3	75	8	4	8	13	11	13	10	9	11	8	8	12
PARP1	P09874	41	26	1092	904	792	963	630	553	740	2614	2613	2160	2126	1786	2476
PARP14	Q460N5	26	12	464	157	66	72	187	83	231	154	191	193	170	141	188
PARP4	Q9UKK3	13	6	131	19	21	16	21	8	24	90	115	127	137	188	165
PARP9	Q8IXQ6-2	23	10	476	104	128	123	74	53	68	169	205	197	188	161	196
PARVA	J3KNQ4	11	6	411	47	52	133	857	691	643	535	435	627	800	685	681
PATL1	Q86TB9	8	4	66	214	406	87	105	95	116	77	95	95	97	79	91
PAWR	Q96IZ0	6	4	226	135	20	77	338	279	472	604	574	507	397	364	650
PAXBP1	Q9Y5B6	14	7	196	8441	133	38	1128	45	66	257	51	39	230	43	43
PAXIP1	Q6ZW49-1	9	3	82	115	24	34	25	27	18	78	44	52	36	47	57
PBRM1	Q86U86-3	25	7	226	23	44	28	22	27	30	44	54	36	27	24	40
PCBP1	Q15365	16	6	547	1018	2135	2833	496	569	1491	964	1242	920	980	908	1024
PCID2	Q5JVF3-4	7	5	122	76	49	19	204	193	153	304	289	254	328	265	232
PCLO	Q9Y6V0-5	30	4	198	61	104	196	156	157	198	226	168	343	245	242	266
PCM1	E7ETA6	18	7	214	231	146	116	291	352	245	252	246	236	282	257	189
PCMT1	J3KP72	9	5	195	152	71	64	79	47	34	59	76	61	64	39	51
PCNA	P12004	17	14	574	1738	1121	1968	316	239	437	341	507	376	357	242	417
PDCD10	Q9BUL8	6	2	166	28	31	26	122	99	91	115	113	122	145	130	126
PDCD11	Q14690	38	21	818	385	501	1100	147	214	254	355	451	269	300	222	371
PDCD5	K7EL31	3	2	136	2	0	11	25	16	30	39	38	57	60	38	47
PDCD6IP	Q8WUM4	27	19	547	394	353	477	442	400	356	637	575	683	604	548	622
PDE4DIP	Q5VU43	28	7	372	132	45	66	86	147	10	82	153	95	187	163	77
PDIA3	G5EA52	12	6	282	924	698	1146	84	76	135	128	139	155	129	87	136
PDIA4	P13667	11	6	343	969	491	551	205	257	355	368	158	248	240	160	249
PDIA6	B5MCQ5	8	5	294	419	140	507	34	33	80	103	122	138	134	98	121
PDLIM1	O00151	18	12	465	2339	898	802	4025	3166	3691	3825	3733	4384	5245	4366	4976
PDLIM2	J3KNH4	7	5	57	15	3	10	73	90	66	34	22	33	32	18	24
PDLIM5	Q96HC4	19	15	506	5833	125	190	1405	835	917	1126	1101	1225	1169	1127	1155
PDLIM7	Q9NR12	19	17	558	1110	674	624	7722	6508	7108	6321	6126	6931	8031	6957	7719
PDPK1	C9JWR9	4	2	31	3	8	1	2	3	1	2	7	7	2	3	6
PDS5A	Q29RF7	35	20	624	718	162	144	169	135	215	259	325	221	196	183	240
PDS5B	Q9NTI5	36	17	747	344	136	229	93	175	158	264	359	273	219	214	305
PDXDC1	H3BND4	8	4	89	294	145	210	57	57	74	109	143	116	113	104	122
PEAK1	Q9H792	15	8	178	119	40	125	275	168	173	295	297	234	276	192	238
PEBP1	P30086	3	2	54	48	11	14	10	3	4	23	63	35	27	18	10
PES1	B5MCF9	18	9	519	166	43	38	25	23	26	130	173	107	95	64	113
PEX11B	O96011	2	2	24	25	5	20	12	12	13	16	55	51	35	35	40
PFDN2	Q9UHV9	4	2	102	5	14	65	33	29	53	76	79	77	61	70	91
PFDN5	H3BPF6	7	2	104	22	37	42	10	4	12	26	31	27	30	22	25
PFKFB4	B7Z5C3	4	2	66	41	73	51	15	25	8	52	31	26	21	49	74
PFKL	P17858	18	7	295	193	41	85	135	117	140	187	206	189	188	157	190
PFN1	P07737	9	6	378	904	933	1479	247	255	374	267	386	318	279	249	233
PGAM1	P18669	12	5	350	525	329	483	602	502	569	505	569	533	588	520	640
PGAM5	Q96HS1	12	5	215	136	74	136	303	296	336	366	348	376	420	435	407
PGD	B4DQJ8	7	4	179	79	34	112	39	24	58	29	45	50	39	29	32

PGK1	P00558	21	10	559	574	537	849	586	620	847	1111	1297	1220	1287	1065	1362
PGK2	P07205	6	2	57	545	266	0	53	3	13	15	6	7	10	4	6
PGM1	P36871	10	5	142	210	106	221	63	64	84	81	95	149	94	91	155
PHB	P35232	11	8	564	710	798	1540	383	422	667	400	449	565	619	586	620
PHB2	J3KPX7	16	8	414	268	768	1165	331	435	534	350	497	620	573	611	722
PHC2	B3KPJ4	7	2	59	20	35	39	26	14	45	36	38	103	35	59	57
PHF3	Q92576-2	17	5	138	167	59	50	201	110	148	229	178	225	212	247	212
PHF5A	Q7RTV0	5	4	201	86	195	92	21	25	54	73	152	89	91	70	60
PHF6	B4E0G4	6	3	73	22	8	7	20	9	13	29	30	24	29	15	24
PHGDH	O43175	8	3	377	85	126	238	65	60	63	38	35	44	36	31	40
PHIP	Q8WWQ0	9	6	46	229	490	382	2720	2884	4983	1442	1356	1576	1434	1067	1642
PHLDB1	Q86UU1	29	2	558	49	13	9	241	152	178	131	90	107	130	133	123
PHLDB2	Q86SQ0	34	10	532	1019	137	132	1230	1149	1304	456	411	440	425	441	459
PHRF1	E9PJ24	7	2	44	9	18	11	4	6	7	43	69	37	48	32	21
PIAS2	Q2TA77	5	2	41	2	66	3	1	0	49	0	2	6	0	1	27
PICALM	Q13492	19	2	655	7	32	2	1	51	7	10	69	67	36	67	51
PIEZO2	Q9H5I5	11	3	81	38	21	32	125	128	123	78	65	101	82	85	109
PIK3C2A	O00443	18	9	199	692	951	710	394	667	441	306	180	300	219	237	309
PIK3C3	A8MYT4	5	2	41	5	9	15	62	104	84	89	70	87	166	113	130
PIK3R4	Q99570	6	3	91	31	38	24	91	142	132	52	46	59	48	46	35
PIKFYVE	Q9Y2I7	18	2	98	930	3448	2733	839	684	1229	3157	2946	3014	4042	2842	3109
PIN1	K7EN45	6	2	53	28	42	30	55	37	8	46	63	60	44	51	44
PIN4	Q9Y237-2	5	2	105	19	2	3	223	189	290	78	77	99	123	134	103
PION	A4D1B5-2	4	2	37	201	546	604	2	4	4	4	2	1	2	3	2
PIP	P12273	5	3	91	59	55	35	82	226	82	89	43	125	53	60	26
PKHD1	P08F94	26	10	157	379	453	493	661	614	314	786	662	868	782	834	889
PKLR	P30613	10	2	128	662	470	139	214	101	181	38	44	39	47	62	51
PKM	P14618	53	38	2114	8516	11636	17444	5801	5413	6069	9970	11348	10427	9405	9324	8840
PLAA	H0YBW4	5	2	82	50	873	676	1090	1455	1840	1314	803	1135	1463	1465	1129
PLAT	P00750	8	8	214	118	663	552	835	1050	1260	280	222	253	240	333	240
PLAU	E7ESM2	3	2	78	13	1	15	209	204	268	48	42	45	47	43	55
PLCB4	E2QRH8	14	3	66	42	8	5	16	9	14	22	26	23	20	12	23
PLEC	Q15149	389	2	17555	9	20	23	12	14	27	31	34	32	44	41	39
PLEC	Q15149-4	385	2	17616	357	86	136	1130	1135	935	919	758	817	983	788	904
PLEKHA5	Q9HAU0-6	13	4	118	38	20	26	26	15	21	38	43	45	35	33	34
PLEKHA6	Q5VTI5	9	3	80	3	3	3	35	47	37	27	46	67	30	50	43
PLEKHG3	A1L390	6	2	56	61	7	5	43	60	77	65	65	63	60	49	65
PLEKHH1	Q9ULM0	12	3	71	9	11	23	168	131	172	147	138	148	168	148	144
PLEKHH2	Q8IVE3	11	4	54	277	162	193	251	401	401	292	180	183	157	179	220
PLIN3	K7EQF4	10	2	348	22	89	122	83	74	69	110	107	134	127	122	131
PLOD1	B4DR87	10	7	265	47	40	38	78	73	47	98	133	157	115	110	176
PLOD2	E7ETU9	7	3	122	32	71	78	19	29	33	40	46	54	62	49	64
PLOD3	O60568	12	5	163	299	39	35	32	11	8	26	27	28	18	37	19
PLRG1	A8MW61	18	10	415	990	195	273	137	71	62	326	420	292	248	332	277
PLS1	Q14651	12	5	241	22	19	12	130	109	130	95	151	85	81	78	92
PLSCR1	B4DTE8	4	4	88	261	98	514	104	78	75	2054	122	222	151	114	134
PNN	Q9H307	23	16	668	777	564	459	215	185	213	548	573	509	444	354	684

PNO1	Q9NRX1	11	2	255	0	0	3	9	12	17	8	19	16	14	10	7
PNP	P00491	7	2	178	27	28	26	42	36	45	34	32	43	35	25	41
PNPLA6	F5H5K9	10	2	92	18	25	8	0	1	5	3	2	0	3	0	3
POGLUT1	Q8NBL1	7	4	92	9908	15	12	1350	92	144	267	75	81	180	87	97
POGZ	B7ZBY5	12	7	179	573	81	105	113	28	59	91	134	77	57	56	73
POLA1	A6NMQ1	11	3	83	184	346	185	120	173	109	100	62	74	58	53	77
POLD1	M0R2B7	14	4	280	15	20	20	18	9	17	25	25	28	16	28	18
POLD2	F8W8R3	7	5	174	57	116	159	77	84	106	65	54	73	50	59	67
POLDIP3	Q9BY77	14	3	333	29	17	18	10	9	7	160	197	124	136	85	189
POLR1A	O95602	30	16	388	529	945	792	530	456	627	1785	1707	1923	2442	2100	2059
POLR1C	H0Y723	9	2	95	9	19	10	11	11	15	27	22	22	32	24	32
POLR1C	O15160	9	2	217	12	14	25	18	19	26	46	49	47	48	33	42
POLR1E	Q9GZS1	4	3	76	30	12	9	3	2	0	12	17	15	13	8	15
POLR2A	P24928	43	21	689	609	206	131	81	60	86	182	329	166	195	171	156
POLR2B	C9J2Y9	30	2	591	58	48	14	27	6	22	42	75	45	35	12	43
POLR2C	P19387	6	5	196	103	44	108	29	9	24	70	150	43	64	65	32
POLR2E	P19388	6	3	274	280	33	57	57	23	9	75	109	57	66	45	53
POLR2G	P62487	4	4	80	314	14	5	24	0	6	18	25	18	14	6	17
POM121	Q96HA1	8	2	99	11	1	5	25	34	16	40	45	38	39	34	23
POP1	Q99575	19	13	364	519	385	478	88	54	80	200	169	237	189	146	190
POP7	C9JYM0	3	3	51	43	7	9	34	25	36	56	65	61	53	48	54
PPA1	Q15181	5	3	106	71	32	73	49	36	24	89	110	97	87	74	89
PPA2	Q9H2U2-2	8	2	99	26	34	55	133	147	192	122	133	145	150	136	175
PPAN	Q9NQ55	10	6	125	113	40	83	104	57	28	99	174	132	133	125	128
PPFIA1	Q13136	30	14	704	162	264	239	3015	2498	2942	1397	1053	1276	1502	1527	1534
PPFIA2	B7Z663	19	2	301	5	2	6	35	25	15	37	43	50	67	57	59
PPFIBP1	Q86W92	40	4	1091	35	158	80	381	342	300	344	373	371	308	289	298
PPHLN1	Q8NEY8	8	4	95	97	28	7	207	243	5	65	74	12	24	67	22
PPIA	P62937	20	12	796	1977	1951	2670	2842	2743	3104	2914	3243	3219	4072	2994	3477
PPIB	P23284	16	9	465	785	918	1316	1493	1535	1885	1425	1633	1658	2361	2103	1674
PPIE	Q9UNP9-2	4	2	63	13	7	8	1	11	4	7	14	5	9	11	3
PPIG	Q13427	8	2	155	41	32	110	3	181	7	34	69	37	25	46	15
PPIH	C9JQD4	10	5	227	71	137	162	270	239	305	224	259	219	239	201	264
PPIL1	Q9Y3C6	4	3	85	229	106	332	126	109	151	225	249	185	229	164	243
PPIL4	Q8WUA2	4	3	56	26	21	20	11	3	5	16	17	8	16	4	11
PPM1G	O15355	6	3	162	2224	66	41	282	8	65	55	44	33	25	20	270
PPME1	J3QT22	6	2	154	89	62	76	66	62	65	56	74	70	65	52	85
PPP1CA	P62136	19	3	871	247	432	440	744	774	1038	705	786	823	1060	753	1158
PPP1CB	P62140	18	3	800	7	15	67	333	309	383	278	343	379	468	381	256
PPP1R12A	O14974	38	7	1109	386	105	116	585	607	566	906	764	697	691	670	720
PPP1R12C	B4DME2	10	2	117	168	10	5	52	54	44	22	41	47	37	29	46
PPP1R13L	Q8WUF5	10	7	157	79	67	40	153	208	149	378	315	314	341	235	312
PPP1R18	Q6NYC8	13	8	242	729	104	172	1204	1218	1580	1149	909	1008	1179	1001	1300
PPP1R7	C9J177	4	3	53	230	276	738	225	316	452	409	315	505	394	399	470
PPP2R1A	P30153	25	8	849	509	272	234	655	395	374	496	581	580	466	521	486
PPP2R2A	P63151	6	5	131	41	37	90	99	88	104	86	104	123	73	70	104
PPP2R3A	Q06190	8	2	89	19	11	8	32	20	25	24	21	26	25	23	18

PPP2R5D	Q14738-3	8	4	122	9	7	8	48	50	71	71	75	97	66	65	96
PPP4C	H3BTA2	6	2	109	5	3	5	4	3	7	9	12	13	15	10	15
PPWD1	F5H7P7	7	2	112	20	4	9	3	0	4	4	8	2	3	1	2
PRC1	F8W9B5	11	2	118	20	50	48	400	572	677	218	227	339	341	369	369
PRDX1	Q06830	20	14	771	2573	4528	5486	3331	2937	3753	3923	4312	4399	5076	4373	4594
PRDX2	P32119	9	3	266	77	163	251	172	169	99	181	190	250	255	240	279
PRDX3	E9PH29	3	3	135	71	62	210	23	21	61	27	21	28	24	15	31
PRDX4	H7C3T4	8	4	222	104	122	142	26	26	34	25	27	18	29	22	28
PRDX5	P30044	6	3	143	39	32	75	89	78	110	80	60	76	58	96	133
PRDX6	P30041	13	7	318	951	393	657	512	283	375	463	643	600	582	485	480
PRKAA1	Q13131	8	4	137	35	61	39	27	20	34	32	46	29	36	26	34
PRKACA	P17612	3	2	62	8	8	19	86	114	125	67	63	92	89	79	118
PRKAG1	Q8N7V9	3	2	51	27	33	45	58	66	75	68	51	74	65	77	82
PRKAR1A	K7ER48	8	3	94	23	19	20	56	32	10	65	61	77	55	33	58
PRKAR2A	Q9BUB1	16	7	369	146	403	461	156	167	144	341	386	374	363	296	401
PRKCA	P17252	8	3	62	13	6	10	7	9	12	57	66	46	27	36	14
PRKCG	F5H5C4	6	2	47	69	131	275	61	33	65	132	108	157	176	177	173
PRKDC	P78527	208	3	7076	118	107	94	29	30	30	127	99	97	84	87	103
PRMT1	H7C2I1	18	6	840	291	418	953	540	504	492	715	1093	972	987	912	939
PRMT5	O14744	14	6	276	94	123	234	138	137	193	259	241	248	291	239	345
PRPF19	Q9UMS4	21	11	697	2294	3237	3889	684	628	982	2243	2659	1785	1586	1639	1580
PRPF3	E7EVD1	15	7	367	222	261	553	240	202	278	396	328	349	358	314	390
PRPF31	Q8WWY3	14	10	209	348	292	374	83	81	155	295	493	239	189	224	321
PRPF38A	Q8NAV1	8	7	161	76	63	58	139	143	152	188	185	148	147	157	172
PRPF4	O43172	18	16	407	316	296	403	197	158	215	513	650	475	489	393	567
PRPF40A	O75400	22	14	516	248	378	457	107	112	182	342	342	277	161	209	222
PRPF4B	Q13523	22	9	368	68	86	100	26	33	64	56	82	62	50	30	59
PRPF6	O94906	37	22	1032	1188	479	674	1581	1552	2008	1718	1702	1594	1734	1559	1706
PRPF8	Q6P2Q9	109	80	3180	7385	2409	3351	1183	1108	1149	3819	4915	3241	3128	2762	3475
PRPS1	B1ALA9	5	2	62	9	14	30	38	34	83	27	32	42	38	24	33
PRPSAP1	Q14558	7	2	180	19	16	56	62	71	49	23	14	21	20	27	23
PRRC2C	Q9Y520	18	4	128	144	158	419	226	214	324	520	612	498	648	509	508
PRSS23	O95084	5	3	83	37	20	7	180	237	189	56	66	82	90	99	79
PSAP	C9JIZ6	4	2	27	9	4	2	7	5	30	45	11	45	9	5	52
PSAT1	Q9Y617	7	2	161	53	69	70	156	131	155	38	39	48	51	32	44
PSD3	J3KQK0	10	6	165	83	19	17	360	373	320	288	168	211	223	277	254
PSIP1	O75475	11	4	201	570	596	576	434	649	667	522	405	327	290	354	324
PSMA1	P25786	9	4	186	131	242	284	195	202	225	248	298	292	397	259	259
PSMA2	P25787	12	9	208	300	352	581	104	95	137	163	284	326	217	156	252
PSMA3	P25788	12	3	376	362	86	54	55	64	49	41	35	47	50	40	38
PSMA5	P28066	10	8	333	839	492	1075	219	236	301	361	411	457	490	367	507
PSMA6	G3V5Z7	9	6	321	255	590	852	117	172	356	141	229	201	161	148	173
PSMB1	P20618	12	7	267	997	356	367	215	164	218	247	342	276	293	240	274
PSMB2	P49721	6	2	73	30	68	115	65	72	49	90	129	115	100	124	94
PSMB3	P49720	8	7	327	76	245	428	97	97	128	248	199	225	175	162	203
PSMB4	P28070	7	4	204	109	159	287	125	174	90	115	181	158	119	121	116
PSMB5	P28074	8	4	341	43	73	178	47	58	55	45	63	66	67	55	77

PSMB6	P28072	6	4	160	143	224	289	36	59	44	37	85	84	72	58	77
PSMB7	Q99436	6	3	72	137	172	62	272	95	537	58	57	61	64	43	54
PSMB8	P28062	5	3	180	79	83	86	68	57	73	68	84	82	80	51	67
PSMB9	B0V0T3	4	2	142	373	77	142	87	57	64	52	57	86	52	67	59
PSMC1	B4DR63	9	5	260	216	223	366	84	80	120	165	161	187	159	142	165
PSMC2	P35998	19	4	406	540	168	79	154	35	41	86	52	51	111	44	44
PSMC3	E9PM69	20	6	460	309	122	262	60	62	100	106	122	129	103	81	120
PSMC4	P43686	13	8	198	917	341	468	133	83	109	198	241	213	166	139	200
PSMC5	P62195-2	16	7	550	603	153	249	96	99	81	396	497	178	259	283	177
PSMC6	P62333	17	3	388	152	22	42	23	21	24	48	54	56	59	41	59
PSMD1	Q99460	21	12	506	218	212	434	148	84	308	358	384	470	361	373	459
PSMD11	O00231	16	7	511	201	101	173	68	61	92	157	136	168	166	133	194
PSMD12	O00232	12	8	284	334	120	169	75	40	84	115	136	150	117	98	145
PSMD13	Q9UNM6	15	9	439	1863	347	397	273	99	119	374	384	375	387	342	399
PSMD14	O00487	10	6	123	167	253	392	87	45	126	164	268	222	234	156	178
PSMD2	Q13200	30	16	845	2518	3486	2703	1338	1646	1563	1300	1311	1547	1529	1339	1576
PSMD3	O43242	18	11	623	192	121	502	159	88	162	388	362	468	400	370	467
PSMD4	Q5VWC4	6	3	200	112	154	134	62	51	73	76	68	61	79	98	54
PSMD5	Q16401	12	7	339	108	132	183	107	115	143	155	136	160	177	122	148
PSMD6	Q15008	17	8	432	171	87	153	53	57	75	170	203	208	160	176	160
PSMD7	P51665	6	2	242	37	68	79	27	21	48	61	114	156	69	61	80
PSMD8	K7EJR3	8	3	149	104	105	145	29	41	73	67	65	97	99	78	92
PSMD9	J3KN29	4	2	51	5	2	11	18	20	30	35	35	30	42	36	46
PSME1	A6NJG9	9	2	160	294	208	312	196	94	59	84	51	59	68	60	49
PSME2	Q9UL46	5	2	115	74	136	187	13	13	15	17	19	22	18	11	19
PSME3	P61289	10	5	412	91	61	42	45	43	76	184	292	155	197	203	203
PSPC1	Q8WXF1	22	12	523	745	312	321	151	199	196	909	1049	704	767	615	953
PTBP1	K7EK45	13	3	414	22	34	4	28	25	53	75	180	87	132	95	184
PTBP1	P26599	24	11	1027	3222	4265	7908	708	676	1628	3301	7184	2708	1712	2214	2061
PTBP3	O95758	17	12	442	226	642	852	270	175	308	960	1365	932	936	712	1087
PTK2	J3QT16	18	3	289	64	18	45	155	80	37	116	154	130	94	137	106
PTP4A2	Q12974	4	3	45	159	191	388	355	379	326	366	323	393	534	465	416
PTPLAD1	H3BPZ1	3	2	81	7	34	53	8	6	13	27	30	33	14	15	25
PTPN1	B4DSN5	8	5	128	312	417	485	2251	2063	3118	1649	1769	1975	1877	1639	2320
PTPN12	Q05209	5	2	79	23	7	5	36	40	40	52	38	57	40	32	46
PTPN13	Q12923	19	4	139	363	532	598	111	106	126	242	177	252	264	285	311
PTPN14	Q15678	20	9	211	174	116	135	178	146	114	322	383	356	322	278	306
PTPN22	E9PMT0	6	2	48	560	334	252	341	286	328	227	199	236	251	225	265
PTPN23	Q9H3S7	20	9	168	308	91	49	112	77	128	120	129	155	120	108	109
PTPRK	F5GXI4	9	4	84	16	21	18	17	15	11	26	24	14	11	20	16
PTRF	Q6NZI2	18	13	956	869	2561	2753	1059	1441	1502	4982	4660	5366	5992	5072	6239
PUF60	Q9UHX1-2	22	5	752	768	267	372	2846	3825	3159	2443	1631	1441	2470	2570	1921
PUM1	H0YEH2	11	5	130	89	26	25	56	66	33	82	156	155	138	108	180
PURA	Q00577	8	2	208	108	60	44	80	74	54	148	211	241	238	218	160
PURB	Q96QR8	9	4	150	19	70	31	90	127	120	130	244	265	226	223	186
PWP1	Q13610	5	3	94	20	28	11	2	1	4	6	12	7	5	3	6
PWP2	Q15269	13	9	326	1389	108	125	146	106	98	152	182	135	145	127	151

PXDN	Q92626	22	17	513	777	1506	1095	1259	1610	1102	1010	725	897	988	1206	976
PXN	F5GZ78	8	3	183	64	47	126	444	309	297	297	283	275	339	318	296
QARS	P47897	31	14	898	834	382	405	617	496	738	1111	1192	1399	1106	1131	1320
QRICH1	Q2TAL8	4	2	110	52	25	15	9	4	3	18	12	20	25	7	8
RAB11FIP1	J3KNP0	4	2	24	20	43	87	576	576	526	280	178	262	316	276	329
RAB13	P51153	10	3	226	75	91	91	368	399	184	379	309	415	404	434	351
RAB14	P61106	6	3	82	65	27	63	22	14	18	55	41	38	44	37	36
RAB5C	P51148-2	4	2	91	261	140	323	87	87	104	159	134	168	164	144	140
RAB6B	B7Z337	4	2	54	3019	150	147	594	130	181	264	165	180	218	191	184
RAC1	P63000	9	4	246	448	598	607	657	870	1124	640	615	747	1012	1020	635
RACGAP1	Q9H0H5	6	2	107	123	0	11	23	44	9	16	11	15	7	18	44
RAD21	O60216	14	9	401	120	246	200	39	24	36	226	303	192	150	106	183
RAD50	Q92878	54	19	928	345	286	337	181	118	166	434	439	343	340	281	367
RAE1	P78406	22	2	604	3	16	12	11	3	5	18	34	17	22	17	23
RAI14	Q9P0K7-4	55	43	1828	546	929	1242	4221	4405	4404	3719	3469	3560	3636	3662	6692
RALB	B4E040	5	2	121	125	0	6	20	26	28	37	25	51	46	46	35
RAN	J3KQE5	11	9	403	1824	2890	3599	1037	1033	1503	1934	1963	1812	2474	1924	2264
RANBP2	P49792	109	58	3138	2891	1667	2247	889	697	1096	1736	2045	1439	1414	1195	1657
RANBP3	B7Z7F3	5	2	150	455	15	26	48	4	2	20	15	10	7	3	15
RANBP9	Q96S59	6	3	78	18	16	24	9	11	9	21	20	27	17	22	30
RANGAP1	P46060	30	13	1032	498	436	1402	232	156	338	630	735	669	480	483	671
RAP1A	P62834	7	2	241	234	16	14	140	126	137	63	49	54	74	82	58
RAP1GDS<br>1	E9PH06	4	3	48	51	70	64	964	844	1265	493	494	702	543	652	812
RAPH1	C9K0J5	11	3	114	188	55	28	26	22	19	37	50	32	48	26	23
RARS	P54136	35	20	1225	1250	405	773	676	517	523	1054	1126	1273	1116	1026	1269
RASA1	B4DTL2	8	3	75	16	11	13	28	23	22	33	35	44	38	30	30
RASSF8	Q8NHQ8	8	3	60	5	2	1	21	27	22	55	60	69	59	63	69
RAVER1	E9PAU2	19	15	315	142	226	202	251	239	287	753	802	650	547	558	765
RBBP5	Q15291	11	2	158	18	7	13	3	0	4	7	10	3	1	2	1
RBBP6	Q7Z6E9	17	2	86	2	4	5	15	58	16	14	21	5	15	21	9
RBBP7	E9PC52	14	6	397	84	119	256	23	11	74	107	218	145	70	71	104
RBM10	P98175	8	5	185	185	28	58	64	52	34	90	81	72	56	48	80
RBM12	Q9NTZ6	12	4	104	26	38	71	26	17	19	63	50	40	45	38	67
RBM12B	Q8IXT5	12	4	185	87	45	48	27	6	10	56	69	43	26	20	35
RBM14	Q96PK6	21	14	596	1142	1418	1304	1216	1000	1447	1916	1716	1852	2226	1828	2122
RBM15	Q96T37-3	12	5	167	29	22	8	55	59	81	68	70	62	39	49	73
RBM17	Q96I25	11	5	201	23	38	55	6	4	15	34	46	23	17	13	30
RBM19	Q9Y4C8	10	9	336	82	35	29	133	122	74	217	212	161	186	165	185
RBM22	Q9NW64	9	5	235	410	55	47	60	33	93	129	160	100	179	92	138
RBM25	P49756	20	11	335	309	244	231	107	199	133	453	472	396	497	398	469
RBM26	Q5T8P6	16	8	313	55	90	70	68	111	160	76	136	99	128	153	128
RBM27	Q9P2N5	11	3	314	100	49	40	49	16	30	41	48	67	36	32	35
RBM28	Q9NW13	16	7	401	134	12	18	14	7	12	26	53	29	25	14	29
RBM3	P98179	5	4	119	186	127	124	609	581	512	637	862	899	1105	828	1010
RBM34	P42696	13	2	277	52	106	18	3	20	6	20	32	20	15	16	21
RBM39	Q14498	16	10	589	775	339	658	288	273	375	708	659	485	462	387	577

RBM42	K7EP90	5	5	214	68	40	53	44	23	20	103	156	71	73	74	79
RBM45	Q8IUH3	3	2	42	2	229	206	88	170	194	31	71	138	124	119	99
RBM47	A0AV96	4	3	52	5	14	5	5	1	10	7	32	23	8	10	7
RBM6	E9PGM9	10	4	72	421	33	55	60	28	51	78	90	96	95	73	114
RBM8A	Q9Y5S9	7	5	184	40	50	101	69	61	58	260	350	219	303	243	252
RBMS1	B4DN88	4	2	104	117	165	210	131	109	191	204	234	246	200	170	241
RBMS2	Q15434	4	3	140	52	30	31	82	36	50	108	77	75	101	57	81
RBMX2	Q9Y388	2	2	57	659	4	18	366	339	361	179	175	189	270	242	301
RBMXL1	Q96E39	30	3	821	0	0	0	1	1	1	5	26	10	7	6	7
RBMXL2	O75526	11	2	247	41	26	21	23	22	19	15	23	45	23	23	54
RBMXL3	Q8N7X1	7	2	116	47	144	92	349	298	247	177	186	229	227	216	303
RBPMS	B4E3T4	6	4	159	31	38	66	28	24	35	91	128	109	110	83	146
RCC1	P18754	12	2	435	13	22	29	25	16	19	168	245	114	123	116	119
RCC2	Q9P258	14	12	439	1437	437	882	308	210	370	542	626	420	436	426	458
RCL1	Q9Y2P8	10	6	250	1583	61	145	151	15	20	76	99	57	47	68	71
RCOR1	J3KN32	8	2	126	0	2	0	0	0	0	3	10	8	3	0	1
RDX	P35241	43	14	1487	344	623	610	672	573	836	968	1233	1089	982	1001	1281
RECQL	P46063	30	21	1069	360	172	318	46	44	105	403	607	383	291	221	390
RER1	Q5T091	4	2	90	51	3	15	22	19	28	20	14	7	21	16	11
REXO4	Q9GZR2	7	3	50	12	2	6	6	7	1	7	9	7	6	5	7
RFC1	P35251	25	9	430	130	253	244	222	250	292	370	294	386	343	335	448
RFC2	P35250	8	3	226	41	26	36	7	2	9	25	35	27	14	16	29
RFC3	P40938	6	4	147	3	36	46	11	12	23	29	83	51	33	53	66
RFC4	P35249	13	5	463	1324	123	131	241	73	137	115	124	91	91	96	80
RFC5	P40937	11	2	239	3	27	14	1	5	10	11	26	26	16	7	23
RHOG	P84095	6	2	128	151	20	31	88	68	49	74	63	93	87	103	60
RIC8A	Q9NPQ8	7	3	125	36	45	82	201	248	278	326	290	340	392	341	390
RIC8B	B7WPL0	3	2	75	81	191	218	287	287	188	420	274	367	518	367	547
RIF1	Q5UIP0	40	10	673	30	45	53	29	39	45	47	66	36	29	17	26
RMND5A	Q9H871	4	2	36	52	136	112	57	66	88	213	225	205	231	205	325
RNF20	Q5VTR2	18	5	212	159	321	212	131	98	138	199	143	191	203	205	225
RNF213	Q63HN8	111	72	2655	2185	999	1423	691	535	701	1713	2118	2074	1738	1451	1776
RNF40	O75150	15	5	125	5	19	22	36	77	135	36	42	29	31	35	46
RNGTT	O60942	7	2	70	500	547	463	3231	3290	4562	1341	1424	1450	1809	1672	1883
RNH1	P13489	18	13	526	951	533	838	1089	879	892	1300	1306	1109	1592	1298	1334
RNMT	O43148-2	7	4	105	70	22	27	214	138	228	128	141	262	595	228	150
RNPC3	A8K1C9	5	2	48	8	0	4	5	7	6	11	15	14	9	8	11
RNPS1	H3BMM9	11	7	258	716	187	165	183	150	104	247	293	191	170	167	177
ROCK1	Q13464	26	2	186	8	3	14	5	8	4	7	9	1	4	7	7
ROCK2	O75116	28	13	228	557	561	981	1247	837	500	1082	991	1011	1076	986	1065
RPA1	P27694	22	11	529	249	913	1127	82	108	132	296	459	335	247	255	325
RPA2	P15927-3	6	4	145	334	111	201	85	62	118	556	125	141	148	134	171
RPA3	P35244	4	2	135	42	65	78	65	49	46	100	116	144	172	118	170
RPF2	Q9H7B2	17	13	445	327	172	366	99	63	268	156	291	191	148	112	239
RPL10A	P62906	13	8	401	432	545	682	725	676	799	1061	1443	1424	1612	1351	1326
RPL11	P62913	6	2	189	3201	2839	4548	847	686	1121	779	863	1071	1135	907	885
RPL12	P30050	10	5	386	3238	6884	6869	2063	2139	2267	2021	2436	2846	3226	2613	2711

RPL13	P26373	11	5	329	172	383	408	227	176	282	1081	1378	1399	1661	1236	1437
RPL13A	P40429	12	3	158	2	47	47	36	50	42	133	196	186	218	167	195
RPL14	P50914	8	5	355	104	62	134	59	77	95	301	486	480	673	379	526
RPL15	P61313	9	4	266	106	36	44	42	39	32	101	198	132	125	104	121
RPL17	J3KRX5	12	3	355	215	609	741	95	116	207	419	816	593	577	494	395
RPL18	J3QQ67	19	7	411	102	395	356	65	98	121	655	830	1056	1017	649	802
RPL18A	M0R117	11	6	272	1320	581	576	323	280	319	875	1300	1109	1470	989	973
RPL19	P84098	9	3	208	74	16	70	71	32	17	165	265	203	278	119	162
RPL21	P46778	11	5	306	189	89	122	81	110	55	437	707	701	712	562	593
RPL22	P35268	9	5	465	3790	1109	1189	1420	827	1287	1286	1463	1188	1732	1247	1141
RPL23	P62829	11	10	381	702	1707	1897	1320	1328	1401	1993	2524	2918	2731	2186	2334
RPL23A	K7EJV9	12	3	247	57	102	168	18	23	22	85	98	126	118	93	105
RPL24	P83731	9	4	228	399	326	279	102	94	129	364	448	538	590	430	475
RPL26	J3QRC4	10	4	207	45	76	75	25	53	55	197	281	352	376	297	289
RPL27	P61353	12	7	180	143	281	284	273	297	257	829	1077	1119	1265	981	1003
RPL27A	P46776	6	5	151	285	656	539	168	191	231	573	791	761	923	591	700
RPL29	P47914	5	2	77	56	110	93	46	22	35	123	162	98	141	88	79
RPL3	P39023	21	4	529	216	361	590	314	286	403	534	990	693	486	503	543
RPL30	E5RI99	10	6	359	493	820	802	1122	1143	1103	1311	1595	1592	1669	1375	1481
RPL32	D3YTB1	6	4	141	192	43	42	23	37	24	114	213	166	191	108	100
RPL35	P42766	4	3	202	62	228	175	84	109	130	337	558	505	602	351	374
RPL35A	P18077	9	4	198	415	428	507	183	175	209	440	553	558	592	530	580
RPL36	Q9Y3U8	4	2	149	68	97	81	91	83	95	189	302	248	329	211	179
RPL36A	H0Y5B4	4	2	44	11	14	32	15	18	21	91	120	122	160	77	99
RPL37A	P61513	4	3	57	27	185	372	29	44	50	252	382	333	339	294	253
RPL38	P63173	4	3	191	274	882	790	476	372	522	509	487	553	568	636	436
RPL4	P36578	27	9	1039	281	519	730	124	173	246	1121	1627	1592	1769	1133	1728
RPL5	P46777	20	13	515	809	688	987	398	434	476	1189	1999	1982	1563	1360	1305
RPL6	Q02878	16	8	550	466	581	899	201	197	177	990	1787	817	1246	751	792
RPL7	P18124	23	9	607	254	242	301	374	436	288	798	1307	920	1245	920	1223
RPL7A	P62424	17	5	550	799	360	408	223	226	172	864	1190	1228	1172	979	1072
RPL7L1	Q6DKI1	8	4	209	28	41	21	4	5	10	27	51	24	23	16	19
RPL8	P62917	10	5	343	1798	235	548	295	190	190	1002	1455	1161	1386	1067	1165
RPLP1	P05386	2	2	32	148	116	339	689	529	158	1045	1649	1490	1075	1021	944
RPLP2	P05387	4	2	237	167	48	199	501	262	268	341	386	611	548	362	393
RPLP2	H0YDD8	7	2	178	7	188	107	5	5	131	2	4	73	5	3	35
RPN1	P04843	19	16	429	3961	822	1104	761	216	293	688	621	647	680	507	459
RPN2	P04844	12	7	379	187	167	346	84	35	63	118	183	151	76	96	87
RPP30	Q5VU11	8	6	152	199	70	272	96	86	98	125	147	152	148	145	223
RPP38	P78345	8	5	65	28	47	51	2609	2503	2616	747	763	731	1132	959	970
RPP40	O75818	4	3	69	10	85	83	20	5	45	23	48	26	14	18	16
RPRD1A	Q96P16	9	2	99	11	5	21	7	2	6	26	22	19	30	29	31
RPRD1B	Q9NQG5	12	2	322	131	221	158	33	38	100	96	126	96	73	68	118
RPRD2	Q5VT52	21	11	325	182	103	103	58	98	68	88	75	68	75	63	76
RPS10	P46783	9	5	228	306	357	473	819	679	684	1425	1977	2014	1892	1683	1721
RPS11	P62280	15	7	440	673	778	736	838	864	1182	1457	2030	1922	2216	1835	1746
RPS12	P25398	9	8	306	416	595	650	1136	992	1167	1235	1674	1511	1888	1396	1385

RPS14	P62263	10	10	399	2803	4782	4998	1538	1209	1509	2171	2521	3016	3791	2694	3048
RPS15	P62841	5	2	61	200	44	87	83	23	25	71	174	23	33	31	10
RPS15A	P62244	16	3	535	535	1066	934	873	593	811	940	1058	1058	1220	1010	838
RPS16	P62249	10	6	457	1479	2817	2790	1059	1179	1786	2073	2368	2839	3154	2458	2884
RPS17L	P0CW22	8	8	352	137	522	297	268	199	279	507	963	652	653	512	373
RPS18	P62269	16	5	554	773	2580	2368	957	1009	1508	1809	2209	2430	3608	2573	2527
RPS19	P39019	12	4	354	236	538	437	542	377	438	644	768	791	815	694	624
RPS19BP1	Q86WX3	4	2	78	5	6	0	0	0	0	10	32	20	15	9	16
RPS2	P15880	21	7	766	524	501	796	1033	983	1174	1803	2023	2396	2412	1933	2237
RPS20	P60866	8	4	189	1044	2582	1972	430	342	416	493	616	669	797	571	599
RPS21	Q8WVC2	5	3	187	32	132	183	23	14	29	46	68	81	91	59	53
RPS23	P62266	3	2	127	101	121	209	153	161	173	514	696	678	753	560	582
RPS24	E7ETK0	8	6	185	890	1784	1239	715	1099	729	991	848	947	1050	744	813
RPS25	P62851	6	3	198	160	561	477	157	148	175	561	667	716	875	662	681
RPS27A	P62979	14	4	527	4256	6776	7149	3483	3245	2704	8449	10445	7745	8303	7570	7629
RPS28	P62857	5	3	226	405	699	907	932	975	1270	867	709	901	1160	1229	1310
RPS29	P62273	3	2	48	137	445	373	73	54	96	99	125	115	143	143	100
RPS3	P23396	25	4	774	1483	1944	3005	658	566	843	801	797	978	1179	1008	1045
RPS3A	P61247	26	6	858	929	1297	1286	362	286	526	844	1547	1049	946	1005	792
RPS4X	P62701	24	10	837	2687	1083	1656	841	608	976	1827	3008	2299	2047	1691	1584
RPS4Y1	C9JEH7	10	2	366	194	37	18	16	6	23	75	95	57	30	51	26
RPS5	P46782	18	9	627	1693	2641	3164	1177	1053	1282	1478	1873	1694	2314	1700	1749
RPS6	P62753	7	2	331	201	307	234	103	143	115	528	729	611	739	481	546
RPS7	P62081	11	7	330	1817	2213	2420	623	532	991	912	1846	1377	1196	1012	1527
RPS8	P62241	12	8	473	481	692	1193	442	425	443	1700	2588	2134	2407	1718	1665
RPS9	P46781	16	8	499	446	502	442	337	390	443	950	1198	1253	1482	1146	1224
RPSA	C9J9K3	13	12	443	1946	1870	2273	864	729	615	1440	2107	1971	1857	1508	1281
RQCD1	Q92600	9	5	174	160	370	226	286	273	367	249	305	289	373	317	388
RRAD	P55042	6	2	98	79	113	222	113	113	144	110	92	129	141	144	118
RRAGA	Q7L523	5	2	22	5	3	2	17	22	26	39	49	40	46	50	42
RRAS2	B7Z5Z2	7	3	153	24	38	13	24	16	29	26	26	24	26	26	15
RRBP1	Q9P2E9	55	34	1505	864	315	506	349	314	341	2198	2419	2424	2308	1633	2243
RRM1	E9PD78	10	5	102	85	212	38	125	105	332	28	23	43	63	50	87
RRP1	P56182	20	11	367	94	262	216	108	111	97	354	494	294	332	253	305
RRP12	Q5JTH9	28	4	637	86	12	33	12	4	9	45	53	33	34	22	56
RRP1B	Q14684	18	10	397	76	34	40	35	42	18	104	130	103	69	65	99
RRP7A	Q9Y3A4	5	3	139	13	59	7	6	4	3	29	72	18	14	13	15
RRP8	O43159	11	5	196	54	48	29	18	20	24	71	93	67	82	41	66
RRP9	O43818	10	4	286	6	8	8	3	10	8	63	79	44	43	31	61
RRS1	Q15050	13	5	158	47	33	10	19	12	11	20	48	18	20	12	20
RSBN1	Q5VWQ0	6	4	52	195	171	190	346	341	410	355	281	294	389	307	441
RSBN1L	Q6PCB5	6	2	100	2	2	3	69	72	82	60	46	41	55	55	56
RSF1	Q96T23	19	11	493	117	66	31	60	22	70	38	61	14	23	7	15
RSL1D1	O76021	17	11	536	1054	1228	1448	217	226	345	703	800	559	598	418	662
RSL24D1	Q9UHA3	8	3	49	592	17	25	92	3	10	17	29	7	7	6	2
RSU1	Q15404	11	4	208	91	115	151	707	565	465	643	755	842	744	1009	819
RTF1	Q92541	5	2	79	2	0	6	2	4	9	4	9	5	3	4	9

RUFY1	Q96T51	16	2	75	13	33	37	190	157	171	175	155	173	177	183	211
RUFY2	H0YD93	13	4	80	11	11	20	132	171	212	102	179	943	178	131	235
RUVBL1	Q9Y265	21	17	807	642	958	1469	1154	1023	1035	2321	2558	2422	2541	2005	3070
RUVBL2	B3KQ59	21	15	838	648	758	1297	756	653	758	1418	1470	1342	1232	1082	1602
S100A10	P60903	5	3	146	28	60	107	644	374	411	461	648	539	518	421	456
S100A11	P31949	6	6	266	1168	818	1106	1692	1388	1185	1246	2286	957	1440	1608	959
S100A13	Q99584	6	3	132	29	78	143	270	241	365	281	328	363	352	393	253
S100A16	Q96FQ6	6	5	130	98	129	234	500	469	489	602	587	589	697	720	574
S100A7	P31151	3	3	37	17	19	18	55	47	59	23	22	26	28	21	22
SAA1	P0DJI8	2	2	48	1	4	6	156	110	72	27	27	38	44	51	30
SACS	Q9NZJ4	64	33	1467	2055	234	282	567	377	416	904	940	1076	946	823	922
SAFB2	Q14151	23	6	355	53	19	51	26	25	67	113	131	87	83	54	98
SAMHD1	Q9Y3Z3	26	16	862	607	325	486	267	185	295	386	425	468	386	395	532
SAMM50	Q9Y512	8	4	211	69	31	33	19	41	25	20	34	35	48	45	68
SAP18	O00422	15	8	406	83	66	113	102	106	192	152	215	207	221	203	192
SAP30BP	J3QQJ0	7	4	127	65	98	131	235	428	419	157	93	143	158	212	172
SARS	Q5T5C7	13	8	209	128	221	279	168	168	193	190	168	214	190	166	217
SART1	O43290	28	17	683	1246	181	167	569	415	508	389	419	338	318	306	342
SART3	Q15020	20	13	435	235	266	179	108	97	115	234	284	216	228	182	258
SBDS	Q9Y3A5	7	3	116	11	3	9	139	176	161	119	119	110	153	122	107
SCAF1	Q9H7N4	11	4	91	460	1082	41	32	35	130	74	151	203	173	166	136
SCAF4	C9JLZ0	10	2	102	14	6	3	7	1	2	41	48	28	18	17	30
SCAF8	B7Z888	9	2	131	5	4	6	4	1	14	5	12	5	1	0	14
SCFD1	Q8WVM8	10	6	222	57	28	59	10	11	20	39	46	58	35	23	44
SCYL1	Q96KG9	7	4	65	7	1	433	11	13	12	34	28	30	20	23	17
SDAD1	E7EW05	16	3	158	36	102	107	197	238	328	210	181	225	262	217	211
SDC4	P31431	4	3	72	10	7	30	156	132	117	145	114	148	126	155	185
SEC11A	H0YK72	3	2	91	36	32	51	12	16	20	21	26	32	36	22	25
SEC13	B4DXJ1	9	7	300	615	194	245	369	275	81	705	837	790	653	648	560
SEC16A	H0Y5S1	12	2	93	908	11	19	30	19	14	80	75	76	104	79	105
SEC16A	J3KNL6	27	14	411	260	264	291	172	230	193	253	324	284	258	213	318
SEC22B	O75396	6	4	176	316	72	162	53	19	28	98	103	120	117	79	104
SEC23A	F5H365	17	13	494	123	131	200	306	259	414	606	529	685	529	691	558
SEC23B	Q15437	5	2	57	27	3	6	0	0	1	7	16	5	7	8	1
SEC24A	O95486	6	4	117	72	75	156	25	17	17	82	71	110	92	94	107
SEC24B	B7ZKM8	6	4	90	57	299	74	23	33	29	57	49	59	47	35	47
SEC24C	E7EP00	10	6	216	109	66	117	40	42	34	224	208	211	168	125	198
SEC24D	O94855	7	4	84	18	42	22	26	14	31	65	78	94	80	59	89
SEC31A	D6REX3	18	10	321	170	204	324	120	143	146	307	262	336	325	278	329
SEC61A1	B4DR61	8	4	128	55	56	79	69	90	54	174	235	253	190	182	173
SEC61B	P60468	3	3	82	334	556	467	341	253	466	383	597	670	586	687	501
SEH1L	Q96EE3	8	5	186	225	210	63	94	27	48	121	148	109	91	71	64
SELH	Q8IZQ5	2	2	52	52	145	170	46	54	50	77	86	63	106	93	74
SEMA7A	O75326	11	6	190	38	117	144	1104	884	1066	641	602	671	729	601	735
SENP3	J3KNH7	11	9	158	1380	410	219	151	65	92	116	122	99	102	84	103
SEPHS1	B4DWK0	4	3	88	0	1	4	1	1	6	8	19	12	7	3	15
SEPT10	B5ME97	12	7	293	742	1066	1145	692	738	493	510	547	544	477	528	497

SEPT11	D6RER5	28	7	878	4017	5460	4678	1415	1301	1486	2168	2421	2340	2063	2232	2284
SEPT7	Q16181	31	24	1025	6410	9816	9723	5989	5697	7555	7842	8145	6906	7447	7565	7629
SEPT8	A6NFQ9	15	7	524	1539	1643	2255	858	999	1090	580	619	699	607	628	749
SEPT9	Q9UHD8	42	15	1194	2330	5455	4026	4340	3912	3447	4904	4258	5331	4835	5106	5892
SERBP1	Q8NC51	8	5	252	76	127	132	100	85	92	332	339	219	169	185	205
SERPINB12	Q3SYB4	6	3	84	167	151	134	155	231	144	136	44	82	38	68	53
SERPINB6	P35237	8	6	176	60	51	129	97	84	113	170	195	186	172	164	182
SERPINE1	P05121	8	5	129	102	25	283	588	574	441	165	108	89	111	114	110
SERPINH1	P50454	31	26	1188	1146	1288	1219	43855	38810	36515	7897	6014	7877	6608	6663	6509
SET	Q5VXV2	6	3	183	440	504	595	154	135	191	515	637	368	409	328	390
SETMAR	Q53H47	6	3	43	327	664	680	233	254	266	290	288	320	357	358	303
SETX	Q7Z333-3	19	4	94	462	277	27	41	41	10	185	722	23	81	26	40
SF1	Q15637-6	8	5	132	57	41	40	75	110	104	309	378	277	351	267	388
SF3A1	Q15459	34	22	743	578	1082	1224	213	227	456	1049	1151	871	896	799	1189
SF3A2	Q15428	8	8	180	136	115	143	57	44	81	228	277	184	200	164	319
SF3A3	Q12874	20	15	647	445	429	527	329	430	483	917	982	884	814	605	1001
SF3B1	O75533	60	39	1794	1845	2135	2496	1351	551	947	2630	2887	1968	2047	1571	2269
SF3B14	Q9Y3B4	6	3	234	162	327	319	100	101	102	296	389	247	265	271	221
SF3B3	Q15393	47	33	1710	2654	1717	3250	857	843	1098	3442	3687	2900	2879	2522	3341
SF3B5	Q9BWJ5	3	3	72	9	2	6	2	2	1	34	61	48	33	19	20
SFN	P31947	8	3	236	2	21	14	8	9	8	5	7	5	6	5	43
SFPQ	P23246	35	24	1234	1941	2133	2053	1157	1151	1133	5683	5913	3816	5031	4180	5892
SFRS3	B4E241	9	4	186	2169	2459	2056	795	626	635	2997	3242	2783	3187	2885	3023
SFXN3	Q9BWM7	4	2	120	68	4	32	67	59	65	62	55	86	98	78	102
SGTA	K7EMD6	4	2	27	15	8	11	3	1	0	9	4	2	1	0	1
SH3BGRL2	Q9UJC5	3	2	65	2	3	4	138	157	231	62	69	83	84	67	94
SH3BP4	Q9P0V3	6	3	71	826	76	76	276	136	30	440	431	375	402	368	355
SH3GL1	Q99961	10	3	248	52	29	34	100	51	85	168	154	145	156	123	129
SH3KBP1	Q96B97	6	4	86	81	39	24	54	29	62	147	191	157	122	140	83
SIN3A	Q96ST3	24	16	366	503	400	353	348	684	1201	723	761	613	682	594	902
SIN3B	O75182	9	2	90	85	3	0	8	1	1	10	16	7	18	10	11
SIPA1	F6RY50	11	2	115	1	1	3	8	12	0	19	38	20	13	19	4
SIPA1L1	J3KP19	14	4	236	112	11	12	124	94	73	107	102	119	92	85	94
SIPA1L2	Q9P2F8	16	6	205	12	9	13	112	133	159	196	171	202	184	165	206
SIPA1L3	O60292	26	16	503	276	80	57	843	858	884	775	810	864	785	762	866
SKIV2L	Q15477	15	4	120	32	61	81	336	361	482	263	221	274	274	229	313
SKIV2L2	P42285	27	18	668	1566	693	781	3619	257	325	817	1040	585	690	886	726
SLC16A3	O15427	2	2	23	12	28	19	17	22	5	31	20	15	12	14	12
SLC25A1	P53007	6	3	175	5	29	39	140	238	335	137	122	192	209	221	213
SLC25A11	I3L1P8	7	5	149	510	10	29	84	65	85	85	94	93	107	115	86
SLC25A12	B3KR64	6	2	72	0	111	84	75	113	74	38	12	20	6	14	17
SLC25A13	Q9UJS0	12	5	173	133	48	59	75	111	131	94	77	118	87	76	84
SLC25A3	Q00325	15	10	353	351	374	464	721	591	475	804	682	822	763	779	628
SLC25A5	P05141	23	5	725	1138	909	843	1702	1911	2303	1462	1279	1706	1970	2091	1699
SLC2A1	C9JIM8	7	5	203	39	71	92	786	994	982	288	233	369	353	361	430
SLC9A3R1	O14745	5	3	69	732	56	29	228	50	51	120	151	197	220	149	195

SLFN5	Q08AF3	25	9	417	680	152	124	72	84	73	213	321	194	191	143	198
SLK	Q9H2G2	12	4	81	254	595	245	340	164	518	146	81	129	131	102	129
SLTM	Q9NWH9	19	7	214	351	244	78	133	89	90	192	201	118	153	126	169
SMAD3	P84022	8	3	90	32	7	21	44	55	86	98	103	100	112	90	115
SMARCA1	F6TQG2	38	7	999	76	84	62	92	91	96	114	151	124	107	89	114
SMARCA2	P51531	22	2	358	14	4	8	141	155	186	89	87	107	100	96	122
SMARCA4	Q9HBD4	24	4	517	97	60	83	133	147	156	181	215	162	175	146	215
SMARCA5	O60264	56	22	1710	325	299	371	152	119	181	728	861	514	527	408	653
SMARCB1	G5E975	9	6	152	215	199	427	169	68	76	308	264	302	295	214	404
SMARCC1	Q92922	20	7	352	115	155	62	46	34	64	117	129	77	77	59	110
SMARCC2	Q8TAQ2	23	10	495	192	102	121	65	160	143	177	215	120	100	80	218
SMARCD1	Q96GM5	12	5	285	35	17	26	13	7	25	24	43	24	27	11	26
SMARCD2	J3KMX2	10	5	219	182	7	28	22	8	1042	79	81	59	50	35	92
SMARCE1	C0IMW4	11	2	167	29	8	6	22	23	26	31	49	42	39	30	51
SMC1A	Q14683	62	34	1703	706	840	835	409	404	533	723	1004	521	520	517	556
SMC2	O95347	34	11	607	168	42	65	134	139	144	120	106	161	150	137	162
SMC3	Q9UQE7	56	37	1530	2878	1054	1902	730	502	637	1662	1655	1312	1302	1144	1529
SMC4	E9PD53	31	12	406	233	102	112	318	261	175	245	294	237	349	295	188
SMC6	Q96SB8	18	5	206	118	21	68	7	4	11	24	29	22	13	12	17
SMCHD1	J3KRK8	5	2	32	31	68	70	10	12	13	184	213	183	206	144	237
SMCHD1	A6NHR9	58	31	997	849	268	272	337	96	153	480	625	427	351	262	526
SMEK1	Q6IN85	7	2	110	7	5	11	1	1	4	8	8	4	2	4	2
SMN1	E7EQZ4	3	3	70	80	51	7	34	25	27	37	50	58	25	20	19
SMPD4	Q9NXE4	15	3	333	53	9	30	16	8	5	34	49	34	25	44	27
SMTN	P53814	18	4	327	284	655	118	1650	1551	2639	1024	844	1131	1736	1623	1860
SMU1	Q2TAY7	14	10	479	491	161	311	66	32	59	159	225	120	129	85	125
SND1	Q7KZF4	36	22	1063	2212	1265	1288	1418	1186	1570	2531	2565	2844	2773	2414	3001
SNRNP200	O75643	76	55	2431	2216	1402	2471	446	521	728	2140	2892	1808	1619	1428	1735
SNRNP40	Q96DI7	15	13	476	424	808	1222	136	151	342	600	818	499	448	439	568
SNRNP70	P08621	10	2	219	9	6	23	15	12	11	23	36	23	18	24	20
SNRPA1	P09661	11	5	406	73	196	221	25	38	82	209	320	256	232	226	222
SNRPB2	P08579	9	5	246	302	104	222	23	24	62	49	78	30	28	48	50
SNRPD1	P62314	7	6	240	738	436	1087	1999	2478	2944	1500	1646	1599	1859	1667	1801
SNRPD2	P62316	9	6	325	1965	2913	2244	604	557	644	1072	1135	1112	1393	1274	1179
SNRPD3	B4DJP7	8	5	154	2334	3965	3833	1489	1726	2355	2419	2609	2881	3160	2844	3284
SNRPE	P62304	5	4	171	734	660	1211	363	432	1080	422	403	462	594	500	638
SNRPF	P62306	4	3	231	164	318	473	109	94	144	308	290	274	348	328	308
SNRPGP15	A8MWD9	5	2	176	154	408	458	103	110	151	260	363	273	269	250	208
SNRPN	B3KVR1	11	5	184	283	568	580	208	187	328	742	819	809	854	710	776
SNTB1	Q13884	11	4	115	105	176	130	92	102	159	173	478	521	254	286	326
SNTB2	Q13425	15	8	233	42	96	140	568	505	646	498	462	551	558	572	650
SNW1	G3V3A4	17	9	365	225	114	111	32	58	45	126	234	77	109	77	101
SNX1	A6NKH4	5	2	151	50	25	48	40	34	56	83	61	78	84	58	87
SNX2	B4DEK4	7	3	201	8	7	10	28	25	14	36	36	49	42	38	42
SNX9	B3KXH8	5	4	58	170	86	179	2681	2339	2799	975	1123	1048	1493	1122	1441
SOAT1	P35610	4	2	43	0	1	1	17	32	12	7	14	25	16	16	24

SOD2	B3KUK2	7	4	159	557	224	409	85	65	64	197	268	396	259	214	251
SOGA2	Q9Y4B5	23	3	123	63	66	3	9	7	0	85	265	9	22	7	6
SON	P18583-5	26	5	638	21	78	59	15	8	8	89	104	46	31	33	58
SORBS3	O60504	7	4	82	115	97	21	266	233	221	342	581	86	142	101	75
SP110	Q9HB58	14	5	192	24	20	17	14	16	18	28	37	34	17	15	23
SPAG11A	H0YEK6	7	2	55	14682	12638	22809	4477	3922	4296	10037	6733	9157	11748	10712	11854
SPARC	P09486	13	11	433	102272	7255	2167	22163	2053	1236	9223	1321	1420	8542	2034	1571
SPATA31C2	B4DYI2	5	3	31	684	158	65	81	40	43	63	73	64	53	47	83
SPATA5	Q8NB90	8	2	70	7	19	103	0	0	3	9	33	1	12	13	2
SPATS2L	Q9NUQ6-2	11	6	116	375	36	25	63	45	51	49	137	72	58	56	44
SPCS2	E9PL01	4	2	78	37	9	13	6	3	7	16	38	21	19	19	18
SPCS3	P61009	5	3	124	77	25	118	45	43	58	79	83	92	105	80	84
SPECC1	Q5M775	15	7	224	99	113	68	536	577	627	487	326	408	388	463	364
SPECC1L	F5H1H6	27	13	348	498	143	341	794	761	772	1039	948	867	985	883	1089
SPEN	Q96T58	24	3	140	32	32	61	17	8	14	57	80	39	60	41	46
SPG20	Q8N0X7	9	3	115	4	4	11	9	8	4	18	19	25	12	13	13
SPIB	M0R3H8	4	2	58	109	12	2	1	0	3	19	9	14	8	6	13
SPIN3	Q5JUX0	3	2	37	7	14	7	46	196	155	77	124	136	117	122	118
SPTAN1	Q13813-3	214	4	9809	531	1012	961	1504	1492	1810	2566	1913	2101	2364	2552	2229
SPTAN1	A6NG51	216	3	9858	116	150	158	187	167	77	238	285	257	189	214	186
SPTB	P11277	33	2	471	222	362	368	322	308	358	280	295	304	371	384	346
SPTBN1	Q01082	203	13	8845	476	1091	1045	4358	4354	5275	6542	6315	6707	7150	7365	6587
SPTBN2	O15020	59	22	1513	317	191	214	552	557	623	749	797	771	726	740	744
SPTBN5	Q9NRC6	43	5	252	432	740	446	313	512	342	240	114	196	173	152	185
SQRDL	H3BMS6	15	4	126	361	29	115	45	17	67	355	344	250	283	188	225
SQSTM1	E7EMC7	8	6	115	352	96	174	198	155	166	316	285	316	379	291	404
SRBD1	Q8N5C6	9	2	94	3	18	19	16	14	21	26	28	32	23	19	27
SRFBP1	Q8NEF9	5	3	48	14	29	23	103	37	157	215	168	138	32	22	45
SRGAP2	H0Y2P8	7	4	110	24	15	33	19	20	13	30	32	130	32	27	49
SRI	C9J0K6	4	2	38	26	43	56	5	5	10	8	9	11	13	10	11
SRP14	P37108	10	5	295	113	305	354	960	774	742	1654	1895	1742	1476	1888	1251
SRP68	F5H5Y3	12	2	219	1	9	19	30	27	35	34	24	35	30	52	45
SRP72	O76094	6	2	147	778	50	40	96	26	17	49	42	49	43	35	44
SRP9	P49458	5	3	149	215	215	297	362	336	566	251	268	281	342	339	266
SRPR	P08240	10	3	204	29	81	96	44	50	41	83	81	89	70	111	78
SRPRB	Q9Y5M8	8	4	255	107	34	101	112	103	117	125	146	159	184	147	151
SRRM1	Q8IYB3	14	6	166	172	105	45	211	260	318	216	192	261	219	199	227
SRRM2	Q9UQ35	36	19	630	412	475	392	485	655	544	803	843	885	613	689	734
SRRT	Q9BXP5	12	9	294	118	57	58	25	22	25	125	128	89	96	71	82
SRSF1	J3KTL2	13	8	440	380	402	532	262	230	328	778	782	648	681	639	677
SRSF10	O75494	7	2	160	88	1	13	4	0	3	7	21	5	2	4	3
SRSF12	Q8WXF0	6	2	148	11	13	4	7	4	7	5	6	6	5	5	11
SRSF4	Q08170	10	2	203	6	14	6	27	41	58	35	27	39	39	24	28
SRSF5	Q13243	7	3	197	43	108	122	25	27	30	145	172	99	110	109	142
SRSF6	Q13247	13	4	367	13	37	24	167	105	35	131	121	120	111	120	109
SRSF7	C9JAB2	9	6	249	799	748	1078	145	127	161	730	761	619	611	587	673

SSB	P05455	8	4	111	20	59	82	13	13	16	85	129	75	43	46	48
SSBP1	Q04837	9	6	416	475	502	769	433	310	342	506	737	523	623	514	533
SSFA2	E9PHV5	11	2	108	3	1	1	2	0	0	0	1	0	1	0	1
SSR4	P51571	4	2	125	33	52	35	10	11	11	49	105	54	17	33	17
SSRP1	Q08945	28	19	873	583	907	755	188	246	338	618	674	491	420	392	499
ST5	P78524	9	3	98	3	32	40	111	137	285	156	183	215	176	153	225
STAG2	F8WAK8	21	11	498	266	105	70	249	66	46	375	192	157	168	149	160
STAM	Q92783	4	2	63	15	30	26	119	142	171	221	218	183	261	242	228
STAT1	P42224	36	23	1300	989	1478	1882	1774	1521	1983	3079	3146	3269	3207	2769	3647
STAT2	G3V2M6	11	7	143	80	146	40	57	46	127	138	127	186	168	127	188
STAT3	K7ENL3	17	15	551	399	254	368	502	375	433	506	579	545	480	427	508
STAU1	O95793	11	5	126	64	49	32	95	38	73	204	157	109	132	115	110
STIP1	P31948	19	10	321	346	349	490	319	209	165	450	543	455	429	392	345
STOM	P27105	8	5	290	370	18	78	41	8	13	30	52	18	24	20	15
STOML2	B4E1K7	11	8	490	563	85	114	133	18	32	56	102	46	82	30	36
STRAP	B4DNJ6	10	6	279	473	68	122	376	248	161	353	362	426	369	337	381
STRBP	Q96SI9	16	6	368	93	149	264	319	326	400	324	302	328	400	366	428
STRIP1	Q5VSL9	13	5	187	273	313	223	227	239	174	222	156	186	140	137	138
STRN	O43815-2	7	5	135	164	63	72	135	112	111	105	127	111	120	133	124
STRN3	Q13033-2	13	4	341	62	23	41	480	423	465	381	407	439	473	403	512
STRN4	F5GYK2	6	3	82	2	2	5	141	133	148	137	129	139	124	122	148
STT3A	P46977	12	5	134	43	71	138	341	405	515	213	217	273	274	266	276
STT3B	Q8TCJ2	10	5	181	112	18	81	20	30	35	62	106	67	54	53	50
STX4	A8MXY0	8	4	30	166	118	206	972	989	1294	659	462	564	823	699	809
SUB1	P53999	7	6	154	113	820	606	240	250	360	451	410	400	418	461	453
SUGP2	M0R2Z9	17	4	288	11	8	6	8	3	4	13	25	8	13	6	16
SUGT1	F5H5A9	5	3	49	8	7	18	6	9	9	39	33	43	39	20	43
SUMO1	B8ZZN6	4	3	102	85	81	139	22	52	38	226	236	176	192	131	268
SUN2	Q9UH99	16	12	437	786	264	294	118	90	81	421	469	423	278	241	312
SUPT16H	Q9Y5B9	41	25	1040	1141	931	1508	445	513	862	1127	1103	1139	1056	854	1131
SUPT4H1	P63272	2	2	65	208	117	269	111	97	169	57	53	57	65	62	45
SUPT5H	O00267	18	13	450	272	66	93	40	26	52	115	145	99	88	78	128
SUPT6H	Q7KZ85	29	18	550	190	330	185	185	172	181	251	243	204	210	188	240
SURF4	Q5T8U5	3	2	73	11	4	4	6	5	3	11	9	15	3	7	7
SURF6	O75683	7	2	72	10	7	3	0	0	0	5	1	1	1	1	2
SUZ12	J3QQW9	6	5	97	10	12	8	3	3	4	8	24	10	6	6	8
SVIL	O95425	79	3	2166	12	6	3	91	104	133	157	140	161	159	113	176
SWAP70	Q9UH65	14	5	126	478	965	980	3783	4928	6283	2080	1780	2186	2370	2049	2413
SYCE1	Q8N0S2	8	2	55	3	3	0	6	10	4	15	14	18	13	10	7
SYCP1	Q15431	13	2	102	6	5	17	26	14	62	24	26	23	34	21	38
SYMPK	Q92797	29	12	677	145	81	78	71	66	106	121	148	83	74	51	97
SYNCRIP	O60506	35	2	1307	24	17	7	1	3	2	93	131	63	66	42	86
SYNE1	E7ENN3	76	12	472	1113	829	871	3668	3728	4450	2548	2186	2471	2874	2963	3259
SYNE2	G3V5X4	118	2	2168	23	41	5	42	19	8	32	61	27	26	25	16
SYNPO	Q8N3V7	5	2	40	26	4	5	26	39	43	19	41	54	57	40	76
TAB1	Q15750	6	4	147	204	503	470	114	94	145	688	1058	1408	1073	758	406
TAF1	P21675-4	13	4	62	945	1494	1339	647	853	777	624	401	572	629	576	635

TAF15	K7EPT6	7	2	121	32	18	16	48	37	49	129	99	129	103	80	145
TAF4	Q5TBP5	6	3	170	34	20	16	225	188	249	87	114	127	97	112	132
TAF5	Q15542	13	2	111	168	119	181	229	404	239	223	77	84	81	135	73
TAF6	P49848-3	8	2	135	9	21	2	0	1	0	2	7	4	4	1	3
TAF9	D6RIE8	7	2	53	27	13	16	8	17	17	15	25	23	24	12	27
TAGLN	Q01995	10	6	241	77	86	124	183	148	163	180	241	225	244	233	206
TAGLN2	P37802	14	6	646	638	906	1344	2142	1817	2093	1501	1512	1832	2244	1681	1986
TAGLN3	C9J5W6	6	2	191	275	18	15	52	37	44	98	116	108	95	62	117
TALDO1	P37837	13	6	368	456	466	751	57	96	130	124	177	154	127	65	181
TANC2	Q9HCD6	12	2	102	121	34	26	11	10	14	24	34	32	20	21	19
TARDBP	Q13148	10	3	387	20	47	117	14	19	41	193	243	144	192	131	198
TARS	P26639	16	5	305	42	14	49	86	82	59	163	183	188	119	108	123
TBC1D1	Q86TI0-2	13	2	135	831	37	33	123	7	10	12	12	6	5	3	8
TBC1D15	Q8TC07	6	2	69	0	0	3	13	19	22	9	16	21	18	17	19
TBCB	A8MVD5	6	2	145	36	12	13	14	11	13	12	17	19	24	17	13
TBK1	Q9UHD2	9	4	155	18	9	8	47	27	15	56	62	81	50	52	62
TBL1X	O60907	8	2	159	91	25	11	4	3	5	271	337	159	361	182	289
TBL1XR1	Q9BZK7	12	9	441	186	100	148	138	109	190	192	310	199	177	200	231
TBL2	E9PF19	9	4	202	19	5	7	13	30	23	82	90	94	76	65	73
TBL3	Q12788	25	4	671	26	44	70	9	4	18	37	52	31	23	21	32
TCEB1	E5RHG8	3	3	82	27	54	75	9	84	9	8	50	40	36	31	35
TCEB3	Q14241	12	4	111	92	2	8	21	8	15	60	49	35	51	43	61
TCERG1	O14776	18	5	333	46	82	75	56	55	61	205	201	124	128	111	163
TCIRG1	Q13488	15	10	335	83	37	28	23	30	42	76	119	90	83	65	87
TCOF1	E9PHK9	19	12	462	99	70	89	219	191	224	341	312	247	271	225	294
TCP1	P17987	29	12	1012	250	303	564	347	290	353	870	993	871	573	768	511
TCP11L1	E9PJ55	4	2	41	9	7	3	38	28	64	6	5	11	9	13	18
TECR	B3KSQ1	7	5	87	85	25	52	103	58	52	151	148	196	164	157	177
TERF1	P54274	3	2	33	0	2	8	3	7	4	9	9	11	9	5	12
TES	H7BYK1	8	3	128	8	24	10	28	220	144	26	98	100	112	108	80
TEX10	Q9NXF1-2	32	13	943	601	270	277	195	56	91	426	608	459	377	273	499
TF	P02787	46	30	2300	214310	266682	216682	521	542	1049	470	403	542	517	403	459
TFAM	Q00059	7	2	134	14	8	15	16	13	8	24	56	35	42	23	22
TFG	G5E9V1	6	3	68	25	34	76	57	21	70	43	38	39	38	32	43
TFIP11	Q9UBB9	6	5	87	470	252	266	50	41	39	147	175	137	154	111	150
TFPI2	P48307	12	10	435	234	361	449	849	1165	1087	2759	2936	3389	4791	5989	4365
TFRC	P02786	16	11	343	1631	3052	3042	39	37	38	38	21	25	35	38	35
TGFB1I1	O43294	5	5	125	13	8	5	303	229	290	123	109	113	139	129	99
TGFBI	G8JLA8	18	8	344	546	700	706	1739	2138	2136	1078	732	1081	1170	1479	1361
TGM2	B4DIT7	40	27	1260	3200	1908	2506	1361	999	1158	12481	11385	13789	13628	15175	13126
TGM4	P49221	5	2	60	28	12	21	46	47	19	83	89	100	110	81	105
THBS1	P07996	18	11	658	1168	516	623	1032	964	1576	1134	662	923	1376	1647	1155
THOC1	Q96FV9	18	8	461	457	107	60	150	106	134	184	215	174	156	130	165
THOC2	Q8NI27	22	8	459	139	96	88	76	117	177	175	172	142	157	138	181
THOC3	Q6NZ53	6	2	91	4	16	19	10	12	26	50	41	41	45	32	48
THOC5	Q13769	13	6	204	122	79	85	97	164	85	131	120	123	109	88	119
THOC6	Q86W42	9	6	188	247	159	153	344	460	334	501	450	448	491	479	711

THRAP3	Q9Y2W1	25	16	665	1797	232	258	237	211	300	719	706	485	508	468	678
TIAL1	Q01085-2	8	5	233	131	35	67	25	19	24	81	64	69	75	52	88
TINAGL1	Q9GZM7	7	5	171	74	47	18	655	587	702	347	317	393	554	516	363
TIRAP3	Q6JUT2	4	3	75	23	184	98	7	10	16	16	16	21	17	15	14
TJP1	G3V1L9	39	25	953	208	404	593	2226	2251	1989	2262	1786	2256	2315	2105	2602
TJP2	Q9UDY2	32	14	734	105	138	213	932	975	1056	622	639	676	620	568	748
TK1	K7ERV3	6	2	43	5	1	4	31	41	46	7	12	15	10	19	12
TKT	B4E022	22	15	532	1461	1927	2851	673	583	1124	1514	2205	1482	1368	1092	1734
TLK2	Q86UE8	12	3	153	43	13	6	2	9	4	5	9	8	6	2	6
TLN1	Q9Y490	170	119	7731	8913	11154	11430	90616	76348	71483	55978	49593	55287	58141	58931	54493
TLN2	Q9Y4G6	71	16	2194	82	67	40	1408	1308	1460	703	592	612	622	706	601
TMA16	H0Y9X1	4	2	64	63	9	18	16	14	5	41	56	39	49	45	49
TMED10	P49755	5	4	239	327	133	101	32	18	29	39	37	26	40	32	25
TMED2	F5GX39	3	3	32	124	45	134	59	11	33	35	153	33	38	15	16
TMEM165	Q9HC07	2	2	83	17	6	42	0	1	2	1	2	3	2	0	1
TMEM201	H0Y4R5	8	6	120	340	46	56	28	27	22	97	129	77	87	55	118
TMEM209	Q96SK2	11	10	193	135	115	39	92	89	166	50	74	32	31	146	30
TMEM214	Q6NUQ4	14	7	256	277	50	31	32	35	23	124	141	100	114	67	92
TMEM43	Q9BTV4	6	4	105	16	25	22	7	3	6	10	27	15	9	7	7
TMF1	P82094	14	6	95	691	857	651	292	238	335	369	414	418	400	397	424
TMOD3	Q9NYL9	10	2	397	17	23	17	228	133	64	187	221	232	232	187	216
TMPO	P42167	21	9	590	491	795	1116	313	286	714	892	1141	783	755	647	938
TMPO	P42166	25	13	778	199	370	408	44	44	194	138	155	117	107	89	136
TNC	J3QSU6	23	8	408	109	27	116	345	402	319	313	220	310	428	463	367
TNFAIP2	Q03169	13	5	306	163	61	118	131	87	82	141	113	109	104	116	125
TNFRSF11 B	O00300	3	3	48	130	118	93	41	42	45	168	148	144	120	125	153
TNIK	F5H5M9	18	4	239	76	23	58	76	87	96	69	60	64	58	62	76
TNIP1	E7ET96	12	5	144	39	28	36	53	64	60	105	71	74	70	73	73
TNKS1BP1	Q9C0C2	38	30	907	39357	631	648	7193	2319	2481	3889	2177	2529	3480	2344	3630
TNPO1	Q92973	16	11	523	1079	322	383	272	136	166	317	442	280	232	211	416
TNPO2	O14787-2	9	3	285	1830	18	22	223	10	3	50	10	4	25	10	2
TNPO3	C9J7E5	9	3	139	19	25	18	8	4	4	12	17	10	8	7	7
TNS3	E7ERH3	18	9	376	85	65	47	857	795	670	279	211	285	235	293	233
TOE1	Q96GM8	6	2	112	0	5	3	3	9	6	10	10	2	1	1	2
TOMM34	Q15785	7	3	35	39	11	44	5	4	12	14	27	12	9	14	11
TOP1	P11387	32	11	618	1996	145	230	352	103	101	791	955	507	631	413	717
TOP2A	P11388	35	18	813	412	599	395	656	767	948	594	529	590	623	523	561
TOP2B	Q02880	43	16	1030	460	272	274	306	368	427	727	676	635	664	598	677
TOR1AIP1	Q5JTV8	13	7	287	119	77	124	62	57	95	299	260	267	268	196	291
TOX4	B4DPY8	3	2	52	3	2	9	3	2	0	5	13	6	2	3	4
TP53	P04637	13	6	371	155	310	348	73	63	170	80	99	127	80	92	91
TP53BP1	F8VY86	26	13	681	133	64	61	29	19	20	339	339	185	202	152	247
TP53BP2	Q13625	15	6	249	10	11	6	76	89	59	158	159	116	94	129	105
TP53I3	Q53FA7	6	3	95	36	26	23	12	9	19	15	36	31	12	6	30
TPI1	P60174	13	4	660	2526	145	990	515	210	297	513	592	449	643	519	551
TPM1	H0YKP3	16	2	756	12079	3078	13415	3898	4275	5526	2559	1558	2779	4564	3922	5688

TPM4	P67936	26	3	1005	56	155	172	1345	1318	1544	1096	1135	1213	1622	1250	1612
TPR	P12270	105	63	3597	8557	3575	4210	1525	975	1436	3417	3356	2126	2300	1910	2571
TRA2A	B4DQI6	8	5	242	144	118	116	52	53	77	74	63	59	62	80	56
TRA2B	H7BXF3	10	2	311	53	43	64	417	333	496	613	601	723	652	587	619
TRA2B	P62995	12	2	470	409	259	619	318	304	178	361	408	304	309	355	312
TRAF1	Q13077	11	5	292	156	68	72	336	274	339	151	113	133	142	125	114
TRAP1	F5H897	5	2	140	14	0	1	1	1	1	1	3	1	2	0	2
TRAPPC3	A6NDN0	3	2	102	19	18	43	40	22	48	44	55	61	47	55	41
TRAPPC9	Q96Q05-2	10	4	51	16	62	63	135	133	156	260	234	316	272	270	308
TRIM14	Q14142	6	2	54	3	8	4	6	4	5	5	6	15	4	10	5
TRIM21	F5H012	8	2	88	27	63	20	33	11	8	56	59	79	61	62	40
TRIM22	Q8IYM9	14	8	351	241	168	121	933	826	1461	594	305	393	490	435	569
TRIM25	Q14258	17	14	624	590	123	175	722	736	941	792	750	962	964	941	1156
TRIM28	Q13263	29	18	939	640	734	1092	256	284	386	1597	1932	1130	1155	866	1397
TRIM33	H0Y612	5	5	105	20	21	3	87	2672	58	30	991	50	71	1007	45
TRIM56	Q9BRZ2	6	5	41	131	12	15	25	89	21	42	89	40	34	99	33
TRIO	O75962	38	17	374	1334	378	498	680	474	514	582	636	673	523	526	562
TRIOBP	F8W6V6	25	8	651	703	138	155	631	432	470	454	445	462	477	449	554
TRIP11	Q15643	35	15	506	9670	21988	14376	113478	116874	139694	47021	45917	50070	59154	51930	63888
TRIP12	Q14669	17	8	233	73	73	126	39	61	32	120	153	98	81	87	95
TRIP6	Q15654	11	7	197	193	97	97	878	754	609	456	386	392	461	354	393
TRMT1	Q9NXH9	8	4	201	54	12	14	12	5	3	12	35	44	14	5	16
TRRAP	F2Z2U4	49	29	785	607	543	493	495	608	679	633	645	645	620	543	675
TSEN54	J9JIH8	6	3	26	122	367	238	1600	1131	2220	994	1062	1320	1381	1197	1953
TSG101	F5H442	8	4	150	74	16	56	87	86	98	167	149	193	195	171	178
TSN	H7C1D4	7	2	78	12	12	4	34	19	2	60	60	61	50	67	50
TSPYL1	Q9H0U9	5	3	57	26	18	33	9	10	13	21	22	25	20	10	19
TSR1	Q2NL82	8	4	167	33	61	43	16	25	33	76	98	64	66	42	74
TSSC1	D3YTH5	3	2	22	1	1	2	2	0	1	15	28	16	30	24	1
TSTA3	Q13630	4	3	121	48	24	20	9	14	22	11	20	17	10	16	14
TTC37	Q6PGP7	13	5	244	4	4	2	8	8	7	32	30	26	10	13	19
TTN	Q8WZ42	247	38	1205	951	982	795	803	652	808	989	866	774	1338	730	905
TUBA1C	F5H5D3	32	4	1642	1192	1553	2327	278	235	454	445	552	556	386	532	381
TUBA4A	A8MUB1	31	3	1437	114	182	169	30	42	43	183	198	182	234	161	119
TUBB	P07437	45	6	2253	2971	5169	5708	2163	2418	3213	3583	3505	4369	3901	3405	4196
TUBB3	Q13509	30	3	1525	77	44	70	15	10	17	54	46	85	69	52	60
TUBB6	Q9BUF5	24	4	1078	306	242	461	63	39	34	74	80	99	84	69	75
TUBG2	Q9NRH3	7	5	193	150	71	74	114	103	66	215	275	241	166	199	151
TUBGCP3	Q96CW5	13	10	432	183	84	84	144	103	115	210	228	215	165	161	176
TUBGCP4	Q9UGJ1	7	2	78	12	15	5	12	20	21	16	13	25	21	18	20
TUFM	P49411	9	5	170	42	66	119	70	74	103	138	128	189	167	151	168
TWF1	Q12792	9	5	145	61	60	75	79	71	85	117	145	149	117	97	113
TWF2	Q6IBS0	9	6	167	87	67	71	169	159	176	277	298	334	279	292	304
TXLNA	P40222	13	2	198	25	0	5	46	29	16	79	57	66	47	52	49
TXN	P10599	5	5	158	277	288	446	374	371	365	372	399	450	584	432	550
TXNL1	K7ER96	3	2	100	8	6	36	11	12	19	23	26	19	26	17	18
TXNL4A	K7EJU2	2	2	38	21	5	13	12	7	9	16	25	18	14	13	20

TXNRD1	E7ESI6	16	11	182	152	125	297	333	269	257	482	539	553	407	360	529
U2AF1	Q01081	5	3	229	49	212	138	185	194	110	448	515	314	452	429	365
U2AF2	P26368	14	6	514	426	339	568	242	266	345	730	879	691	556	505	807
U2SURP	O15042	20	7	550	335	691	279	321	255	464	215	174	190	228	187	223
UACA	H0YNH8	19	5	213	37	12	19	94	109	162	79	117	106	76	56	82
UAP1	Q16222	6	5	111	155	20	31	42	40	48	22	20	25	27	29	22
UBA1	P22314	24	11	725	277	314	513	127	117	128	405	484	435	345	297	367
UBA6	A0AVT1	15	8	235	185	181	194	165	146	173	473	445	493	519	491	495
UBAP2L	F8W726	11	8	299	98	148	125	89	91	79	319	355	301	303	254	274
UBE2I	H3BPC4	8	4	115	168	315	239	83	103	164	152	207	175	230	155	199
UBE2N	F8VSD4	4	3	67	69	87	113	22	17	20	43	47	59	46	41	37
UBE2V2	H0YBX6	5	2	59	55	74	107	16	20	25	14	18	15	24	16	23
UBP1	Q9NZI7	3	2	24	3	9	14	4	3	22	2	11	12	7	12	6
UBR4	Q5T4S7	39	9	361	412	187	139	2059	1798	2730	1148	1043	1419	1659	1372	2031
UBR5	O95071	14	6	104	165	159	258	210	275	244	276	237	334	313	307	336
UBTF	E9PKP7	30	20	472	252	386	361	582	432	160	624	693	493	553	528	502
UBXN1	E9PJ81	5	2	37	65	35	262	9	9	5	35	16	20	102	17	24
UBXN7	O94888	7	2	63	4	3	3	5	13	3	11	16	8	2	7	9
UCHL1	P09936	10	6	237	652	430	1170	258	239	336	265	288	347	342	293	321
UFD1L	A6NJ11	4	2	63	30	9	51	38	41	51	57	65	79	82	85	117
UGDH	O60701	11	6	237	130	222	274	86	69	102	196	189	226	215	184	198
UHRF1	Q96T88	16	9	417	125	71	56	21	20	47	25	26	24	21	22	27
UNC45A	Q9H3U1-2	21	9	276	67	36	46	277	218	220	181	199	191	167	174	156
Uncharacterized	I3L521	3	2	106	14	3	1	0	1	4	6	18	9	5	4	9
UPF1	Q92900	25	16	466	319	183	233	302	245	262	501	560	641	570	498	602
UPI000013FC70	H7BZT4	4	2	105	6	10	9	39	34	20	4	90	15	70	49	95
UQCRC1	P31930	12	8	293	655	80	183	248	172	168	108	117	115	143	124	147
UQCRC2	P22695	9	2	293	44	4	80	3	5	7	7	16	14	21	10	19
UQCRQ	O14949	2	2	20	40	26	13	70	37	47	83	39	57	69	56	41
URB1	O60287	29	12	474	937	82	50	136	112	49	76	97	76	67	40	52
URB2	Q14146	16	8	240	65	39	43	5	6	13	29	43	46	19	15	19
USO1	O60763	15	9	506	162	93	154	45	63	48	203	216	254	225	178	194
USP10	Q14694	8	3	71	158	40	73	76	57	62	130	123	129	135	127	138
USP11	G5E9A6	6	2	59	21	2	5	5	8	2	39	6	7	7	4	4
USP14	J3QRZ5	8	2	205	49	9	14	13	6	6	10	8	7	7	6	3
USP15	E9PCQ3	7	3	111	18	5	14	18	11	9	15	19	21	13	17	13
USP24	B7WPF4	11	2	94	558	1432	976	7506	6954	8022	4139	4118	4661	4920	4717	5614
USP34	Q70CQ2	20	4	144	24	339	57	1734	62	58	45	28	24	116	38	25
USP36	Q9P275	11	2	93	9	24	10	5	4	7	7	12	10	6	4	12
USP39	B9A018	12	9	343	334	93	171	108	75	75	335	454	254	273	245	319
USP48	B7ZKS7	11	5	152	491	3	11	63	4	7	20	28	9	21	6	9
USP5	P45974	6	4	114	36	42	47	8	13	15	33	32	47	18	11	67
USP7	F5H8E5	15	7	159	57	41	67	31	32	42	49	76	38	37	34	60
USP9X	Q93008	33	11	650	88	57	120	128	96	106	259	293	280	244	215	274
UTP11L	Q9Y3A2	8	5	90	48	37	26	21	16	34	32	56	31	28	27	39

UTP14A	Q9BVJ6	18	3	516	29	13	7	5	1	11	24	29	8	10	10	12
UTP18	Q9Y5J1	12	9	251	412	166	130	106	71	72	152	180	143	132	104	177
UTP20	O75691	43	19	726	605	191	172	143	58	107	132	177	116	121	116	98
UTP3	Q9NQZ2	4	2	77	29	2	6	6	14	2	19	28	15	10	10	27
UTP6	Q9NYH9	15	10	304	109	111	98	525	584	793	297	309	288	293	321	351
UTRN	P46939	82	29	1614	801	1633	1189	2633	2880	3579	2351	1686	2415	2342	2860	2235
VARS	B0V043	35	24	904	734	352	492	377	294	378	723	744	845	714	570	799
VASP	P50552	17	14	717	255	389	471	5592	5199	6649	2949	2774	3538	3625	3413	3762
VAT1	Q99536	6	3	176	16	69	144	98	105	125	166	107	139	144	175	166
VCAN	P13611	19	11	407	1587	1802	1359	1655	1618	1891	528	302	430	464	532	432
VCL	P18206	69	40	2826	6435	1959	2673	23267	20614	24173	10934	8196	9551	10992	11324	11107
VCP	P55072	39	26	1311	1600	1919	2629	1047	1122	1534	2031	2479	2083	1580	1513	1912
VCPIP1	Q96JH7	8	4	86	20	19	54	2	5	4	26	24	18	14	9	21
VDAC1	P21796	9	6	338	780	508	888	134	159	213	131	144	173	133	130	117
VDAC2	P45880	8	7	278	496	343	996	125	101	175	147	162	217	181	147	180
VDAC3	F5H740	3	2	147	22	5	24	9	3	9	8	7	10	1	1	10
VIM	P08670	83	59	5306	360297	578949	636434	137829	123408	188736	312518	282733	343806	359354	332240	368291
VPRBP	Q9Y4B6	9	4	37	49	165	177	600	544	876	523	481	528	611	543	570
VPS13C	Q709C8	28	5	199	35	108	146	173	159	194	314	335	232	289	217	238
VPS26A	O75436	14	3	272	106	355	139	135	78	94	190	121	86	232	105	113
VPS28	E9PQR7	4	3	47	1256	27	11	105	16	10	43	29	28	27	18	9
VPS29	H3BLV8	5	5	96	73	47	77	23	18	18	21	38	49	24	21	25
VPS33A	Q96AX1	7	4	127	5	8	4	60	69	76	41	39	43	42	35	41
VPS33B	Q9H267	8	4	92	9	29	32	158	142	160	88	104	108	150	143	135
VPS35	Q96QK1	22	11	429	187	123	310	407	346	299	487	465	558	520	475	598
VPS4B	O75351	5	2	72	18	15	17	9	11	8	22	51	47	40	26	40
VPS51	Q9UID3	8	2	54	51	1	12	15	13	13	18	15	10	19	26	4
VRK1	Q99986	6	4	143	213	90	370	69	93	80	94	121	116	118	119	156
VWA9	B4DJL6	6	4	58	76	8	4	9	0	3	18	20	20	16	16	27
WAPAL	Q7Z5K2-3	14	5	128	215	50	159	54	55	31	89	80	71	84	66	88
WARS	P23381	6	4	75	234	119	216	137	109	193	188	174	222	205	168	214
WASF2	Q9Y6W5	6	5	143	20	14	28	57	49	42	202	128	171	119	114	132
WBP11	F5H721	5	3	104	30	46	69	8	15	26	62	62	48	33	46	47
WDHD1	O75717	17	7	266	644	52	34	27	8	27	12	21	20	18	14	11
WDR1	O75083	25	7	540	393	156	202	783	744	245	502	608	548	504	475	440
WDR11	Q9BZH6	8	3	79	17	3	9	18	20	9	19	18	19	20	13	26
WDR12	Q9GZL7	15	10	274	243	88	146	60	50	88	167	268	160	159	143	169
WDR18	Q9BV38	14	4	368	85	60	55	17	21	23	210	230	170	166	153	213
WDR26	Q9H7D7	6	4	119	110	49	80	95	12	79	42	76	113	28	52	248
WDR3	Q9UNX4	18	11	403	836	180	245	121	55	106	97	167	101	87	61	67
WDR33	Q9C0J8	20	11	195	316	51	73	120	58	55	141	212	97	117	97	80
WDR36	Q8NI36	20	15	506	424	133	130	71	30	38	216	280	170	152	105	182
WDR43	Q15061	15	10	402	87	91	395	22	17	304	95	171	142	56	76	230
WDR46	O15213	15	5	171	22	12	74	70	63	78	142	168	141	141	113	143
WDR5	P61964	9	7	275	70	100	191	29	48	46	129	167	113	91	79	106
WDR61	Q9GZS3	9	7	272	113	57	44	112	74	84	82	144	86	77	79	70
WDR70	Q9NW82	4	2	99	37	8	10	33	27	36	31	24	25	29	34	33

WDR75	Q8IWA0	13	10	309	179	95	126	44	22	38	87	134	74	86	66	76
WDR77	Q9BQA1	3	2	121	34	5	28	21	15	14	37	43	35	23	31	31
WDR82	Q6UXN9	12	7	203	55	242	53	1500	78	137	149	216	157	209	118	197
WHSC1	O96028	12	3	170	78	26	32	32	34	48	41	39	39	44	40	46
WHSC2	B3KSP0	7	4	168	121	8	8	13	0	12	18	33	22	13	12	13
WIZ	O95785	9	3	80	4	17	14	9	11	12	23	14	14	9	7	19
WTAP	Q15007	8	6	238	26	38	39	11	9	14	82	55	38	53	23	39
XAB2	F5H315	23	19	614	874	348	396	207	124	157	198	275	204	204	175	252
XP32	Q5T750	3	2	61	489	463	235	167	289	204	127	42	62	55	63	62
XPC	Q01831	4	2	97	455	266	298	69	46	69	66	59	64	81	37	81
XPO1	O14980	42	24	1406	1960	1137	1477	626	468	678	875	1075	803	723	625	774
XPO4	F2Z2X4	8	3	158	186	3	13	22	2	3	7	10	8	5	5	10
XPO5	Q9HAV4	20	12	618	147	70	157	34	55	86	131	182	138	89	80	128
XPO7	E7ESC6	8	3	82	34	13	3	4	1	0	4	7	3	0	0	3
XPOT	O43592	11	5	150	180	135	122	110	86	82	124	192	210	120	146	103
XRCC1	P18887	9	5	116	12	9	16	18	27	21	40	88	66	47	40	70
XRCC4	Q13426	5	3	27	37	15	25	27	22	10	32	34	31	30	38	57
XRCC5	P13010	30	20	874	1247	1283	1856	625	262	576	1925	2021	1909	1151	1361	1232
XRCC6	P12956	42	33	1247	4063	2456	3448	1234	628	1277	2932	3451	3052	2326	2886	3079
XRN1	Q8IZH2	11	3	178	0	7	1	19	18	31	37	44	45	46	29	44
XRN2	B4DZC3	27	18	597	375	260	402	355	341	333	1143	1262	923	912	823	1219
YARS	P54577	13	5	229	203	180	208	103	74	84	93	102	134	84	92	126
YLPM1	P49750-4	18	11	307	312	921	487	164	104	141	312	307	148	215	216	165
YME1L1	Q96TA2	13	2	121	51	19	21	8	7	12	9	11	14	3	12	14
YTHDC1	J3QR07	13	2	164	17	10	10	9	0	27	51	68	71	65	50	111
YWHAB	P31946	19	5	739	1027	395	803	829	975	1163	696	880	1291	1302	1066	1466
YWHAE	P62258	20	9	786	1373	1759	2030	1707	1499	1589	1252	1790	1920	1956	2244	1939
YWHAG	P61981	17	5	521	70	15	86	189	159	138	111	77	167	130	123	188
YWHAH	Q04917	17	6	566	432	401	746	648	503	432	389	592	532	592	600	551
YWHAQ	P27348	21	9	787	221	710	968	1130	1062	1025	1174	1086	1022	1562	1243	1208
YWHAZ	P63104	24	10	812	2796	2376	3143	3072	2939	2319	3997	3834	3737	5356	4617	4227
ZC3H11A	O75152	8	3	148	160	77	141	488	620	852	321	170	311	376	433	504
ZC3H13	Q5T200	15	6	85	94	89	59	196	240	211	109	86	126	169	132	165
ZC3H14	H0YJA2	10	6	271	12	1	33	3	12	16	53	47	35	34	25	29
ZC3H18	E7ERS3	10	2	84	1	2	3	4	1	1	6	6	3	3	12	4
ZC3H7B	Q9UGR2	3	2	25	3	4	0	29	3	11	7	13	15	18	5	3
ZC3HC1	C9J0I9	12	11	205	917	1634	1499	1354	1259	2001	1019	907	1047	1655	1497	2068
ZCCHC8	Q6NZY4	5	2	111	36	44	23	5	10	3	28	32	20	20	16	22
ZFR	Q96KR1	22	14	368	1057	155	132	66	26	64	231	438	189	165	122	243
ZFYVE16	Q7Z3T8	12	3	66	346	19	141	61	69	74	93	95	110	122	129	114
ZMYM2	Q9UBW7	11	6	100	632	35	26	126	143	92	165	194	201	200	154	205
ZMYM3	A6NHB5	9	2	51	5	9	6	2	4	8	13	6	6	4	7	5
ZMYM4	Q5VZL5	9	3	101	21	10	21	54	45	46	45	39	56	55	41	51
ZMYND8	Q2HXV1	11	5	131	108	32	6	45	4	63	47	60	46	39	28	41
ZNF185	O15231-3	25	10	621	181	223	166	1839	1652	2248	1134	879	1095	1197	1030	1217
ZNF207	J3QRS9	2	2	38	12	32	33	13	35	35	91	79	79	85	74	97
ZNF233	A6NK53	4	2	23	515	11	8	81	27	5	31	51	35	23	31	23

ZNF281	A6NF48	7	2	109	84	2	17	24	12	15	11	3	2	9	5	4
ZNF326	Q5BKZ1	15	7	542	49	9	18	28	40	21	267	312	167	186	136	251
ZNF469	H3BS19	23	7	111	66	38	58	128	111	141	214	229	237	226	212	288
ZNF638	Q14966	19	2	318	18	1	1	0	13	21	6	11	14	20	3	1
ZNF687	H0Y5I5	7	4	52	57	29	35	318	339	516	219	258	234	381	309	308
ZNF800	Q2TB10	7	5	95	8	16	1255	13	23	11	26	41	38	30	35	33
ZNFX1	Q9P2E3	17	2	102	24	17	28	4	2	3	7	11	4	7	7	7
ZW10	O43264	10	4	211	14	9	12	21	10	7	13	21	20	11	12	19
ZWILCH	Q9H900	5	3	88	18	0	10	11	11	9	21	32	28	19	13	10
ZYX	B4DQX7	8	5	260	209	140	136	533	584	692	495	470	517	678	609	640
